# Reactions of *N*,3-diarylpropiolamides with arenes under superelectrophilic activation: synthesis of 4,4-diaryl-3,4-dihydroquinolin-2(1*H*)-ones and their derivatives

**DOI:** 10.3762/bjoc.12.93

**Published:** 2016-05-11

**Authors:** Larisa Yu Gurskaya, Diana S Belyanskaya, Dmitry S Ryabukhin, Denis I Nilov, Irina A Boyarskaya, Aleksander V Vasilyev

**Affiliations:** 1 Department of Organic Chemistry, Institute of Chemistry, Saint Petersburg State University, Universitetskaya nab., 7/9, Saint Petersburg, 199034, Russia; 2 N.N. Vorozhtsov Novosibirsk Institute of Organic Chemistry, Siberian Branch of Russian Academy of Science, ul. Lavrentieva 9, Novosibirsk, 630090, Russia; 3 Department of Chemistry, Saint Petersburg State Forest Technical University, Institutsky per. 5, Saint Petersburg, 194021, Russia

**Keywords:** alkynes, quinolinones, Friedel–Crafts reactions, superacids, superelectrophilic activation

## Abstract

The reaction of 3-aryl-*N*-(aryl)propiolamides with arenes in TfOH at room temperature for 0.5 h led to 4,4-diaryl-3,4-dihydroquinolin-2-(1*H*)-ones in yields of 44–98%. The obtained dihydroquinolinones were further transformed into the corresponding *N*-acyl or *N*-formyl derivatives. For the latter, the superelectrophilic activation of the *N*-formyl group by TfOH in the reaction with benzene resulted in the formation of *N*-(diphenylmethyl)-substituted dihydroquinolinones.

## Introduction

Quinoline derivatives are a very important class of heterocycles, which are used in chemistry, biology, medicine, and materials science. For instance, see a series of recent reviews on anti-malaria drugs containing a quinoline motif in the structure [1–3]. The synthesis of quinolines is an important task of organic chemistry [4–5]. Many of these synthetical protocols are based on alkynes, which are widely used for the preparation of various carbo- and heterocycles [6–10].

Based on our works on the synthesis of 4-arylquinolin-2(1*H*)-ones from acetylene compounds under superelectrophilic activation conditions [11–14], we continued to develop some methods for the syntheses of quinoline derivatives. Previously we showed, just in a few examples, that some 3-aryl-*N*-(aryl)propiolamides reacted with benzene under the action of Brønsted or Lewis superacids affording 4-aryl-4-phenyl-3,4-dihydroquinolin-2(1*H*)-ones [12–13].

The main goal of this work was a systematic study on reactions of 3-aryl-*N*-(aryl)propiolamides with arenes under the action of the Brønsted superacid TfOH (CF_3_SO_3_H, triflic acid), strong Lewis acids AlX_3_ (X = Cl, Br), or the conjugate Brønsted–Lewis superacid TfOH–SbF_5_.

## Results and Discussion

**Scheme 1:**
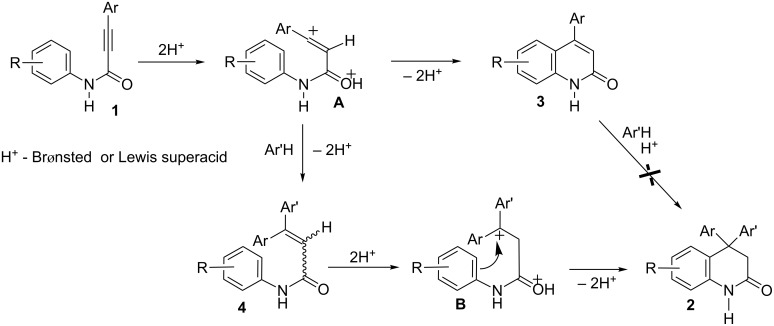
Transformations of 3-aryl-*N*-(aryl)propiolamides **1** into 4-arylquinolin-2(1*H*)-ones **3** or 4,4-diaryl-3,4-dihydroquinolin-2(1*H*)-ones **2** in the presence of arenes through the formation of intermediate cations **A**, **B** under the superelectrophilic activation.

The protonation of alkynamides **1** on both the oxygen atom of the amide group and a carbon atom of the acetylene bond in superacids or coordination of these basic centers with strong Lewis acids leads to the formation of dications **A** that are considered as superelectrophiles [15] (Scheme 1). These dications can either undergo an intramolecular cyclization to 4-arylquinolin-2(1*H*)-ones **3** or, alternatively, react with arenes as external π-nucleophiles. In this latter pathway, Friedel–Crafts alkenylation of arenes by species **A** leads to structures **4**, which can then be diprotonated to the cations **B** and finally cyclized to 4,4-diaryl-3,4-dihydroquinolin-2(1*H*)-ones **2**. It should be noted that quinolinones **3** do not react with arenes with formation of compounds **2** under superelectrophilic activation, and starting materials **3** remain unreacted [12–13].

Such 4,4-diaryl-3,4-dihydroquinolin-2-(1*H*)-ones **2** are very rare objects, there is no general method for their synthesis [16–17]. Thus, developing of synthetic method to get these compounds is an actual goal of organic chemistry.

**Figure 1:**
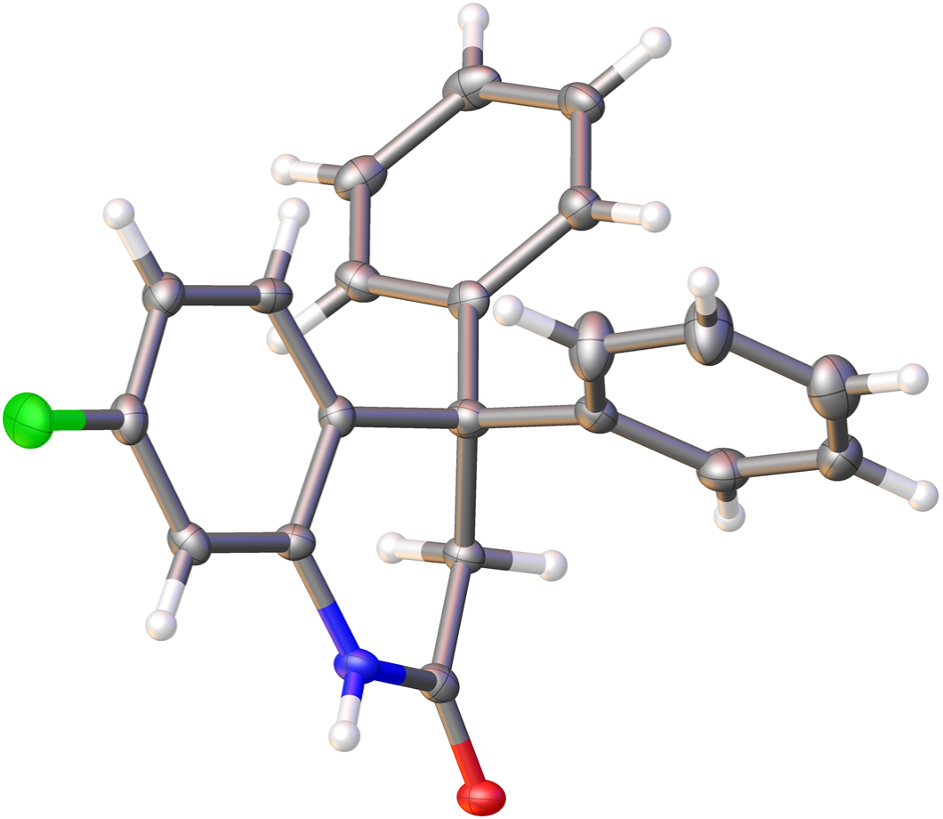
Molecular structure of **2f** (ellipsoid contours of probability levels are 50%).

Reactions of the series of 3-aryl-*N*-(aryl)propiolamides **1a–u**, bearing various donor–acceptor substituents on aryl rings, with benzene and other arenes in the presence of TfOH, TfOH–SbF_5_, AlCl_3_, or AlBr_3_ were studied. Initial amides **1a–u**, reaction conditions, target 4,4-diaryl-3,4-dihydroquinolin-2-(1*H*)-ones **2a–x**, and some byproducts **3a–c** and intermediate compounds **4a**,**b** are given in Table 1. Structures of compounds **2a–x**, **3a–c**, and **4a**,**b** were determined by means of ^1^H, ^13^C, ^19^F NMR, HRMS methods (see Supporting Information File 1), and X-ray analysis in case of compound **2f** (Figure 1).

**Table 1:** Reactions of amides **1a–u** with benzene (and other arenes) under superelectrophilic activation, leading to dihydroquinolinones **2a–x**.

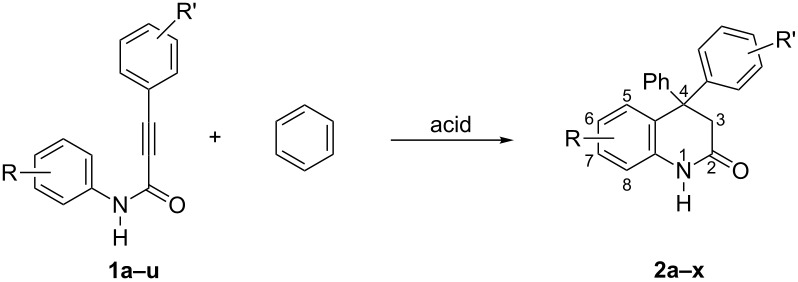								

Entry	Starting amide **1**			Acid (reaction conditions)	Reaction product, dihydroquinolinone **2**			
	no.	R	R′		no.	R	R′	Yield, %

1	**1a**	H	H	TfOH^a^	**2a**	H	H	90
2	**1a**			TfOH-SbF_5_^a^	**2a**			85
3	**1a**			AlCl_3_^b^	**2a**			91
4	**1a**			AlBr_3_^b^	**2a**			52^c^
5	**1b**	2-Me	H	TfOH^a^	**2b**	8-Me	H	93
6	**1b**			TfOH-SbF_5_^a^	**2b**			38
7	**1c**	3-Me	H	TfOH^a^	**2c**	7-Me	H	75
8	**1c**			TfOH-SbF_5_^a^	**2c**			83
9	**1d**	4-Me	H	TfOH^a^	**2d**	6-Me	H	98
10	**1d**			TfOH-SbF_5_^a^	**2d**			53
11	**1e**	2-F	H	TfOH^a^	**2e**	8-F	H	73
12	**1e**			TfOH-SbF_5_^a^	**2e**			95
13	**1e**			AlBr_3_^b^	**2e**			55
14	**1f**	3-F	H	TfOH^a^	**2f**	7-F	H	52
15	**1f**			AlBr_3_^b^	**2f**			58
16	**1g**	4-F	H	TfOH^a^	**2g**	6-F	H	18^d^
17	**1g**			TfOH^e^	**2g**			84
18	**1g**			TfOH-SbF_5_^a^	**2g**			95
19	**1g**			AlBr_3_^b^	**2g**			72
20	**1h**	4-Cl	H	TfOH^a^	**2h**	6-Cl	H	23^f^
21	**1h**			TfOH^e^	**2h**			88
22	**1h**			AlBr_3_^b^	**2h**			52
23	**1i**	2,3-Me_2_	H	TfOH^a^	**2i**	7,8-Me_2_	H	88
24	**1j**	2,4-Me_2_	H	TfOH^a^	**2j**	6,8-Me_2_	H	89
25	**1k**	3,4-Me_2_	H	TfOH^a^	**2k**	6,7-Me_2_	H	74
26	**1l**	2-MeO	H	TfOH^a^	**2l**	8-MeO	H	98
27	**1m**	3-MeO	H	TfOH^a^	**2m**	7-MeO	H	44
28	**1n**	4-MeO	H	TfOH^a^	**2n**	6-MeO	H	64
29	**1o**	3-F, 4-MeO	H	TfOH^a^	**2o**	7-F, 6-MeO	H	53
30	**1p**	H	4-Me	TfOH^a^	**2p**	H	4-Me	90
31	**1q**	4-F	4-Me	TfOH^a^	**2q**	6-F	4-Me	68
32	**1q**			AlBr_3_^b^	**2q**			53
					**2g**	6-F	H	13
33	**1r**	H	4-F	TfOH^a^	**2r**	H	4-F	66
34	**1s**	4-F	4-Cl	TfOH^a^	**2s**	6-F	4-Cl	87
35	**1s**			AlBr_3_^b^	**2s**			70
36	**1t**	2,3-benzo	H	AlBr_3_^b^	**2t**	7,8-benzo	H	22^g^
37	**1u**	3,4-benzo	H	AlBr_3_^b^	**2u**	5,6-benzo	H	17^h^
38	**1a**	H	H	TfOH^a,i^	**2v**	H	4-Cl	50
39	**1a**			TfOH^a,j^	**2x**	H	3,4-Cl_2_	60

^a^Room temperature, 0.5 h. ^b^80 °C, 1 h. ^c^4-Phenylquinolin-2(*1H*)-one **3a** was also obtained in a yield of 41%. ^d^*N*-(4-Fluorophenyl)amide of 3,3-diphenylpropenoic acid **4a** [Ph_2_C=CHCONH(4-FC_6_H_4_)] was obtained as a major reaction product in a yield of 80%. ^e^Room temperature, 7 h. ^f^*N*-(4-Chlorophenyl)amide of 3,3-diphenylpropenoic acid **4b** [Ph_2_C=CHCONH(4-ClC_6_H_4_)] was obtained as a major reaction product in a yield of 75%. ^g^4-Phenyl-7,8-benzoquinolin-2(*1H*)-one **3b** was also obtained in a yield of 15%. ^h^4-Phenyl-5,6-benzoquinolin-2(*1H*)-one **3c** was also obtained in a yield of 17%. ^i^Reaction with chlorobenzene. ^j^Reaction with 1,2-dichlorobenzene.

We found that the best conditions for the synthesis of dihydroquinolines **2** from amides **1** and arenes were as follows: TfOH, room temperature, 0.5 h. Some optimization of the reaction on minimization of the usage of arenes and TfOH were performed. The amount of arene may be decreased to 5 equivalents. But, concerning the use of a lower amount of TfOH, for instance, running the reaction in CH_2_Cl_2_ solution led to a dramatic decrease of the yields of the target products. Thus, it is better to conduct the reaction in neat TfOH, which is a good weak nucleophilic medium for the stabilization of the intermediate cationic species.

No concurrent formation of the corresponding 4-arylquinolinones **3** was observed, and in most cases the yields of the target products **2** are 44–98% (see Table 1). Also this reaction may be carried out under the action of other acids, e.g., TfOH–SbF_5_ or AlX_3_ (X = Cl, Br). But in the stronger acidic system TfOH–SbF_5_ (Hammet acidity function H_0_ −19, see [18]) the yields of compounds **2** were usually lower as compared to TfOH (H_0_ −14) (Table 1, entries 2, 6 and 10). For the Lewis acid AlBr_3_, additional formation of compounds **3a**,**b**,**c** was detected (Table 1, entries 4, 36 and 37), or exchange of aryl groups in **2** took place (Table 1, entry 32, formation of **2g** from **2q**, as a result of the exchange of the *p*-tolyl moiety by a phenyl group).

Amides **1**, bearing various donor (Me, MeO, benzo-) and acceptor (F, Cl) substituents on both aryl rings at the acetylene bond and at the nitrogen atom may be involved in this reaction. Substrates **1g**, **1h**, having electron withdrawing 4-F and 4-Cl substituents on the *N*-aryl ring, were hardly cyclized under the formation of dihydroquinolinones **2g**, **2h**. Thus, at room temperature for 0.5 h, apart from target compounds **2g**, **2h**, the corresponding amides of 3,3-diphenylpropenoic acid **4a**, **4b** were isolated (Table 1, entries 16 and 20). Increasing the reaction time to 7 h resulted in the solely formation of **2g** and **2h** (Table 1, entries 17 and 21). That clearly proved the participation of compounds **4** in the reaction pathway leading to dihydroquinoliones **2** (Scheme 1).

It should be noted, that substrates **1** with more than one possible reaction site, namely 3-substituted amides **1c**,**f** and 3,4-disubstituted amides **1k**,**o** gave regioselectively only 7-substituted quinolinones **2c**,**f** (Table 1, entries 7, 8, 14 and 15) and 6,7-disubstituted quinolinones **2k**,**o** (Table 1, entries 25 and 29), respectively.

Concerning the arene component of this reaction, apart from benzene, chlorobenzene and 1,2-dichlorobenzene it may take part in this Friedel–Crafts process (Table 1, entries 38 and 39). More donating aromatic substrates, such as toluene, isomeric xylenes, mesitylene, or pseudocumene, led to complex mixtures of oligomeric reaction products. Such activated aryl groups may undergo several electrophilic attack from intermediate cationic species **A** and **B** (Scheme 1), that complicated this reaction.

Thus, reaction of amides **1** with benzene and some other arenes in TfOH is an effective way to 4,4-diaryl-3,4-dihydroquinolin-2(1*H*)-ones **2**.

**Scheme 2:**
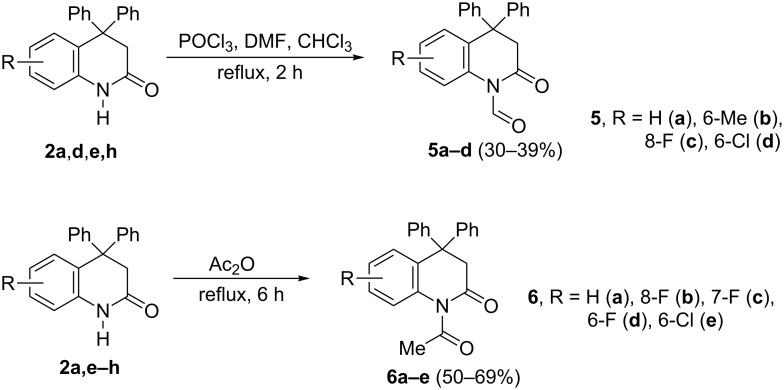
*N*-Formylation and *N*-acylation of dihydroquinolinones **2**.

To show the synthetic potential of dihydroquinolinones **2** their *N*-formylation and *N*-acetylation reactions were carried out. Compounds **2a**,**d**,**e** and **h** were *N*-formylated in the system POCl_3_–DMF–CHCl_3_ under the Vilsmeier–Haack reaction conditions with formation of compounds **5a–d** (Scheme 2). Apart from that, dihydroquinolinones **2a**,**e–h** were acetylated into derivatives **6a–e** (Scheme 2).

**Scheme 3:**
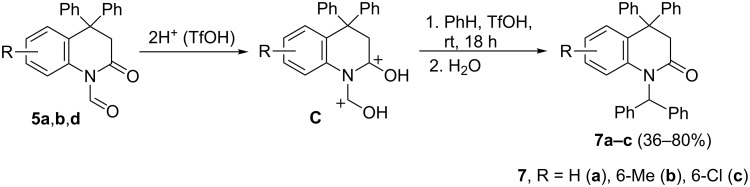
Superelectrophilic activation of the *N*-formyl group of compounds **5** and their reaction with benzene.

Then reactions of compounds **5** and **6** with arenes in TfOH were checked. Analogously to other heteroaromatic aldehydes of the series of pyridine [19], quinolone [20], pyrazol [21], and imidazole [22], the superelectrophilic activation of the formyl group in **5** was expected. Indeed, compounds **5a**,**b** and **d** reacted with benzene in a Friedel–Crafts process affording *N-*diphenylmethyl substituted derivatives **7a–c**, respectively (Scheme 3). Most probably, the reaction proceeds through the formation of O,O’-diprotanated intermediate **C** (Scheme 3), which are considered as superlectrophiles [15]. The close structural proximity of two positive charges in **C** substantially increases the electrophilic properties of the carbon of the O-protonated *N*-formyl group and explains its reactivity. The reactions of **5** with other arenes, such as 1,2-dichlorobenzene, toluene, or isomeric xylenes, gave deformylation products **2**. In this case, probably, target compounds **7** were initially formed, but then protolytic cleavage of the N–CH(Ar)_2_ bond occurred leading to relatively stable diarylmethyl cations (^+^CH(Ar)_2_). That took place to less extent also for the *N-*diphenylmethyl group in **7a–c**. It should be mentioned, that this is the first example of such a superelectrophilic activation of a *N-*formyl group, and this reaction did not take place for *N*-acyl derivatives **6**.

**Figure 2:**
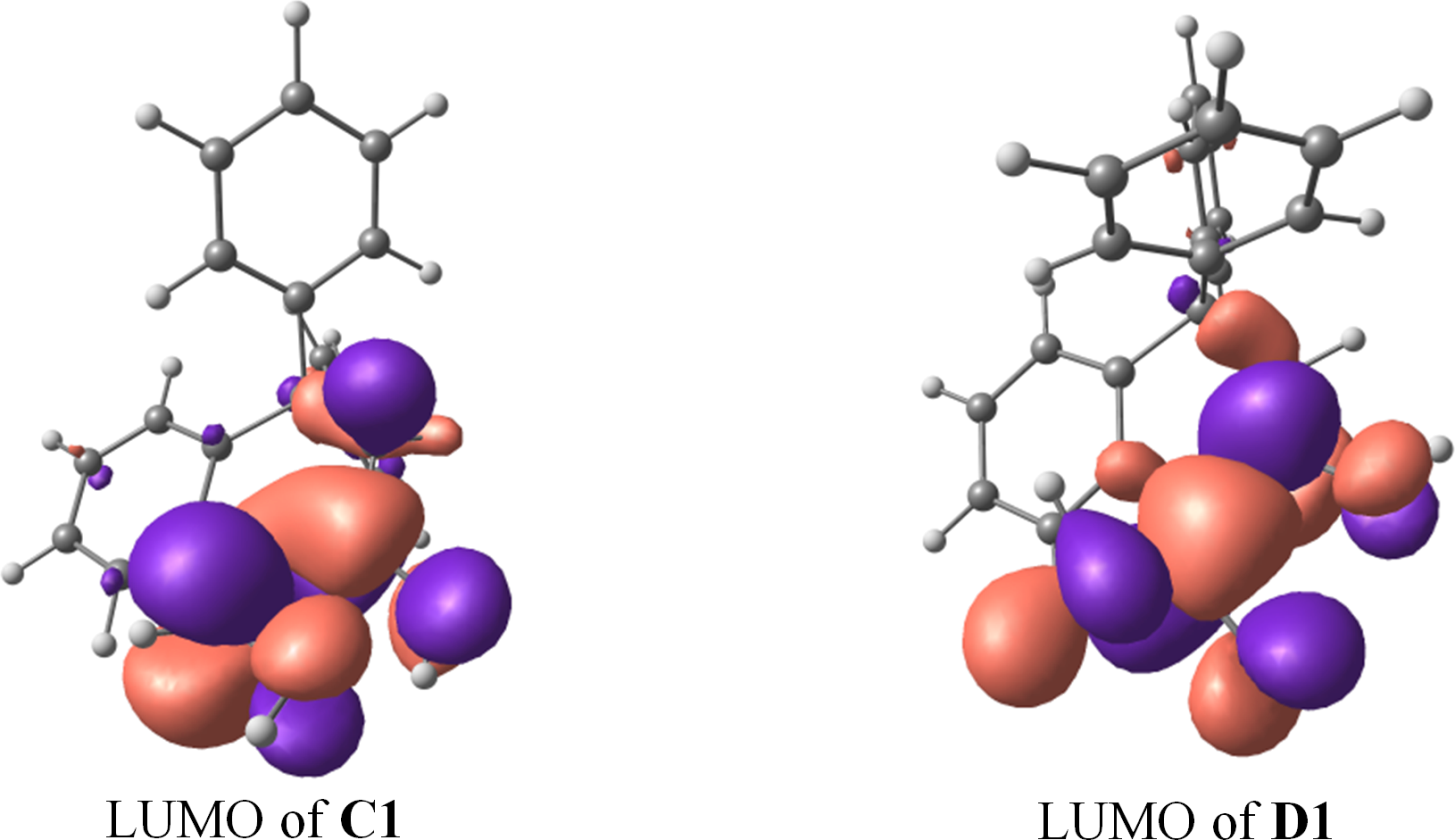
LUMO of species **C1** and **D1**.

Additionally a DFT calculation of dications **C1** and **D1** derived from **5a** and **6a**, respectively, was carried out to estimate the electrophilic properties of these species. Charge distribution, contribution of atomic orbital into LUMO and global electrophilicity indices ω [23–24] were calculated (Table 2, and Figure 2). The calculations showed that the C^α^ carbon of the protonated *N-*formyl and *N-*acyl groups in species **C1** and **D1**, respectively, have a rather large positive charge. Despite the large charge on C^2^ of the protonated carbonyl groups and its substantial contribution into LUMO (Figure 2), this carbon is not reactive, probably, due to steric reasons. A comparison of the electrophilicity indices ω of **C1** and **D1** (Table 2) revealed that the former is stronger electrophile. Also, perhaps, dication **D1** was not reactive due to the bigger spatial volume of the methyl substituent in the *N*-acyl group compared to hydrogen of the *N*-formyl one in **C1**, that hampered the reaction with arene molecule.

**Table 2:** Selected electronic characteristics (DFT calculations) of dications **C1** and **D1** derived from protonation of **5a** and **6a**, respectively.

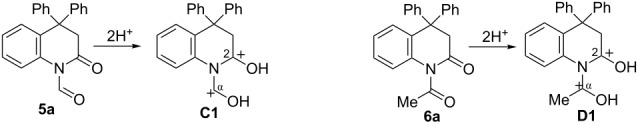									

cation	*E*_HOMO_, eV	*E*_LUMO_, eV	ω,^a^ eV	q(C^2^),^b^	q(C^a^),^b^	q(C^N^),^b^	*k*(C^2^)_LUMO_,^с^ %	*k*(C^a^)_LUMO_,^с^ %	*k*(C^N^)_LUMO_,^с^ %

**C1**	−7.11	−4.56	6.69	0.84	0.60	−0.44	14.8	11.5	1.1
**D1**	−7.22	−4.49	6.28	0.82	0.84	−0.50	24.9	11.9	3.1

^a^Global electrophilicity index ω = (*E*_HOMO_ + *E*_LUMO_) ^2^/8(*E*_LUMO_ − *E*_HOMO_). ^b^Natural charges. ^c^Contribution of atomic orbitals into the molecular orbital.

## Conclusion

A simple and effective approach to 4,4-diaryl-3,4-dihydroquinolin-2-(1*H*)-ones based on the reaction of 3-aryl-*N*(aryl)propiolamides with arenes under superelectrophilic activation in triflic acid has been developed. The synthetic potential of the obtained dihydroquinolinones has been demonstrated by their transformations into *N*-acyl, *N*-formyl, and *N*-diphenylmethyl derivatives.

## Supporting Information

File 1 Experimental procedures, characterization of compounds, ^1^H, ^13^C, ^19^F NMR spectra, and data on DFT calculations.
