# Supplementary material for: Reactions of N,3-diarylpropiolamides with arenes under superelectrophilic activation: synthesis of 4,4-diaryl-3,4-dihydroquinolin-2(1H)-ones and their derivatives
Source: Beilstein J Org Chem. 2016 May 11;12:950–6. doi: 10.3762/bjoc.12.93 (PMC4901988; doi:10.3762/bjoc.12.93)
Supplement: File 1 — Experimental procedures, characterization of compounds, 1H, 13C, 19F NMR spectra, and data on DFT calculations. [file Beilstein_J_Org_Chem-12-950-s001.pdf]

Supporting Information

for

Reactions of *N*,3-diarylpropiolamides with arenes under superelectrophilic activation: synthesis of 4,4-diaryl-3,4-dihydroquinolin-2(1*H*)-ones and their derivatives

Larisa Yu. Gurskaya[1,2], Diana S. Belyanskaya[1], Dmitry S. Ryabukhin[1,3], Denis I. Nilov[1], Irina A. Boyarskaya[1] and Aleksander V. Vasilyev*[1,3]

Address: [1]Department of Organic Chemistry, Institute of Chemistry, Saint Petersburg State University, Universitetskaya nab., 7/9, Saint Petersburg, 199034, Russia, [2]N.N. Vorozhtsov Novosibirsk Institute of Organic Chemistry, Siberian Branch of Russian Academy of Science, ul. Lavrentieva 9, Novosibirsk, 630090, Russia and [3]Department of Chemistry, Saint Petersburg State Forest Technical University, Institutsky per. 5, Saint Petersburg, 194021, Russia.

Email: Aleksander V. Vasilyev - aleksvasil@mail.ru

∗Corresponding author

[§]Tel./fax: +07-812-670-93-90

Experimental procedures, characterization of compounds, ^{1}H, ^{13}C, ^{19}F NMR spectra, and data on DFT calculations

Contents

1. Experimental section……………………………………………………………………….S2

2. ^{1}H, ^{13}C, ^{19}F NMR spectra of compounds……………………………………………….…S11

3. Details of DFT calculations………………………………………………………………….S62

4. References…………………………………………………………………………………...S72

1. Experimental section

The NMR spectra of solutions of compounds in $CDCl_3$, $(CD_3)_2CO$ or DMSO-d_6 were recorded on a Bruker-400 spectrometer at 25 °C (at 400, 100, and 376 MHz for ^1H, ^{13}C, and ^{19}F NMR spectra, respectively). The residual proton-solvent peak of $CDCl_3$ (δ 7.26 ppm), $(CD_3)_2CO$ (δ 2.05 ppm) for ^1H NMR spectra, the carbon signal of $CDCl_3$ (δ 77.0 ppm), $(CD_3)_2CO$ (δ 29.84 ppm), and DMSO-d_6 (δ 39.52 ppm) for ^{13}C NMR spectra, and the signal of $CFCl_3$ (δ 0.0 ppm) for ^{19}F NMR spectra were used as references. HRMS was carried out at instruments Bruker maXis HRMS-ESI-QTOF. The preparative reactions were monitored by thin-layer chromatography carried out on silica gel plates (Alugram SIL G/UV-254), using UV light for detection. Preparative column chromatography was performed on silica gel 60 Merck with hexanes–ethyl acetate mixture elution.

X-ray analysis. Similarly as described in [S1] suitable crystals were selected and studied on the diffractometer for X-ray analysis. The crystals were kept at 100(2) K during data collection. The structure was solved using Olex2 [S2] with the ShelXS [S3] structure solution program by means of Direct Methods and refined with the ShelXL refinement package using Least Squares minimization. CCDC 1456371 – for (**2f**) contains the supplementary crystallographic data, which can be obtained free of charge at www.ccdc.cam.ac.uk/conts/retrieving.htmL or from the Cambridge Crystallographic Data Centre, 12 Union Road, Cambridge CB2 1EZ, UK; Fax: (internat.)+44-1223-336-033; E-mail: deposit@ccdc.cam.ac.uk.

DFT calculations. Similarly as described in [S1] all computations were carried out at the DFT/HF hybrid level of theory using Becke's three-parameter hybrid exchange functional in combination with the gradient-corrected correlation functional of Lee, Yang, and Parr (B3LYP) by using GAUSSIAN 2003 program packages [S4]. The geometry optimizations were performed using the 6-311+G(2d,2p) basis set (standard 6-311 basis set added with polarization (d,p) and diffuse functions). Optimizations were performed on all degrees of freedom and solvent-phase optimized structures were verified as true minima with no imaginary frequencies. The Hessian matrix was calculated analytically for the optimized structures in order to prove the location of correct minima and to estimate the thermodynamic parameters. Solvent-phase calculations used the Polarizable Continuum Model (PCM) with water as a solvent.

Synthesis and properties of starting **3-aryl-*N*-(aryl)propiolamides 1a–u** were given in our previous works [S5,S6].

General procedure for the synthesis of 4,4-diaryl 3,4-dihydroquinolin-2(1*H*)-ones 2a–x from 3-aryl-*N*-(aryl)propiolamides 1a–u and arenes in TfOH (Table 1).

Similarly as previously described in [S4,S7] a mixture of amide **1** (0.2 mmol), arene (benzene, chlorobenzene or 1,2-dichlorobenzene, 1 mL), and TfOH (1 mL) was stirred at room temperature

for 0.5 h (or other time as indicated in Table 1). The mixture was poured into water (30 mL), and extracted with $CHCl_3$ (3×30 mL). The extracts were combined, washed with water, a saturated aqueous solution of $NaHCO_3$, water again. After drying over Na_2SO_4, the solvent was distilled off under reduced pressure, and the residue was subjected to chromatographic separation on silica gel using hexanes–ethyl acetate as an eluent.

Analogously the reactions were carried out for amide **1** (0.2 mmol), benzene (0.5 mL) in mixture of TfOH (2.42 mL) and SbF_5 (0.85 mL), having Hammet acidity function H_0 −18 (Table 1), or amide **1** (0.2 mmol) under the action of AlX_3 (X = Cl, Br, 1 mmol) in benzene (5 mL) (Table 1). Reaction mixtures were worked-up as described above.

Yields of the obtained dihydroquinolinones **2a–x** are presented in Table 1.

General procedure for the synthesis of 1-formyl-4,4-diaryl 3,4-dihydroquinolin-2(1*H*)-ones 5a–d from dihydroquinolinones 2a,d,e,h (Scheme 2).

A solution of $POCl_3$ (19.6 g, 0.27 mmol) and DMF (35.4 g, 0.23 mmol) in $CHCl_3$ (0.5 mL) was stirred at room temperature for 1 h. Then compound **2** (0.1 mmol) was added to the solution and the reaction mixture was refluxed for 2 h. Then $CHCl_3$ was distilled off under reduced pressure, and a saturated aqueous solution of $NaHCO_3$ was added to the residue. The reaction product was extracted with CH_2Cl_2 (3 × 10 mL). The extracts were combined, washed with water, and dried over Na_2SO_4. Then the solvent was distilled off under reduced pressure, and the residue was subjected to chromatographic separation on silica gel using hexanes–ethyl acetate as an eluent.

General procedure for the synthesis of 1-acyl-4,4-diaryl 3,4-dihydroquinolin-2(1*H*)-ones 6a–e from dihydroquinolinones 2a,e–h (Scheme 2).

A mixture of compound **2** (0.2 mmol) and Ac_2O (1.5 mL) was refluxed for 6 h. Then Ac_2O was distilled off under reduced pressure, and saturated aqueous solution of $NaHCO_3$ was added to the residue. The reaction product was extracted with CH_2Cl_2 (3 × 10 mL). The extracts were combined, washed with water, and dried over Na_2SO_4. Then the solvent was distilled off under reduced pressure, and the residue was subjected to chromatographic separation on silica gel using hexanes–ethyl acetate as an eluent.

General procedure for the synthesis of 1-diphenylmethyl-4,4-diaryl 3,4-dihydroquinolin-2(1*H*)-ones 7a–c from 1-formyl dihydroquinolinones 5a,b,d (Scheme 3).

A mixture of compound **5** (0.1 mmol), benzene (0.5 mL), and TfOH (0.5 mL) was stirred at room temperature for 18 h. The mixture was poured into water (30 mL), and extracted with $CHCl_3$ (3 × 30 mL). The extracts were combined, washed with water, a saturated aqueous solution of $NaHCO_3$, water again, and dried over Na_2SO_4, the solvent was distilled off under reduced pressure,

and the residue was subjected to chromatographic separation on silica gel using hexanes–ethyl acetate as an eluent.

Properties of the following quinolinone derivatives were given in our previous works: 4,4diphenyl-3,4-dihydroquinolin-2(1*H*)-one (**2a**) [S8], 6-chloro-4,4-diphenyl-3,4-dihydroquinolin-2(1*H*)-one (**2h**) [S5], 4-(4-methylphenyl)-4-phenyl-3,4-dihydroquinolin-2-(1*H*)-one (**2p**) [S5], 4-(4-fluorophenyl)-4-phenyl-3,4-dihydroquinolin-2-(1*H*)-one (**2r**) [S5], 4-phenylquinolin-2(*1H*)-one (**3a**) [S9], 4-phenyl 7,8-benzoquinolin-2(*1H*)-one (**3b**) [S5], 4-phenyl 5,6-benzoquinolin-2(*1H*)-one (**3c**) [S5]. Properties of *N*-(4-fluorophenyl)amide of 3,3-diphenylpropenoic acid (**4a**) and *N*-(4-chlorophenyl)amide of 3,3-diphenylpropenoic acid (**4b)** correspond to literature data [S10].

8-Methyl-4,4-diphenyl-3,4-dihydroquinolin-2(1*H*)-one (2b). M. p. 198-200 °C. ^{1}H NMR (400 MHz, $CDCl_3$): δ = 2.26 (s, 3H, CH_3), 3.43 (s, 2H, CH_2), 6.72 (d, *J* = 7.6 Hz, $1H_{Ar}$), 6.93 (t, *J* = 7.6 Hz, $1H_{Ar}$), 7.08-7.14 (m, $5H_{Ar}$), 7.28-7.33 (m, $6H_{Ar}$), 7.50 (s, NH). ^{13}C NMR (100 MHz, $CDCl_3$): δ = 17.2 (CH_3), 44.4 (CH_2), 52.1, 122.6, 123.5, 127.2, 127.6, 128.4, 128.7, 129.8, 131.5, 135.4, 143.9, 169.8 (C=O). HRMS (ESI) calcd. for $C_{22}H_{20}NO_2$ 314.1539 [M+H]; found: 314.1539.

7-Methyl-4,4-diphenyl-3,4-dihydroquinolin-2(1*H*)-one (2c). M. p. 227-229 °C. ^{1}H NMR (400 MHz, $CDCl_3$): δ = 2.33 (s, 3H, CH_3), 3.38 (s, 2H, CH_2), 6.63 (br.s, $1H_{Ar}$), 6.71 (d, *J* = 7.8 Hz, $1H_{Ar}$), 6.80 (d, *J* = 7.8 Hz, $1H_{Ar}$), 7.06-7.08 (m, $4H_{Ar}$), 7.23-7.31 (m, $6H_{Ar}$), 8.70 (br.s, 1NH). ^{13}C NMR (100 MHz, $CDCl_3$): δ = 21.1 (CH_3), 44.8 (CH_2), 51.7, 116.9, 123.9, 127.1, 128.4, 128.5, 128.7, 129.4, 137.0, 138.4, 144.0, 170.4 (C=O). HRMS (ESI) calcd. for $C_{22}H_{20}NO_2$ 314.1539 [M+H]; found: 314.1539.

6-Methyl-4,4-diphenyl-3,4-dihydroquinolin-2(1*H*)-one (2d). M. p. 232-234 °C. ^{1}H NMR (400 MHz, $CDCl_3$): δ = 2.25 (s, 3H, CH_3), 3.39 (s, 2H, CH_2), 6.66 (br.s, $1H_{Ar}$), 6.71 (d, *J* = 7.9 Hz, $1H_{Ar}$), 7.05-7.10 (m, $5H_{Ar}$), 7.26-7.34 (m, $6H_{Ar}$), 7.77 (s, NH). ^{13}C NMR (100 MHz, $CDCl_3$): δ = 21.2 (CH_3), 44.8 (CH_2), 52.0, 116.1, 127.2, 128.5, 128.7, 128.8, 130.0, 131.3, 132.7, 134.6, 143.8, 169.8 (C=O). HRMS (ESI) calcd. for $C_{22}H_{20}NO_2Na$ 336.1359 [M+Na]; found: 336.1344.

8-Fluoro-4,4-diphenyl-3,4-dihydroquinolin-2(1*H*)-one (2e). M. p. 239-240 °C. ^{1}H NMR (400 MHz, $CDCl_3$): δ = 3.42 (s, 2H, CH_2), 6.67 (d, *J* = 7.8 Hz, $1H_{Ar}$), 6.94 (td, *J* = 8.0, 5.5 Hz, $1H_{Ar}$), 7.04-7.07 (m, $5H_{Ar}$), 7.26-7.34 (m, $6H_{Ar}$), 7.68 (s, 1H, NH). ^{13}C NMR (100 MHz, $CDCl_3$): δ = 44.5 (CH_2), 114.5 (d, *J* = 18 Hz), 122.7 (d, *J* = 7 Hz), 124.9, 127.4, 128.6, 133.6, 150.4 (d, *J* = 244 Hz), 168.7 (C=O). HRMS (ESI) calcd. for $C_{21}H_{17}FNO$ 318.1289 [M+H]; found: 318.1289.

7-Fluoro-4,4-diphenyl-3,4-dihydroquinolin-2(1*H*)-one (2f). M. p. 235-236 °C. ^{1}H NMR (400 MHz, $(CD_3)_2CO$): δ = 3.41 (s, 2H, CH_2), 6.74 (dd, *J* = 7.6, 1.6 Hz, $2H_{Ar}$), 6.80-6.83 (m, $1H_{Ar}$), 7.07-7.14 (m, $4H_{Ar}$), 7.31-7.35 (m, $6H_{Ar}$), 8.29 (s, 1H, NH). ^{13}C NMR (100 MHz, $(CD_3)_2CO$): δ =

52.1 (CH_2), 103.5 (d, J = 26 Hz), 108.9 (d, J = 22 Hz), 131.1, 131.4, 140.6, 140.7, 144.8, 163.0 (d, J = 243 Hz), 169.4 (C=O). ^{19}F NMR (376 MHz, ($CDCl_3$): δ = -114.39 m. HRMS (ESI) calcd. for $C_{21}H_{17}FNO$ 318.1289 [M+H]; found: 318.1289.

Single crystal of compound **2f**, $C_{21}H_{16}FNO$, was studied by X-ray analysis at 100(2) K; rhombic crystal with size 0.25 × 0.15 × 0.05 mm, a 6.96671(15) b 14.8509(4) c 29.7304(8) Å, α 90°, β 3075.96(13), γ 90°, V 3075.96(14) Å^3, Z 4, space group $P2ac2ab$, $d_{calcul.}$ 1.371 g/cm^3, μ 0.747 mm^{-1}, 2.97≤θ≤69.95°; 35862 reflection were measured.

6-Fluoro-4,4-diphenyl-3,4-dihydroquinolin-2(1*H*)-one (2g). M. p. 239-240 °C. ^{1}H NMR (400 MHz, $CDCl_3$): δ = 3.40 (s, 2H, CH_2), 6.56 (dd, J = 9.1, 2.4 Hz, $1H_{Ar}$), 6.68 (td, J = 8.4, 2.5 Hz, $1H_{Ar}$), 6.78 (dd, J = 8.6, 6.0 Hz, $1H_{Ar}$), 7.03-7.06 (m, $4H_{Ar}$), 7.24-7.32 (m, $6H_{Ar}$), 8.15 (s, NH). ^{13}C NMR (100 MHz, $CDCl_3$): δ = 44.6 (CH_2), 51.6, 103.6 (d, J = 26 Hz), 109.6 (d, J = 21 Hz), 127.22, 127.26, 127.4, 128.6, 128.63, 131.0 (d, J = 9 Hz), 138.5 (d, J = 10.5 Hz), 143.5, 162.5 (d, J = 246 Hz), 170.2 (C=O). ^{19}F NMR (376 MHz, $CDCl_3$): δ = -113.68- -113.61 (m). HRMS (ESI) calcd. for $C_{21}H_{17}FNO$ 318.1289 [M+H]; found: 318.1289.

7,8-Dimethyl-4,4-diphenyl-3,4-dihydroquinolin-2(1*H*)-one (2i). M. p. 204-206 °C. ^{1}H NMR (400 MHz, $CDCl_3$): δ = 2.13 (s, 3H, CH_3), 2.31 (s, 3H, CH_3), 3.38 (s, 2H, CH_2), 6.57 (d, J = 7.9 Hz, $1H_{Ar}$), 6.82 (d, J = 7.9 Hz, $1H_{Ar}$), 7.06-7.08 (m, $4H_{Ar}$), 7.23-7.31 (m, $6H_{Ar}$), 7.50 (s, NH). ^{13}C NMR (100 MHz, $CDCl_3$): δ = 12.9 (CH_3), 20.5 (CH_3), 44.4 (CH_2), 52.0, 122.0, 124.4, 126.8, 127.1, 128.4, 128.8, 129.4, 135.3, 136.6, 144.0, 170.0 (C=O). HRMS (ESI) calcd. for $C_{23}H_{22}NO$ 328.1696 [M+H]; found: 328.1689.

6,8-Dimethyl-4,4-diphenyl-3,4-dihydroquinolin-2(1*H*)-one (2j). M. p. 214-215 °C. ^{1}H NMR (400 MHz, $CDCl_3$): δ = 1.50 (s, 3H, CH_3), 2.21 (s, 3H, CH_3), 3.34 (s, 2H, CH_2), 6.76 (d, J = 7.8 Hz, $1H_{Ar}$), 7.04 (d, J = 7.8 Hz, $1H_{Ar}$), 7.13-7.15 (m, $4H_{Ar}$), 7.19 (s, NH), 7.26-7.31 (m, $6H_{Ar}$). ^{13}C NMR (100 MHz, $CDCl_3$): δ = 17.6 (CH_3), 23.2 (CH_3), 47.5 (CH_2), 53.2, 121.7, 127.2, 127.3, 128.5, 128.7, 129.7, 130.0, 136.3, 136.8, 143.6, 168.6 (C=O). HRMS (ESI) calcd. for $C_{23}H_{21}NONa$ 350.1515 [M+Na]; found: 350.1520.

6,7-Dimethyl-4,4-diphenyl-3,4-dihydroquinolin-2(1*H*)-one (2k). M. p. 226-228 °C. ^{1}H NMR (400 MHz, $CDCl_3$): δ = 2.13 (s, 3H, CH_3), 2.23 (s, 3H, CH_3), 3.35 (s, 2H, CH_2), 6.58 (s, $1H_{Ar}$), 6.59 (s, $1H_{Ar}$), 7.06-7.08 (m, $4H_{Ar}$), 7.23-7.31 (m, $6H_{Ar}$), 7.97 (s, NH). ^{13}C NMR (100 MHz, $CDCl_3$): δ = 19.5 (CH_3), 19.6 (CH_3), 45.0 (CH_2), 51.7, 117.5, 127.1, 128.4, 128.6, 128.7, 130.4, 131.3, 134.8, 136.8, 144.1, 170.2 (C=O). HRMS (ESI) calcd. for $C_{23}H_{21}NONa$ 350.1515 [M+Na]; found: 350.1522.

8-Methoxy-4,4-diphenyl-3,4-dihydroquinolin-2(1*H*)-one (2l). M. p. 171-172 °C. ^{1}H NMR (400 MHz, $CDCl_3$): δ = 3.37 (s, 2H, CH_2), 3.85 (s, 3H, OCH_3), 6.66 (d, *J* = 6.5 Hz, $1H_{Ar}$), 6.82 (d, *J* = 8.2 Hz, $1H_{Ar}$), 6.93 (t, *J* = 8.0 Hz, $1H_{Ar}$), 7.06-7.08 (m, $4H_{Ar}$), 7.22-7.30 (m, $6H_{Ar}$) 7.75 (s, NH). ^{13}C NMR (100 MHz, $CDCl_3$): δ = 44.7 (CH_2), 52.2, 56.0 (OCH_3), 109.6, 121.5, 122.5, 126.6, 127.1, 128.4, 128.7, 131.7, 143.8, 146.4, 168.9 (C=O). HRMS (ESI) calcd. for $C_{22}H_{19}NO_2Na$ 352.1308 [M+Na]; found: 352.1312.

7-Methoxy-4,4-diphenyl-3,4-dihydroquinolin-2(1*H*)-one (2m). M. p. 246-248 °C. ^{1}H NMR (400 MHz, $CDCl_3$): δ = 3.38 (s, 2H, CH_2), 3.80 (s, 3H, OCH_3), 6.34 (d, *J* = 2.1 Hz, $1H_{Ar}$), 6.53 (dd, *J* = 8.6, 2.5 Hz, $1H_{Ar}$), 6.73 (d, *J* = 8.6 Hz, $1H_{Ar}$), 7.05-7.08 (m, $4H_{Ar}$), 7.23-7.31 (m, $6H_{Ar}$) 7.68 (s, NH). ^{13}C NMR (100 MHz, $CDCl_3$): δ = 45.0 (CH_2), 51.4, 55.6 (OCH_3), 102.2, 108.1, 123.8, 127.1, 128.5, 128.7, 130.6, 138.0, 144.1, 159.7, 170.1 (C=O). HRMS (ESI) calcd. for $C_{22}H_{19}NO_2Na$ 352.1308 [M+Na]; found: 352.1309.

6-Methoxy-4,4-diphenyl-3,4-dihydroquinolin-2(1*H*)-one (2n). M. p. 235-237 °C. ^{1}H NMR (400 MHz, $CDCl_3$): δ = 3.35 (s, 2H, CH_2), 3.66 (s, 3H, OCH_3), 6.39 (d, *J* = 1.1 Hz, $1H_{Ar}$), 6.76 (m, $1H_{Ar}$), 7.05-7.07 (m, $4H_{Ar}$), 7.22-7.30 (m, $6H_{Ar}$), 8.24 (s, NH). ^{13}C NMR (100 MHz, $CDCl_3$): δ = 44.5 (CH_2), 52.1, 55.6 (OCH_3), 112.7, 116.1, 117.1, 127.2, 128.4, 128.5, 128.7, 130.8, 132.9, 143.6, 155.6, 170.0 (C=O). HRMS (ESI) calcd. for $C_{22}H_{19}NO_2Na$ 352.1308 [M+Na]; found: 352.1299.

7-Fluoro-6-methoxy-4,4-diphenyl-3,4-dihydroquinolin-2(1*H*)-one (2o). M. p. 242-243 °C. ^{1}H NMR (400 MHz, $CDCl_3$): δ = 3.36 (s, 2H, CH_2), 3.65 (s, 3H, CH_3), 6.43 (d, *J* = 8.8 Hz, $1H_{Ar}$), 6.61 (d, *J* = 11.2 Hz, $1H_{Ar}$), 7.05-7.06 (m, $4H_{Ar}$), 7.27-7.33 (m, $6H_{Ar}$), 7.83 (s, NH). ^{13}C NMR (100 MHz, DMSO-d*6*): δ = 43.5 (CH_2), 51.2, 56.4 (OCH_3), 104.2 (d, *J* = 22 Hz), 115.0, 126.6, 126.7, 127.0, 128.3, 131.9 (d, *J* = 9 Hz) , 132.0, 141.5 (d, *J* = 11 Hz), 143.8, 150.8 (d, *J* = 244 Hz), 168.6 (C=O). ^{19}F NMR (376 MHz, DMSO-d*6*): δ = -130.92- -130.85 (m). HRMS (ESI) calcd. for $C_{22}H_{18}NFO_2Na$ 370.1214 [M+Na]; found: 370.1207.

6-Fluoro-4-(4-methylphenyl)-4-phenyl-3,4-dihydroquinolin-2(1*H*)-one (2q). M. p. 263-265 °C. ^{1}H NMR (400 MHz, $CDCl_3$): δ = 2.33 (s, 3H, CH_3), 3.35 (2H, CH_2), 6.58 (dd, *J* = 9.5, 2.7 Hz, $1H_{Ar,}$), 6.76 (dd, *J* = 8.6, 3.1 Hz, $1H_{Ar}$), 6.92-6.94 (m, $4H_{Ar}$), 7.11 (m, $5H_{Ar}$), 8.18 (s, 1H, NH). ^{13}C NMR (100 MHz, $CDCl_3$): δ = 20.8 (CH_3), 43.9 (CH_2), 51.4, 114.7, 116.3 (d, *J* = 24 Hz), 116.9 (d, *J* = 8.5 Hz), 120.6, 120.9, 127.1, 127.4, 128.2, 128.3, 128.5, 129.0, 130.1, 133.4, 136.8, 139.64, 143.0, 162.5 (d, *J* = 246 Hz), 169.8 (C=O). ^{19}F NMR (376 MHz, $CDCl_3$): δ = -113.60 (m). HRMS (ESI) calcd. for $C_{22}H_{18}NFONa$ 354.1265 [M+Na]; found: 354.1269.

4-(4-Chlorophenyl)-6-fluoro-4-phenyl-3,4-dihydroquinolin-2(1*H*)-one (2s). M. p. 161-162 ºC. ^{1}H NMR (400 MHz, $CDCl_3$): δ = 3.35 (m, 2H, CH_2), 6.56 (dd, *J* = 9.4, 2.7 Hz, $1H_{Ar}$), 6.80 (dd, *J*

= 8.6, 4.8 Hz, $1H_{Ar}$), 6.99 (t, *J* = 5.8 Hz, $2H_{Ar}$), 7.04 (dd, *J* = 8.6, 4.8 Hz, $3H_{Ar}$), 7.30 (dd, *J* = 8.3, 5.9 Hz, $5H_{Ar}$), 8.29 (s, 1H, NH). ^{13}C NMR (100 MHz, $CDCl_3$): δ = 44.2 (CH_2), 51.6, 115.3 (d, *J* = 23 Hz), 116.3 (d, *J* = 24.5 Hz), 117.7 (d, *J* = 8 Hz), 121.6, 128.5, 128.6, 128.7, 128.8, 130.0, 133.4, 133.5, 141.7, 142.2, 158.8 (d, *J* = 243 Hz), 170.0 (C=O). ^{19}F NMR (376 MHz, $CDCl_3$): δ = -118.61 (m). HRMS (ESI) calcd. for $C_{21}H_{16}NClFO$ 352.0899 [M+H]; found: 352.0891.

4,4-Diphenyl-3,4-dihydro-benzo[*h*]quinolin-2(1*H*)-one (2t). M. p. 292-294 °C. ^{1}H NMR (400 MHz, $CDCl_3$): δ = 3.51 (s, 1H, CH_2), 7.03 (d, *J* = 8.6 Hz, $1H_{Ar}$), 7.10-7.12 (m, $4H_{Ar}$), 7.51-7.54 (m, $3H_{Ar}$), 7.57 (d, *J* = 8.8 Hz, $1H_{Ar}$), 7.80 (d, *J* = 8.2 Hz, $1H_{Ar}$), 7.86 (d, *J* = 8.8 Hz, $1H_{Ar}$), 8.28 (s, 1H, NH). ^{13}C NMR (100 MHz, $CDCl_3$): δ = 52.4 (CH_2), 119.8, 122.6, 122.9, 126.5, 126.8, 127.0, 127.2, 127.2, 128.6, 128.6, 128.7, 128.8, 129.1, 129.1, 132.0, 133.3, 143.9, 170.1 (C=O). HRMS (ESI) calcd. for $C_{25}H_{20}NO$ 350.1539 [M+H]; found: 350.1539.

4,4-Diphenyl-3,4-dihydro-benzo[*f*]quinolin-2(1*H*)-one (2u). M. p. 150-151 °C. ^{1}H NMR (400 MHz, $CDCl_3$): δ = 3.50 (s, 2H, CH_2), 7.09 (m, $2H_{Ar}$),7.11 (d, *J* = 1.9 Hz, $2H_{Ar}$), 7.26-7.28 (m, $4H_{Ar}$), 7.37 (d, *J* = 7.3 Hz, $2H_{Ar}$), 7.4 (d, *J* = 7.5 Hz, $2H_{Ar}$), 7.63 (d, *J* = 8.2 Hz, $2H_{Ar}$), 7.74 (d, *J* = 8.2 Hz, $2H_{Ar}$), 8.07 (m, 1H, NH). ^{13}C NMR (100 MHz, $CDCl_3$): δ = 52.4 (CH_2), 53.6, 119.8, 122.6, 122.9, 126.5, 126.9, 127.0, 127.2, 127.5, 128.5, 128.6, 128.7, 128.8, 129.2, 129.3, 132.0, 133.3, 143.3, 143.9, 170.1 (C=O). HRMS (ESI) calcd. for $C_{25}H_{20}NO$ 350.1539 [M+H]; found: 350.1539.

4-(4-Chlorophenyl)-4-phenyl-3,4-dihydroquinolin-2(1*H*)-one (2v). M.p. 211-212 °C. ^{1}H NMR (400 MHz, $CDCl_3$): δ = 3.34 (d, *J* = 15.8 Hz, 1H, CH_2), 3.41 (d, *J* = 15.8 Hz, 1H, CH_2), 6.83 (d, *J* = 7.9 Hz, $2H_{Ar}$), 7.00-7.03 (m, $3H_{Ar}$), 7.05-7.07 (m, $2H_{Ar}$), 7.24-7.34 (m, $6H_{Ar}$), 8.16 (s, 1H, NH). ^{13}C NMR (100 MHz, $CDCl_3$): δ = 44.6 (CH_2), 51.6, 116.4, 123.3, 127.4, 128.58, 128.62, 128.64, 128.67, 129.4, 130.1, 131.0, 133.2, 137.0, 142.3, 143.3, 169. 9 (C=O). HRMS (ESI) calcd. for $C_{21}H_{16}NClONa$ 356.0813 [M+Na]; found: 356.0800.

4-(3,4-Dichlorophenyl)-4-phenyl-3,4-dihydroquinolin-2(1*H*)-one (2x). M. p. 222-224 °C.

^{1}H NMR (400 MHz, $CDCl_3$): δ = 3.29 (d, *J* = 15.8 Hz, 1H, CH_2), 3.45 (d, *J* = 15.8 Hz, 1H, CH_2), 6.81 (d, *J* = 7.6 Hz, $1H_{Ar}$), 6.84 (d, *J* = 7.8 Hz, $1H_{Ar}$), 6.92 (dd, *J* = 8.5, 2.3 Hz, $1H_{Ar}$), 7.00-7.05 (m, $3H_{Ar}$), 7.11 (d, *J* = 2.2 Hz, $1H_{Ar}$), 7.26-7.33 (m, $4H_{Ar}$), 7.36 (d, *J* = 8.5 Hz, $1H_{Ar}$), 8.21 (s, NH). ^{13}C NMR (100 MHz, $CDCl_3$): δ = 44.5 (CH_2), 51.6, 116.5, 123.5, 127.7, 128.2, 128.5, 128.79, 128.85, 129.3, 130.3, 130.4, 130.8, 131.6, 132.8, 137.0, 142.6, 144.4, 169.5 (C=O). HRMS (ESI) calcd. for $C_{21}H_{15}NCl_2ONa$ 390.0423 [M+Na]; found: 390.0418.

1-Formyl-4,4-diphenyl-3,4-dihydroquinolin-2(1*H*)-one (5a). Yield 30 %, m. p. 175-175.5 °C. ^{1}H NMR (400 MHz, $CDCl_3$): δ = 3.59 (s, 2H, CH_2), 6.75 (dd, *J* = 7.8, 1.4 Hz, $1H_{Ar}$), 7.01-7.03 (m, $2H_{Ar}$), 7.17 (td, *J* = 7.7, 1.5 Hz, $1H_{Ar}$), 7.27-7.34 (m, $3H_{Ar}$), 7.38 (td, *J* = 7.9, 1.5 Hz, $1H_{Ar}$),

7.89 (dd, *J* = 8.2, 0.9 Hz, $1H_{Ar}$), 9.35 (s, 1H, CHO). ^{13}C NMR (100 MHz, $CDCl_3$): δ = 46.3 (CH_2), 51.2, 123.0, 126.4, 127.6, 127.8, 128.6, 128.7, 128.8, 133.3, 136.6, 142.6. 160.1 (CHO), 172.7 (C=O). HRMS (ESI) calcd. for $C_{22}H_{17}NO_2Na$ 350.1151 [M+Na]; found: 350.1158.

1-Formyl-6-methyl-4,4-diphenyl-3,4-dihydroquinolin-2(1*H*)-one (5b). Yield 39 %, m. p. 207-208 °C. ^{1}H NMR (400 MHz, $CDCl_3$): δ = 2.24 (s, 3H, CH_3), 3.56 (s, 2H, CH_2), 6.53 (d, *J* = 1.6 Hz, $1H_{Ar}$), 7.02-7.04 (m, $4H_{Ar}$), 7.18 (dd, *J* = 8.3, 1.4 Hz, $1H_{Ar}$), 7.27-7.34 (m, $6H_{Ar}$), 7.77 (d, *J* = 8.3 Hz, $1H_{Ar}$), 9.33 (s, 1H, CHO). ^{13}C NMR (100 MHz, $CDCl_3$): δ = 21.3 (CH_3), 46.5 (CH_2), 51.0, 122.9, 127.6, 128.3, 128.6, 128.7, 129.2, 130.8, 136.2, 136.4, 142.7, 160.1 (CH=O), 172.8 (C=O). HRMS (ESI) calcd. for $C_{23}H_{19}NO_2Na$ 364.1308 [M+Na]; found: 364.1312.

1-Formyl-8-fluoro-4,4-diphenyl-3,4-dihydroquinolin-2(1*H*)-one (5c). Yield 31 %, m. p. 162-163 °C. ^{1}H NMR (400 MHz, $CDCl_3$): δ = 3.57 (s, 2H, CH_2), 6.52-6.54 (m, $1H_{Ar}$), 6.95-7.10 (m, $4H_{Ar}$), 7.14-7.23 (m, $2H_{Ar}$), 7.29-7.33 (m, $7H_{Ar}$), 9.03 (d, *J* = 3.0 Hz, 1H, CHO). ^{13}C NMR (100 MHz, $CDCl_3$): δ = 46.7 (CH_2), 51.8 d (*J* = 1.5 Hz), 116.2 d (*J* = 21 Hz), 121.3 (*J* = 12 Hz), 123.9 (*J* = 3 Hz), 127.9, 128.0 d (*J* = 8 Hz), 128.5, 128.9, 140.9, 141.9, 155.3 d (*J* = 256 Hz), 157.7 (CH=O), 172.3 (C=O). ^{19}F NMR (376 MHz, $CDCl_3$): δ = -111.60 - -111.64 (m). HRMS (ESI) calcd. for $C_{22}H_{16}NFO_2Na$ 368.1057 [M+Na]; found: 368.1066.

6-Chloro-1-formyl-4,4-diphenyl-3,4-dihydroquinolin-2(1*H*)-one (5d). Yield 37 %, m. p. 156-157 °C. ^{1}H NMR (400 MHz, $CDCl_3$): δ = 3.57 (s, 2H, CH_2), 6.73 (d, *J* = 2.4 Hz, $1H_{Ar}$), 7.00-7.05 (m, $4H_{Ar}$), 7.30-7.37 (m, $7H_{Ar}$), 7.86 (d, *J* = 8.7 Hz, $1H_{Ar}$), 9.32 (s, 1H, CHO). ^{13}C NMR (100 MHz, $CDCl_3$): δ = 46.1 (CH_2), 51.0, 124.4, 128.0, 128.4, 128.6, 128.7, 129.0, 131.8, 132.1, 138.6, 141.8, 159.9 (CH=O), 172.0 (C=O). HRMS (ESI) calcd. for $C_{22}H_{16}NClO_2Na$ 384.0762 [M+Na]; found: 384.0767.

1-Acetyl-4,4-diphenyl-3,4-dihydroquinolin-2(1*H*)-one (6a). Yield 65%, m. p. 139–140 °C. ^{1}H NMR (400 MHz, $CDCl_3$): δ = 2.14 (s, 3H, CH_3), 3.56 (s, 2H, CH_2), 6.69 (dd, *J* = 7.8, 1.3 Hz, $1H_{Ar}$), 7.05-7.07 (m, $4H_{Ar}$), 7.28-7.35 (m, $7H_{Ar}$), 7.53 (dd, *J* = 8.1, 0.9 Hz, $1H_{Ar}$). ^{13}C NMR (100 MHz, $CDCl_3$): δ = 27.1 (CH_3), 48.3 (CH_2), 51.1, 125.4, 126.0, 127.2, 127.4, 127.9, 128.6, 128.7, 135.7, 138.2, 142.9, 171.6 (C=O), 172.6 (C=O). HRMS (ESI) calcd. for $C_{23}H_{19}NO_2Na$ 364.1308 [M+Na]; found: 364.1321.

1-Acetyl-8-fluoro-4,4-diphenyl-3,4-dihydroquinolin-2(1*H*)-one (6b). Yield 57%, m. p. 177–178 °C. ^{1}H NMR (400 MHz, $CDCl_3$): δ = 2.27 (s, 3H, CH_3), 3.56 (s, 2H, CH_2), 6.48–6.50 (m, $1H_{Ar}$), 7.04–7.09 (m, $4H_{Ar}$), 7.09-7.17 (m, $2H_{Ar}$), 7.28-7.34 (m, $6H_{Ar}$). ^{13}C NMR (100 MHz, $CDCl_3$): δ = 26.7 (CH_3), 48.5 (CH_2), 51.7, 51.7, 115.5 (d, *J* = 21 Hz), 123.4 (d, *J* = 3 Hz), 124.7 (d, *J* = 11 Hz), 127.3 d (*J* = 9 Hz), 127.6, 128.5, 128.8, 141.4, 142.3, 155.6 д (d, *J* = 254 Hz), 169.6

(C=O), 171.1 (C=O). ^{19}F NMR (376 MHz, $CDCl_3$): δ = -113.23 (dd, *J* = 9.8, 5.5 Hz). HRMS (ESI) calcd. for $C_{23}H_{18}NFO_2Na$ 382.1214 [M+Na]; found: 382.1209.

1-Acetyl-7-fluoro-4,4-diphenyl-3,4-dihydroquinolin-2(1*H*)-one (6c). Yield 69%, m. p. 111–112 °C. ^{1}H NMR (400 MHz, $CDCl_3$): δ = 2.13 (s, 3H, CH_3), 3.55 (s, 2H, CH_2), 6.83 (td, *J* = 8.3, 2.6 Hz, $1H_{Ar}$), 6.64 (dd, *J* = 8.6, 6.1 Hz, $1H_{Ar}$), 7.03-7.05 (m, $4H_{Ar}$), 7.28-7.36 (m, $7H_{Ar}$). ^{13}C NMR (100 MHz, $CDCl_3$): δ = 27.1 (CH_3), 48.1 (CH_2), 50.7, 112.6 (d, *J* = 21 Hz), 113.0 (d, *J* = 26 Hz), 127.6, 128.5, 128.8, 129.0 (d, *J* = 9 Hz), 133.9 (d, *J* = 3.2 Hz), 136.8 (d, *J* = 11 Hz), 142.7, 161.3 (d, *J* = 246 Hz), 171.6 (C=O), 172.2 (C=O). ^{19}F NMR (376 MHz, $CDCl_3$): δ = -113.70 (dt, *J* = 9.2, 6.5 Hz). HRMS (ESI) calcd. for $C_{23}H_{18}NFO_2Na$ 382.1214 [M+Na]; found: 382.1219.

1-Acetyl-6-fluoro-4,4-diphenyl-3,4-dihydroquinolin-2(1*H*)-one (6d). Yield 56%, m. p. 133–134 °C. ^{1}H NMR (400 MHz, $CDCl_3$): δ = 2.11 (s, 3H, CH_3), 3.56 (s, 2H, CH_2), 6.41 (dd, *J* = 9.3, 2.9 Hz, $1H_{Ar}$), 7.00-7.05 (m, 5H, H_{Ar}), 7.29-7.34 (m, $6H_{Ar}$), 7.53 (dd, *J* = 8.9, 5.1 Hz, $1H_{Ar}$). ^{13}C NMR (100 MHz, $CDCl_3$): δ = 26.9 (CH_3), 48.3 (CH_2), 51.1, 114.0 (d, *J* = 23 Hz), 115.0 (d, *J* = 25 Hz), 127.4 (d, *J* = 8 Hz), 127.7, 128.4, 128.9, 131.5 (d, *J* = 3 Hz), 140.9 (d, *J* = 7 Hz), 142.3, 160.3 (d, *J* = 247 Hz), 171.2 (C=O), 172.5 (C=O). ^{19}F NMR (376 MHz, $CDCl_3$): δ = -114.66 td (*J* = 8.6, 5.5 Hz). HRMS (ESI) calcd. for $C_{23}H_{18}NFO_2Na$ 382.1214 [M+Na]; found: 382.1225.

1-Acetyl-6-chloro-4,4-diphenyl-3,4-dihydroquinolin-2(1*H*)-one (6e). Yield 50%, m. p. 130–131 °C. ^{1}H NMR (400 MHz, $CDCl_3$): δ = 2.12 (s, 3H, CH_3), 3.55 (s, 2H, CH_2), 6.67 (d, *J* = 2.4 Hz, $1H_{Ar}$), 7.03-7.04 (m, $4H_{Ar}$), 7.29-7.35 (m, $7H_{Ar}$), 7.49 (d, *J* = 8.7 Hz, $1H_{Ar}$). ^{13}C NMR (100 MHz, $CDCl_3$): δ = 27.0 (CH_3), 48.2 (CH_2), 51.1, 126.9, 127.3, 127.8, 127.8, 128.4, 128.9, 131.7, 134.2, 140.3, 142.2, 171.3 (C=O), 172.1 (C=O). HRMS (ESI) calcd. for $C_{23}H_{18}NClO_2Na$ 398.0918 [M+Na]; found: 398.0906.

4,4-Diphenyl-1-(diphenylmethyl)-3,4-dihydroquinolin-2(1*H*)-one (7a). Yield 62%, m. p. 210–211 °C. ^{1}H NMR (400 MHz, $CDCl_3$): δ = 3.62 (s, 2H, CH_2), 6.78-6.80 (m, 1H, CH), 6.85-6.96 (m, $7H_{Ar}$), 7.09-7.20 (m, $11H_{Ar}$), 7.32-7.36 (m, $6H_{Ar}$). ^{13}C NMR (100 MHz, $CDCl_3$): δ = 45.3 (CH_2), 51.1, 60.9 (CH), 119.8, 122.8, 126.8, 127.1, 127.2, 128.2, 128.6, 128.7, 128.9, 129.0, 135.6, 138.1, 138.7, 143.4, 169.5 (C=O). HRMS (ESI) calcd. for $C_{34}H_{28}NO$ 466.2165 [M+H]; found: 466.2186.

6-Methyl-4,4-diphenyl-1-(diphenylmethyl)-3,4-dihydroquinolin-2(1*H*)-one (7b). Yield 80%, m. p. 225-227 °C. ^{1}H NMR (400 MHz, $CDCl_3$): δ = 2.13 (s, 3H, CH_3), 3.60 (s, 2H, CH_2), 6.59 (s, $1H_{Ar}$), 6.73 (dd, *J* = 8.5, 1.2 Hz, $1H_{Ar}$), 6.77 (d, *J* = 8.3 Hz, $1H_{Ar}$), 6.87-6.90 (m, $4H_{Ar}$), 7.09-7.17 (m, $11H_{Ar}$), 7.32-7.36 (m, $5H_{Ar}$). ^{13}C NMR (100 MHz, $CDCl_3$): δ = 21.0 (CH_3), 45.5 (CH_2), 51.1, 60.7 (CH), 119.6, 127.0, 127.1, 127.3, 128.2, 128.5, 128.7, 129.0, 129.4, 132.3, 135.4, 136.2, 138.1, 143.6, 169.4 (C=O). HRMS (ESI) calcd. for $C_{35}H_{29}NONa$ 502.2141 [M+Na]; found: 502.2164.

6-Chloro-4,4-diphenyl-1-(diphenylmethyl)-3,4-dihydroquinolin-2(1*H*)-one (7c). Yield 36%, m. p. 226-228 °C. ^{1}H NMR (400 MHz, $CDCl_3$): δ = 3.61 (s, 2H, CH_2), 6.76 (d, *J* = 2.4 Hz, $1H_{Ar}$), 6.79 (d, *J* = 8.8 Hz, $1H_{Ar}$), 6.84-6.86 (m, $4H_{Ar}$), 6.89 (dd, *J* = 8.8, 2.4 Hz, $1H_{Ar}$), 7.07-7.09 (m, $4H_{Ar}$), 7.14-7.21 (m, $7H_{Ar}$), 7.34-7.36 (m, $6H_{Ar}$). ^{13}C NMR (100 MHz, $CDCl_3$): δ = 45.0 (CH_2), 51.1, 60.5 (CH), 121.8, 126.8, 127.3, 127.5, 128.3, 128.6, 128.7, 128.8, 128.9, 137.1, 137.6, 142.6, 169.2 (C=O). HRMS (ESI) calcd. for $C_{34}H_{26}NClONa$ 522.1595 [M+Na]; found: 522.1616.

^{1}H, ^{13}C, ^{19}F NMR Spectra of compounds

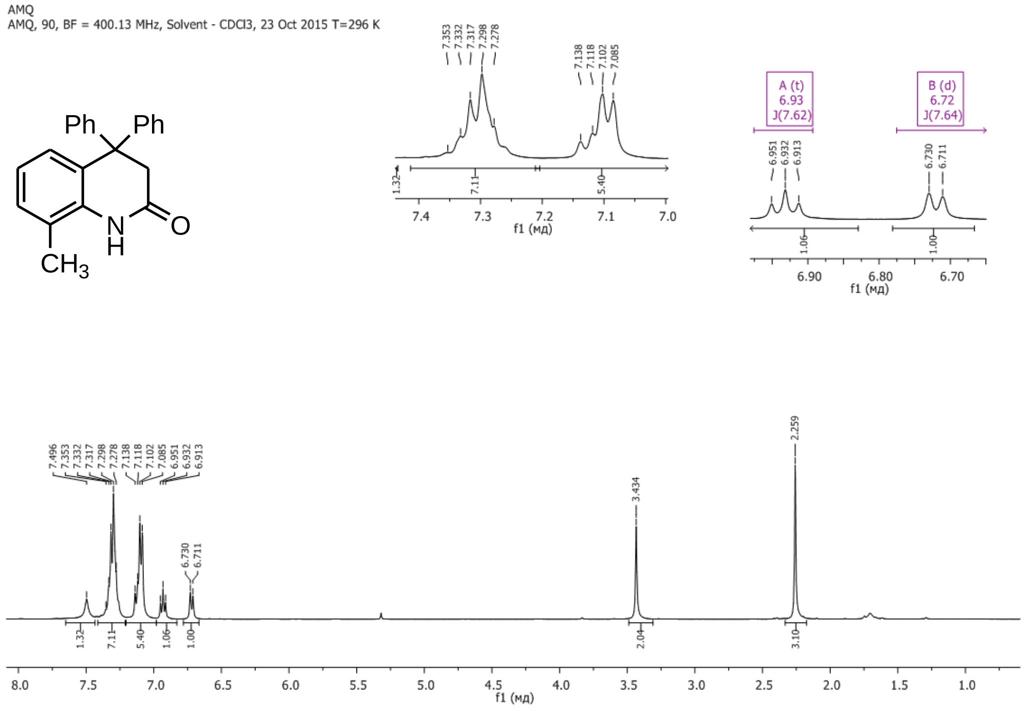

Figure S1. ^{1}H NMR spectrum of the compound **2b** (400 MHz, $CDCl_3$).

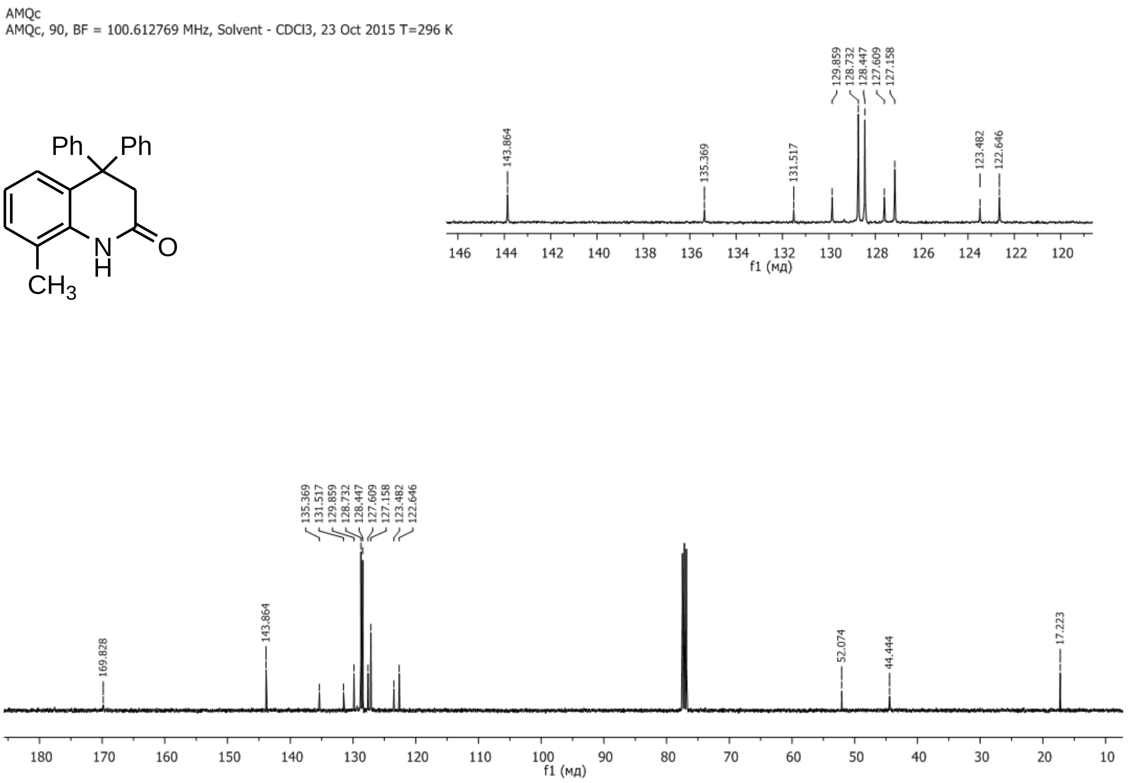

Figure S2. ^{13}C NMR spectrum of the compound **2b** (100 MHz, $CDCl_3$).

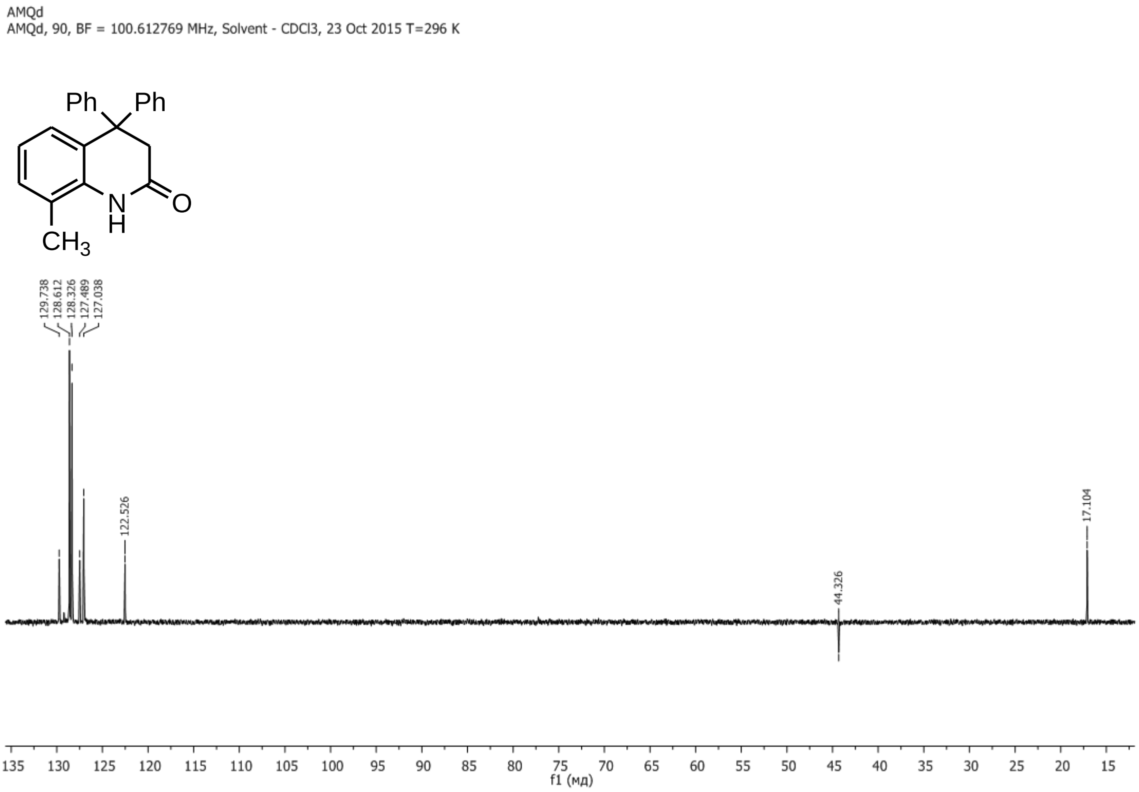

Figure S3. DEPT spectrum of the compound **2b** (100 MHz, $CDCl_3$).

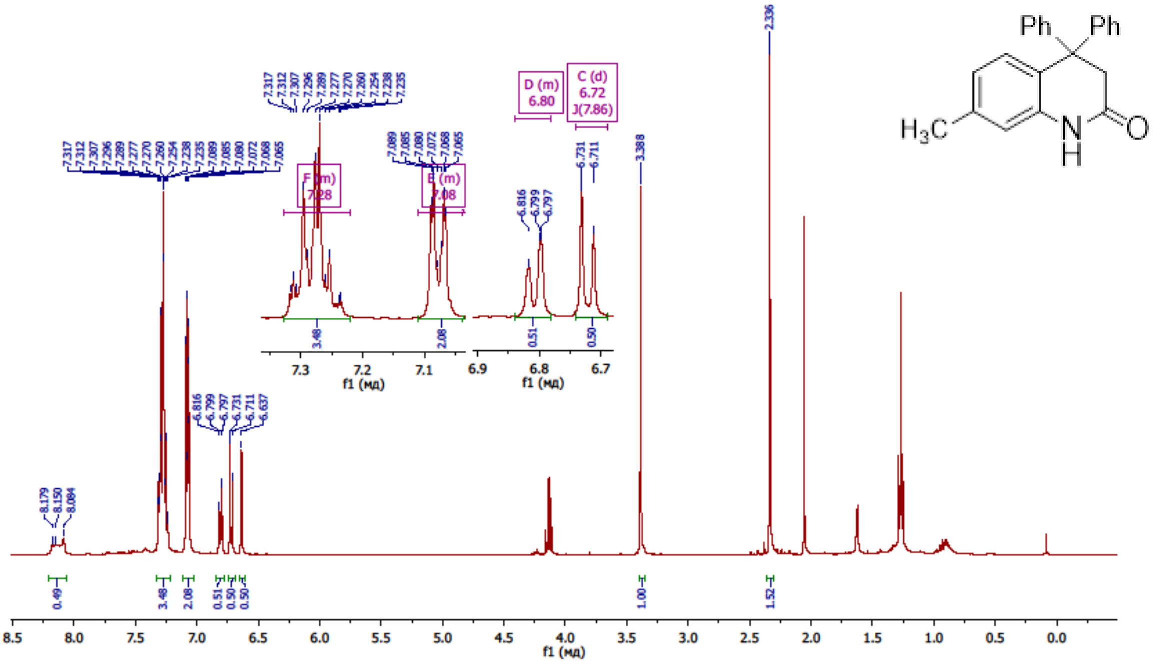

Figure S4. ^{1}H NMR spectrum of the compound **2c** (400 MHz, $CDCl_3$).

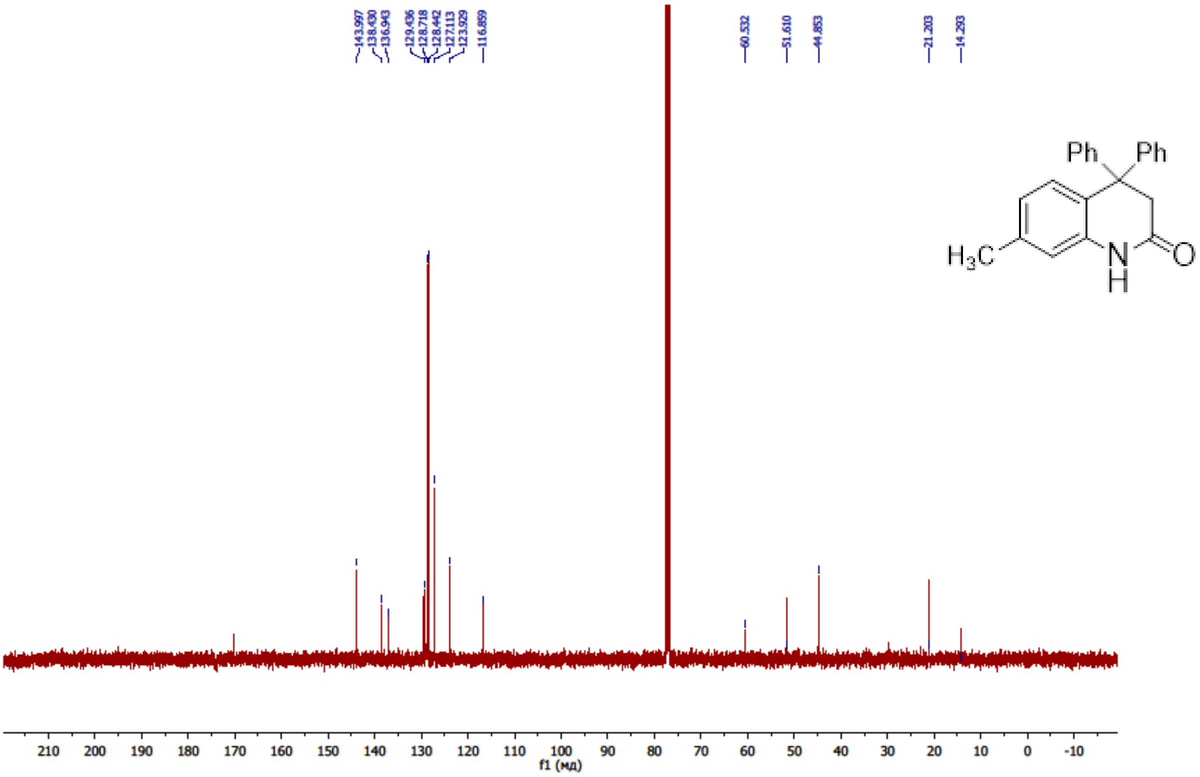

Figure S5. ^{13}C NMR spectrum of the compound **2c** (100 MHz, $CDCl_3$).

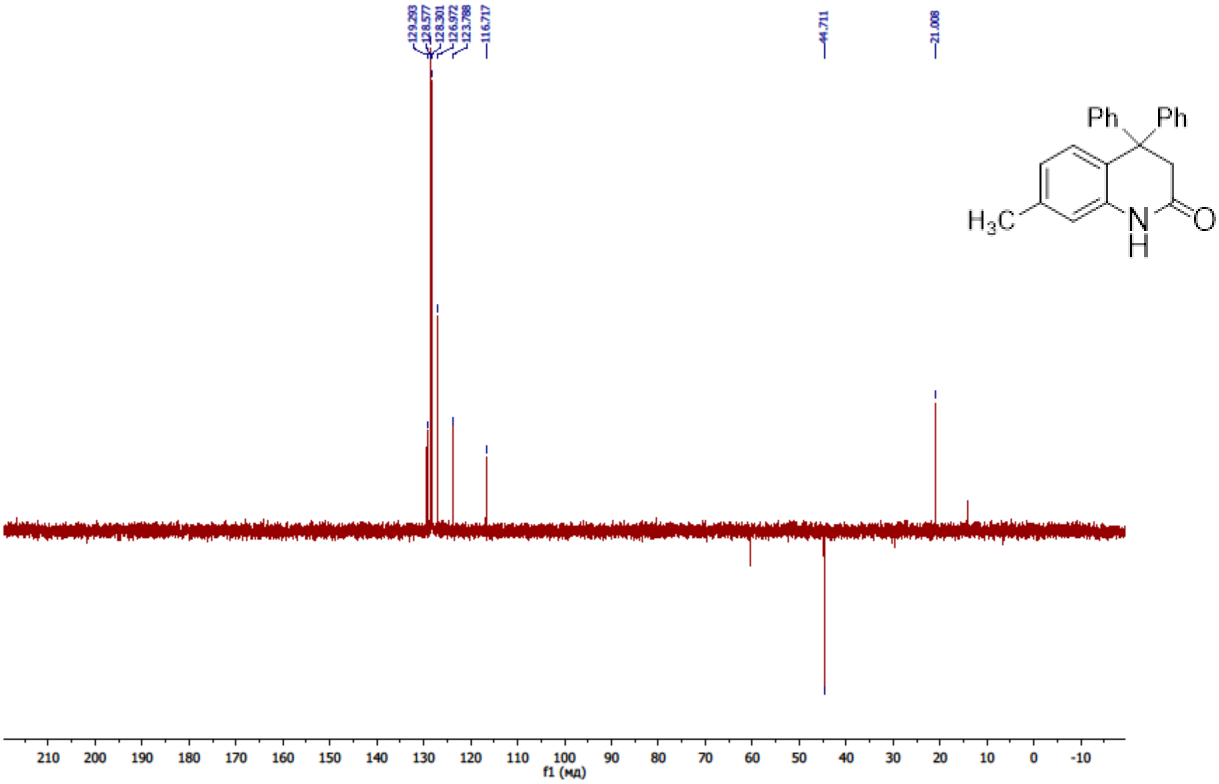

Figure S6. DEPT spectrum of the compound **2c** (100 MHz, $CDCl_3$).

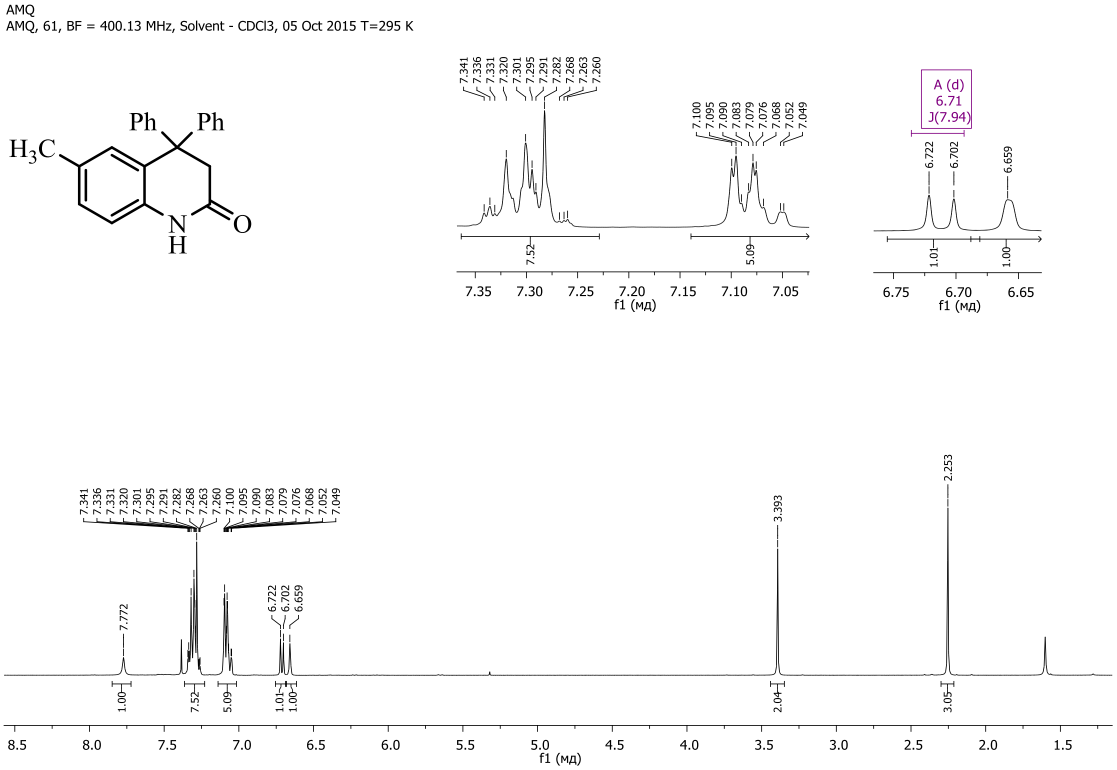

Figure S7. ^{1}H NMR spectrum of the compound **2d** (400 MHz, $CDCl_3$).

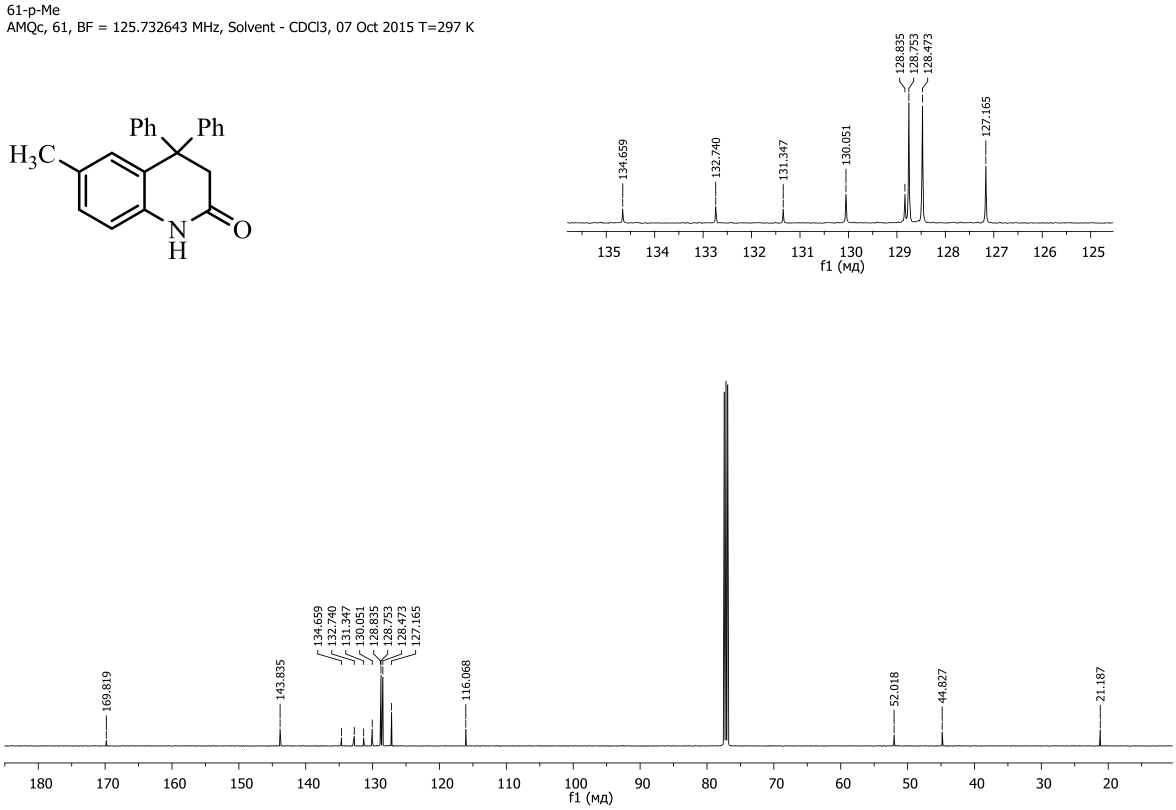

Figure S8. ^{13}C NMR spectrum of the compound **2d** (100 MHz, $CDCl_3$).

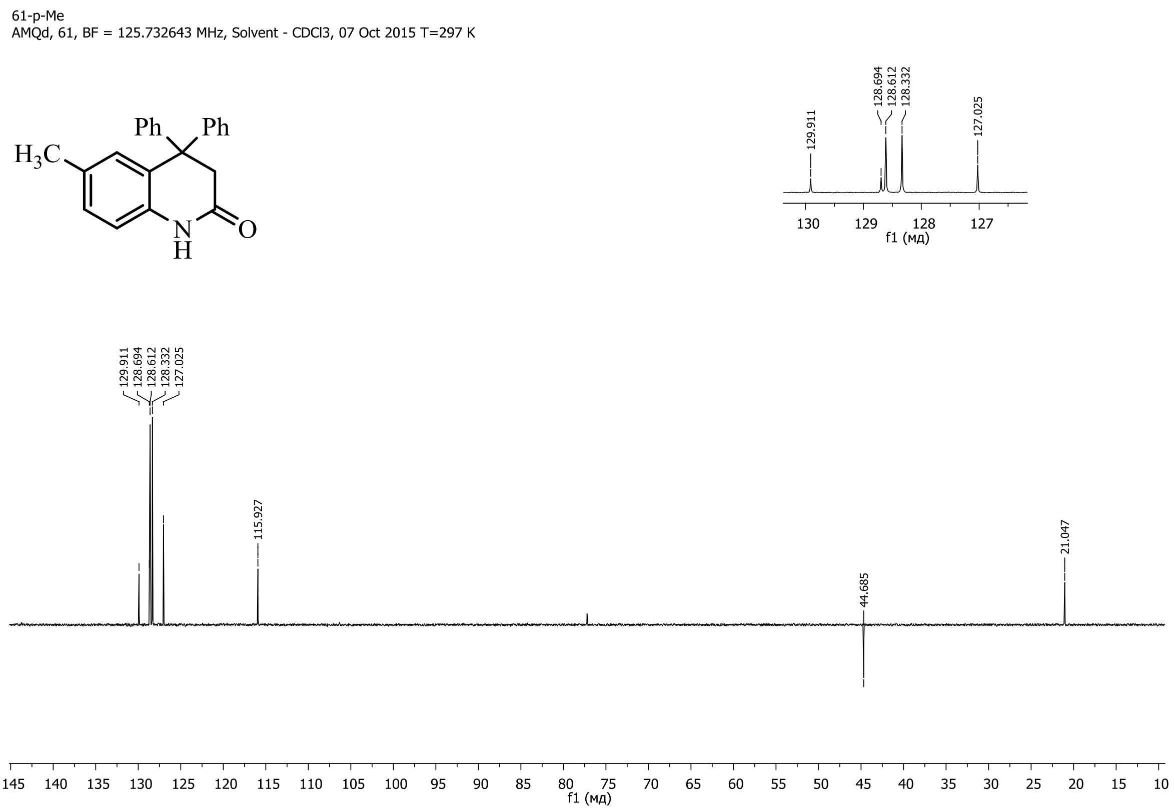

Figure S9. DEPT spectrum of the compound **2d** (100 MHz, $CDCl_3$).

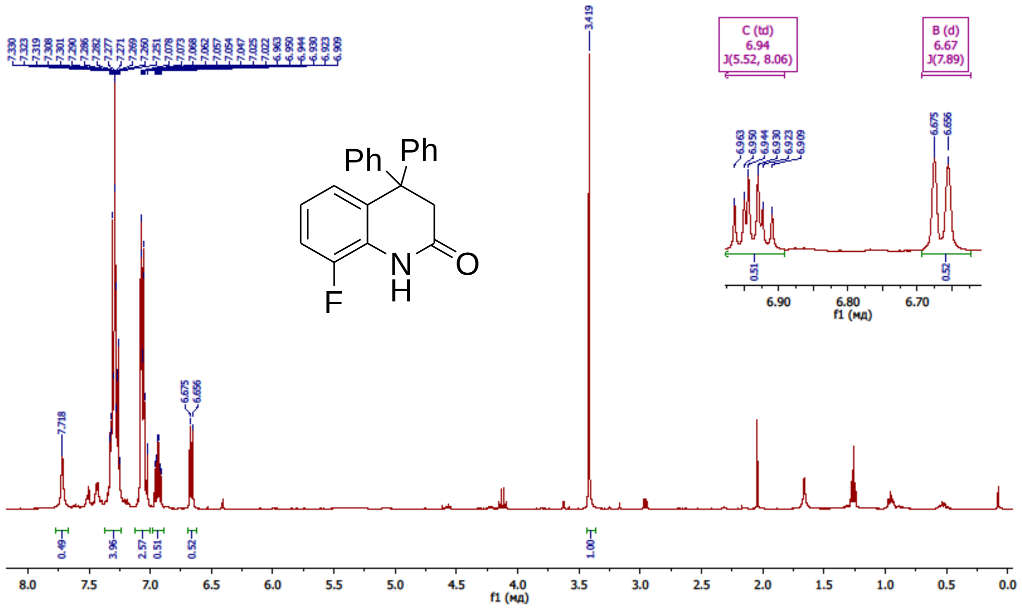

Figure S10. ^{1}H NMR spectrum of the compound **2e** (400 MHz, $CDCl_3$).

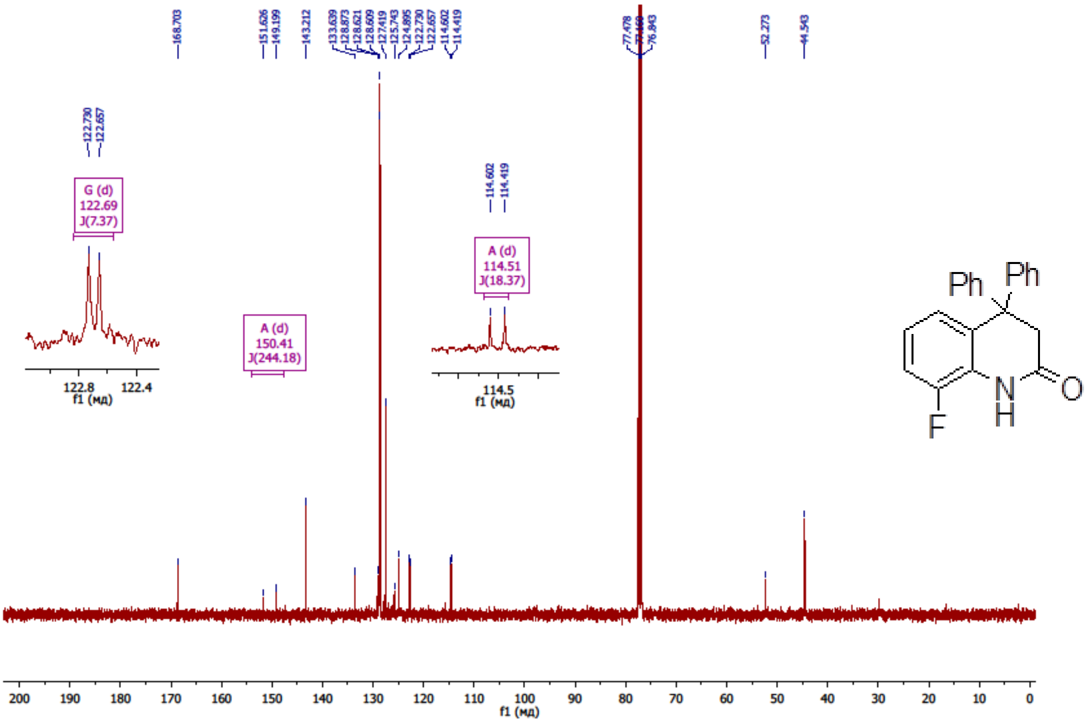

Figure S11. ^{13}C NMR spectrum of the compound **2e** (100 MHz, $CDCl_3$).

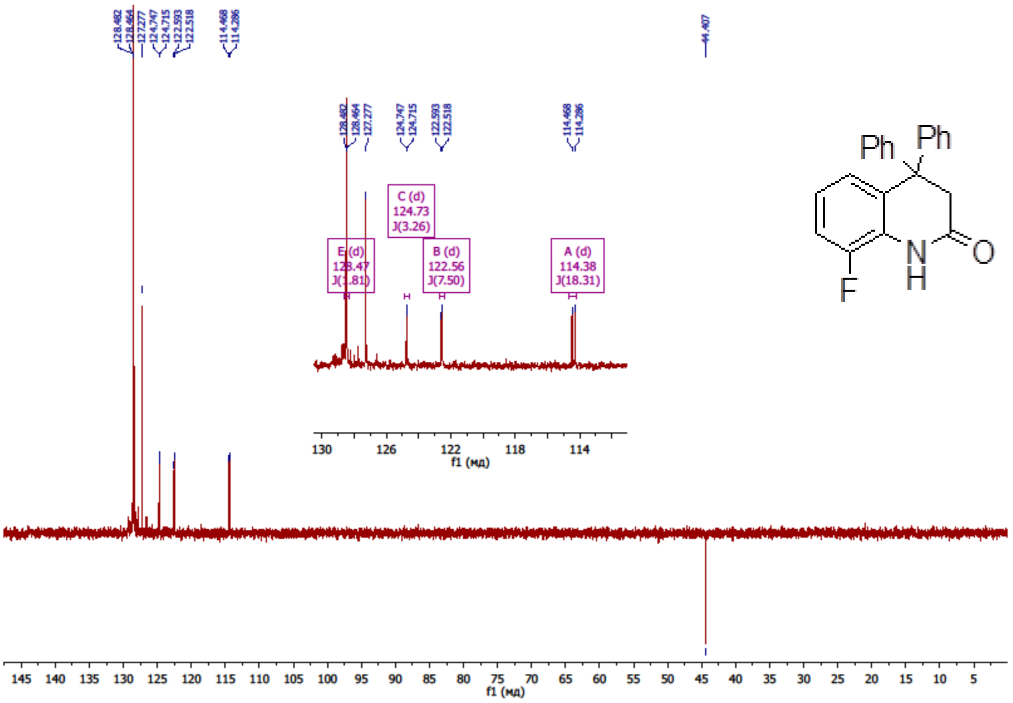

Figure S12. DEPT spectrum of the compound **2e** (100 MHz, $CDCl_3$).

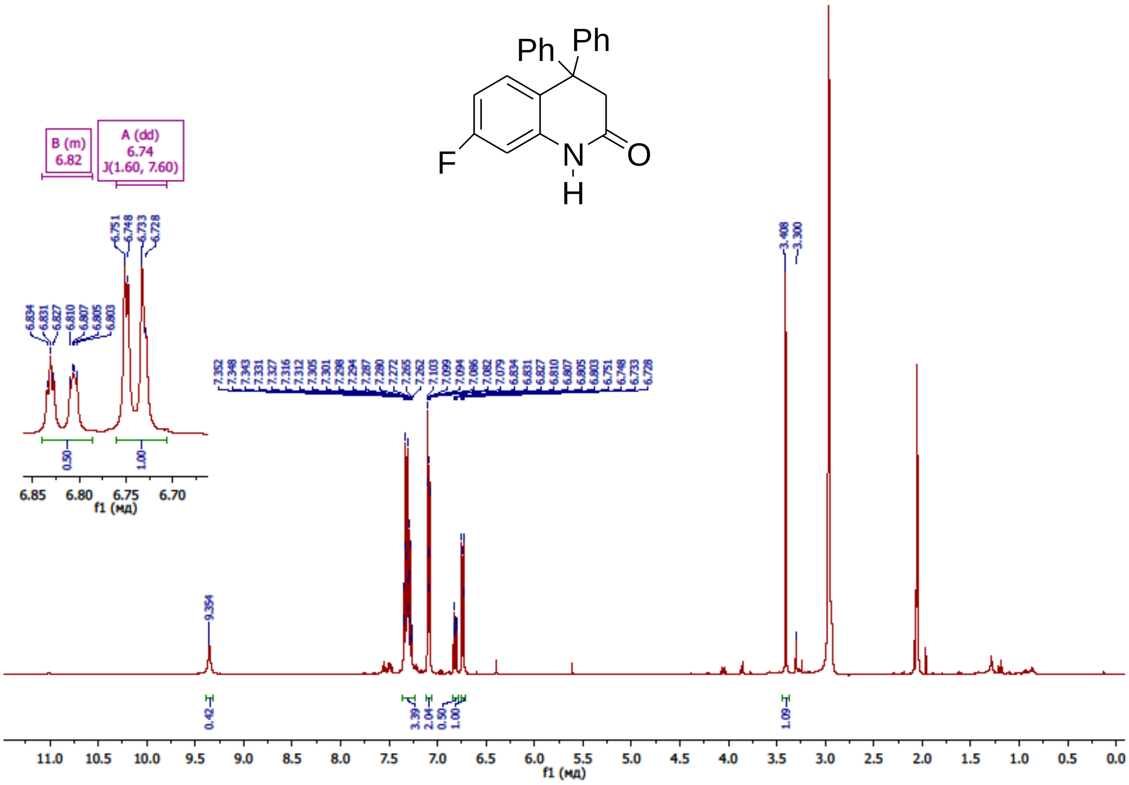

Figure S13. ^{1}H NMR spectrum of the compound **2f** (400 MHz, $(CD_3)_2CO$).

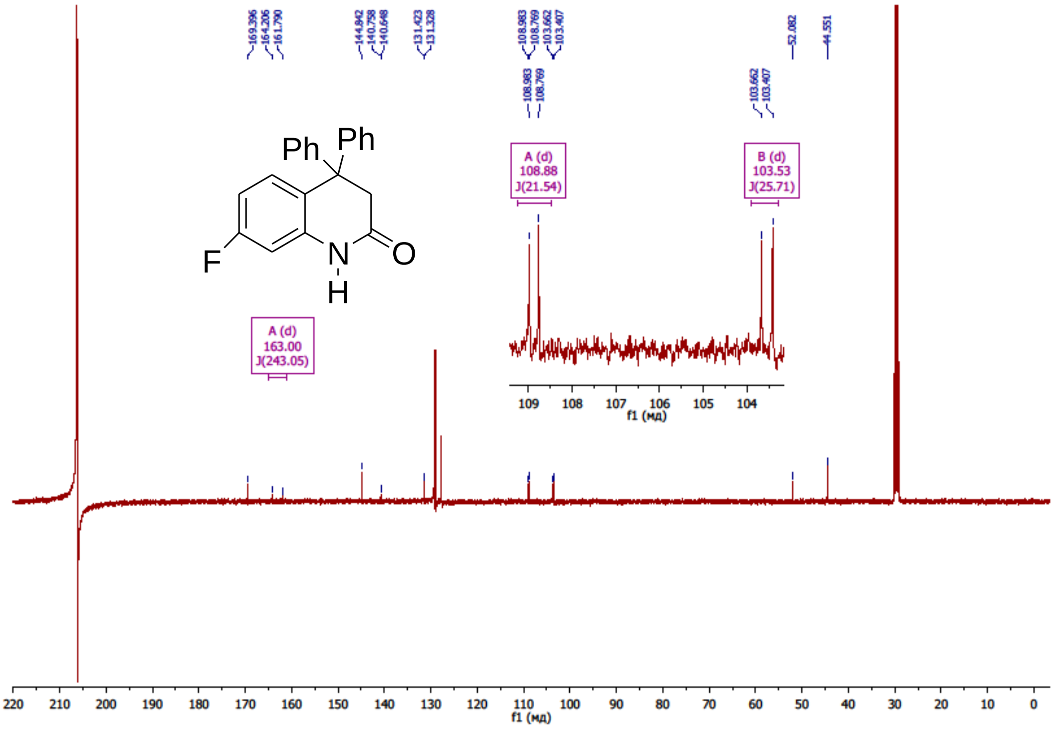

Figure S14. ^{13}C NMR spectrum of the compound **2f** (100 MHz, $(CD_3)_2CO$).

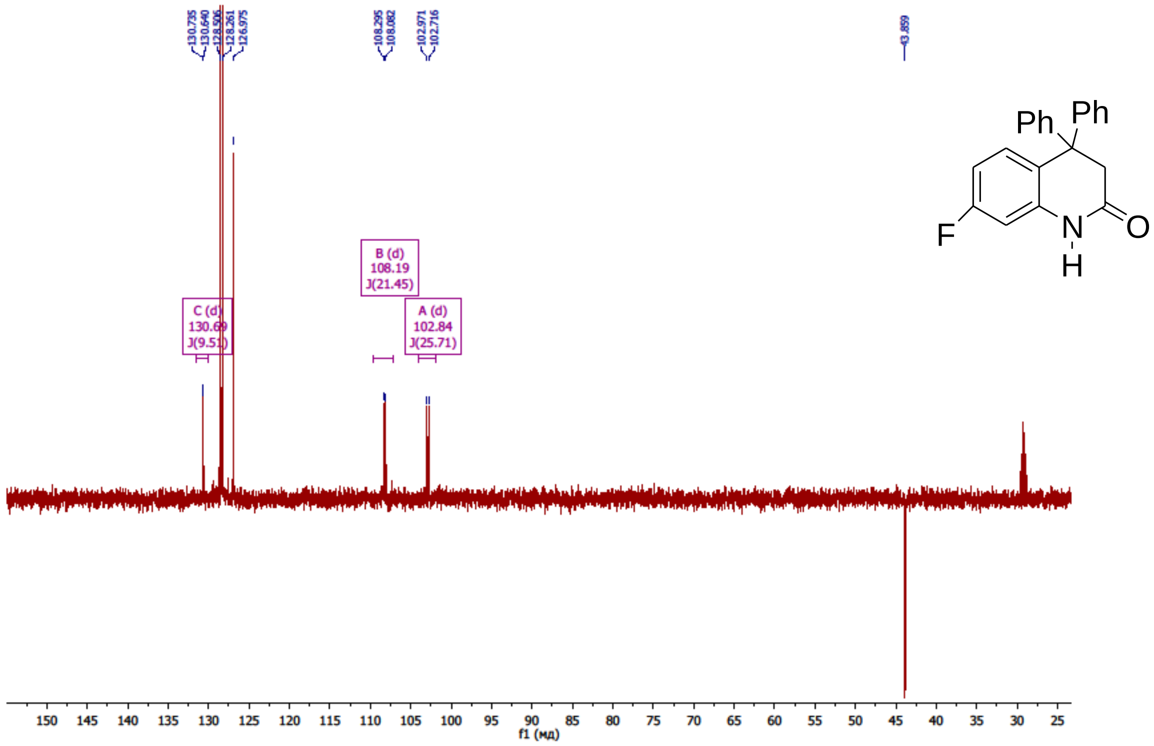

Figure S15. DEPT spectrum of the compound **2f** (100 MHz, $(CD_3)_2CO$).

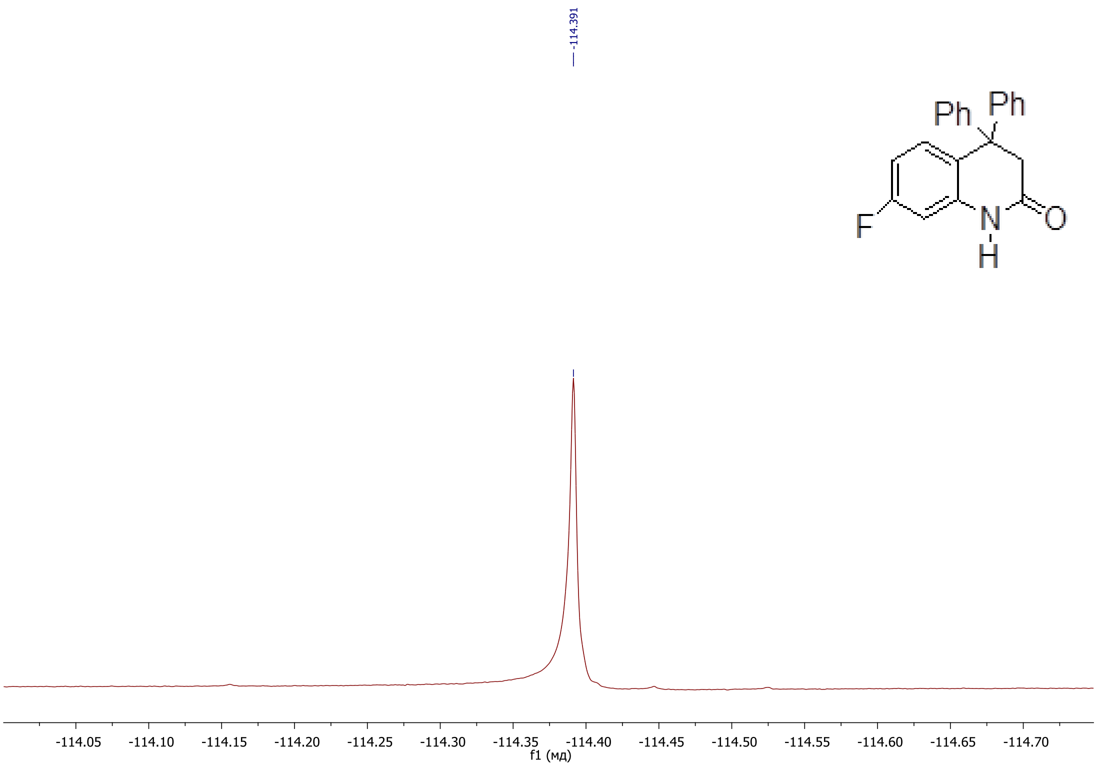

Figure S16. ^{19}F {^{1}H}NMR spectrum of the compound **2f** (376 MHz, $CDCl_3$).

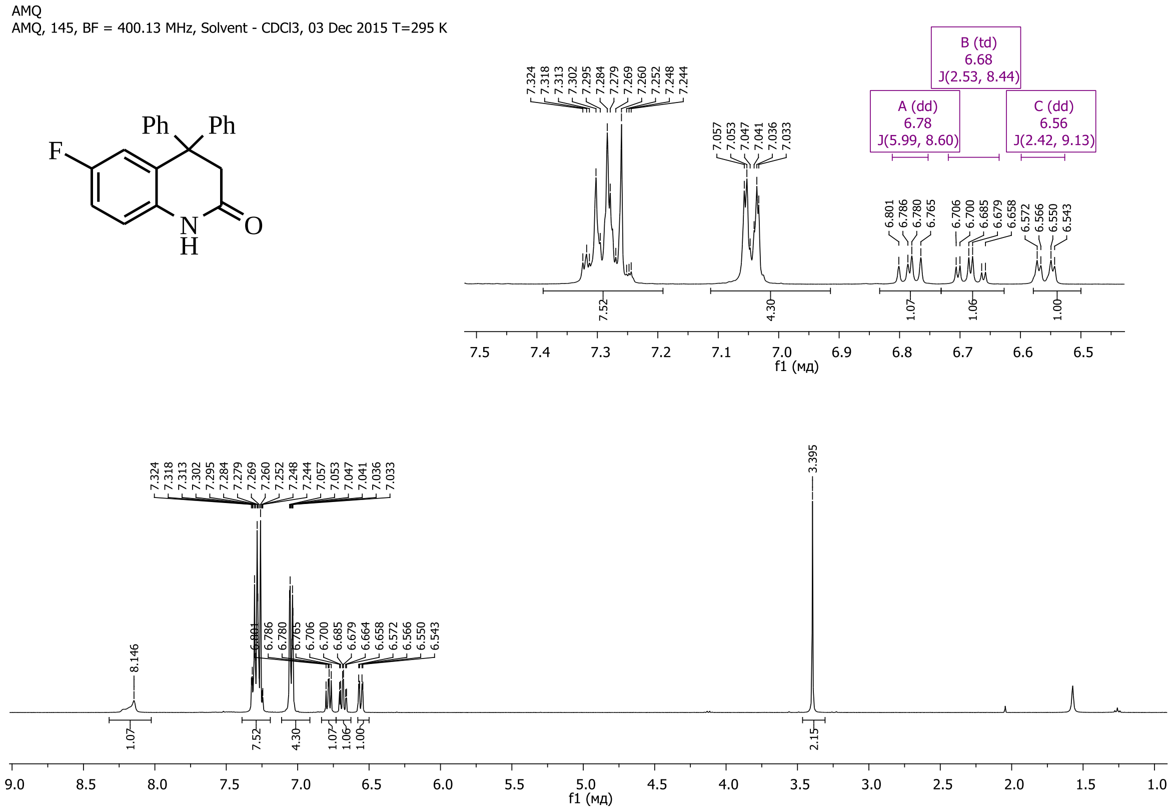

Figure S17. ^{1}H NMR spectrum of the compound **2g** (400 MHz, $CDCl_3$).

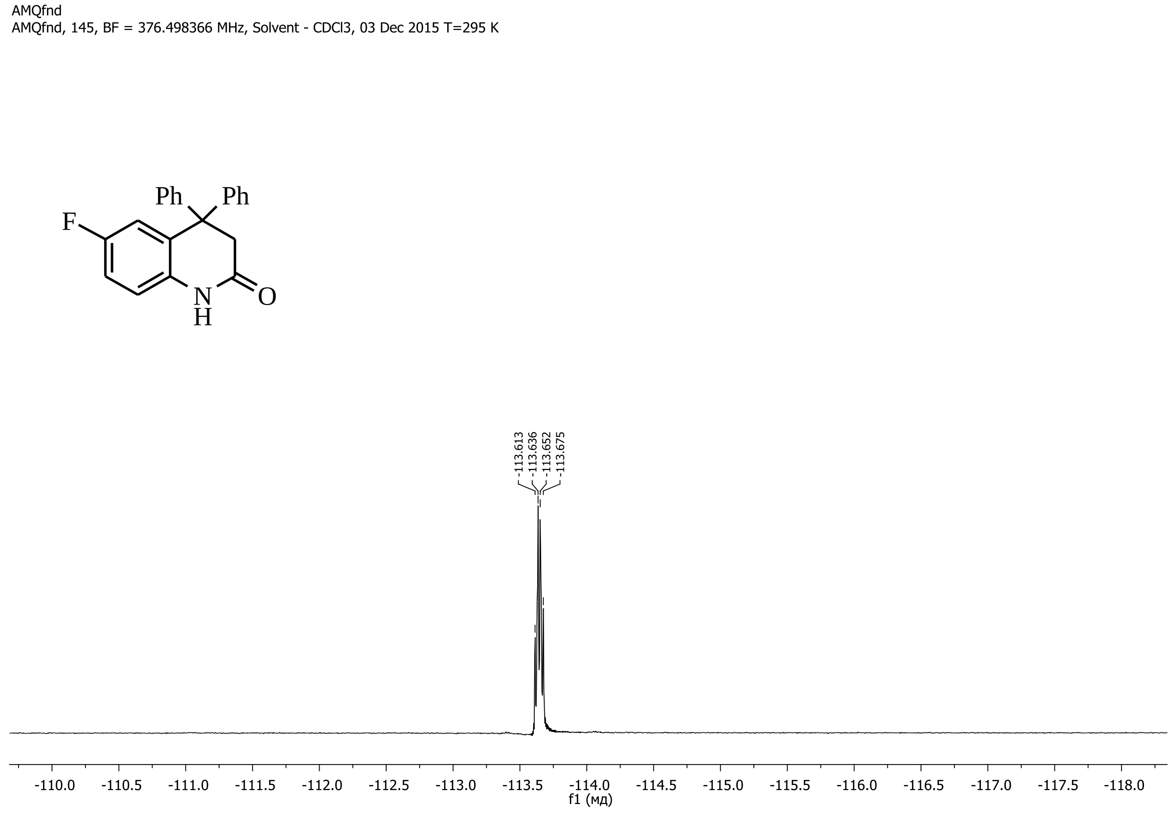

Figure S18. ^{19}F NMR spectrum of the compound **2g** (376 MHz, $CDCl_3$).

AMQc
AMQc, 145, BF = 100.612769 MHz, Solvent - CDCl3, 10 Dec 2015 T=296 K

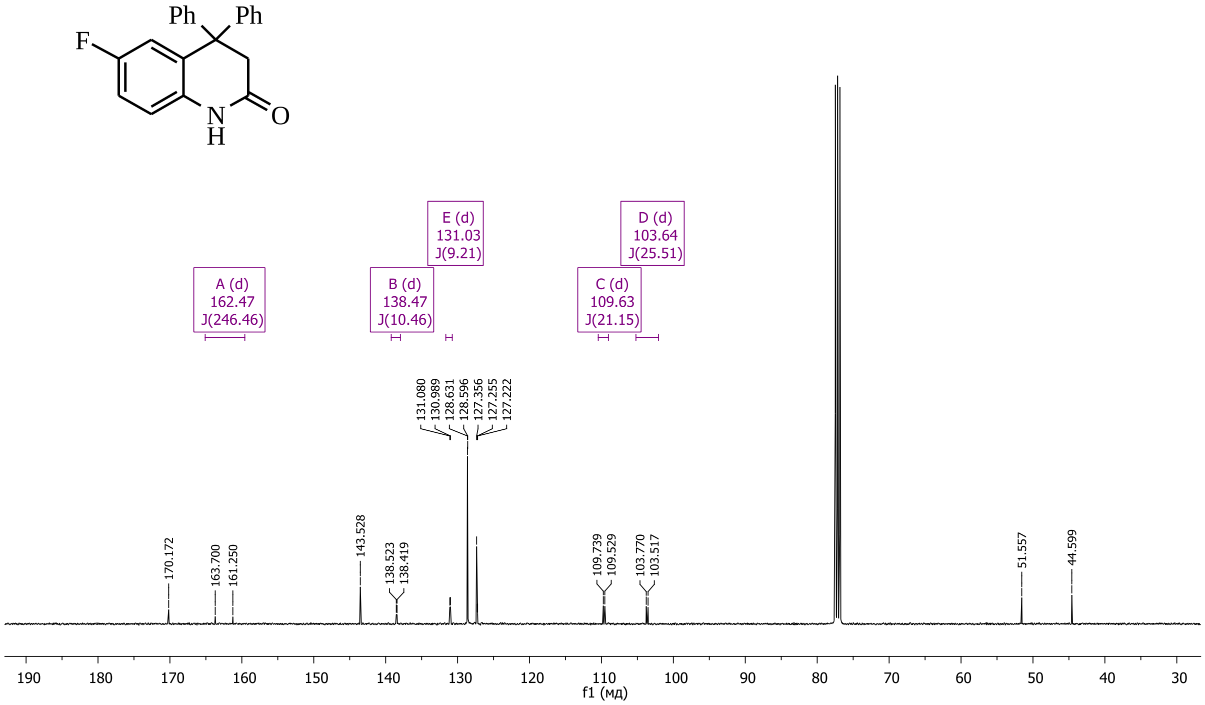

Figure S19. ^{13}C NMR spectrum of the compound **2g** (100 MHz, $CDCl_3$).

AMQd
AMQd, 145, BF = 100.612769 MHz, Solvent - CDCl3, 10 Dec 2015 T=295 K

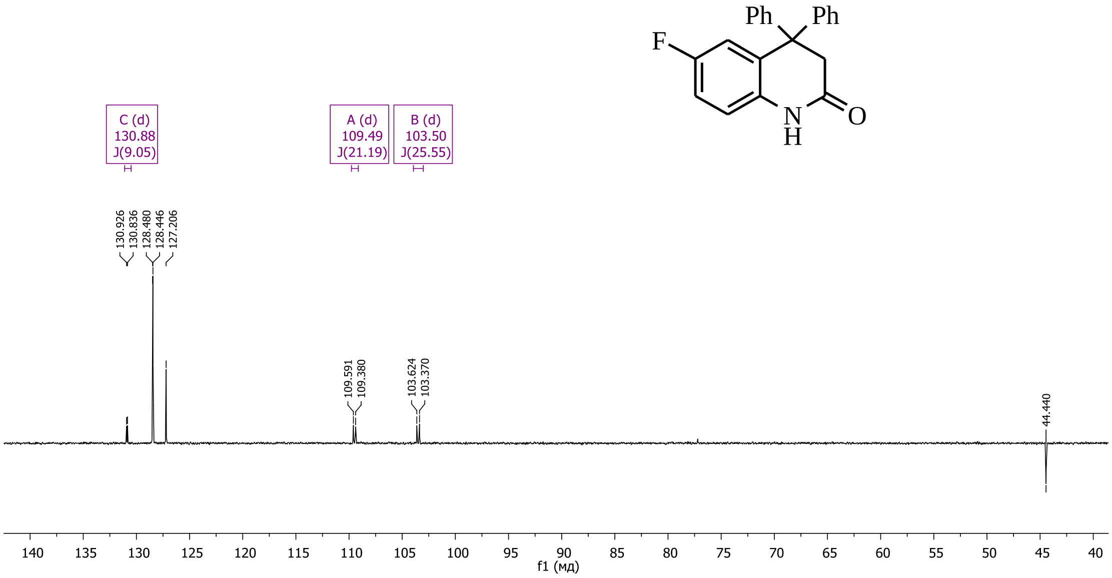

Figure S20. DEPT spectrum of the compound **2g** (100 MHz, $CDCl_3$).

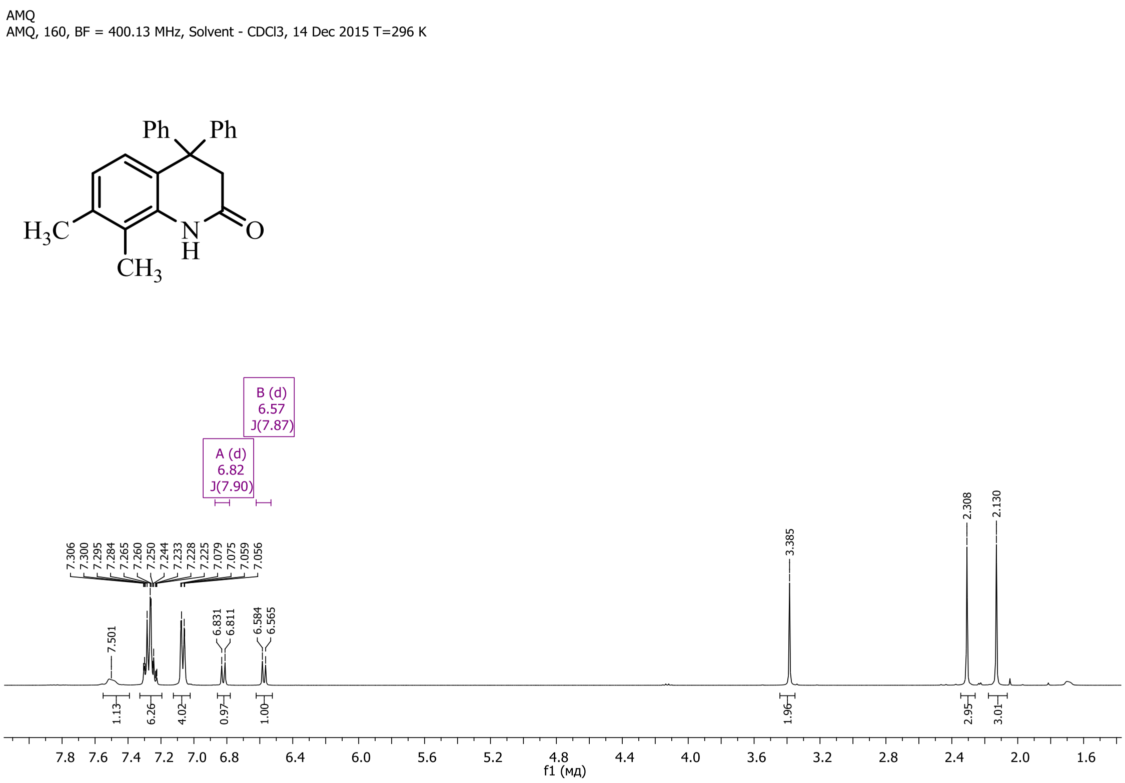

Figure S21. ^{1}H NMR spectrum of the compound **2i** (400 MHz, $CDCl_3$).

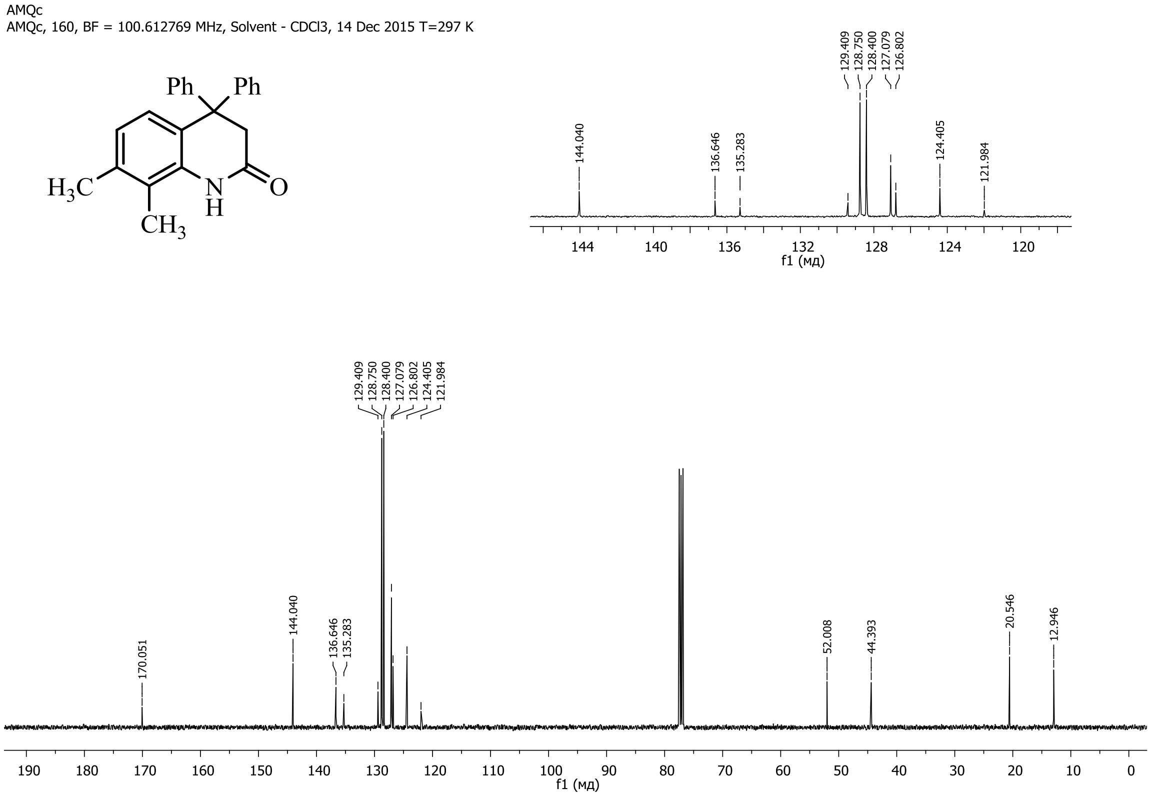

Figure S22. ^{13}C NMR spectrum of the compound **2i** (100 MHz, $CDCl_3$).

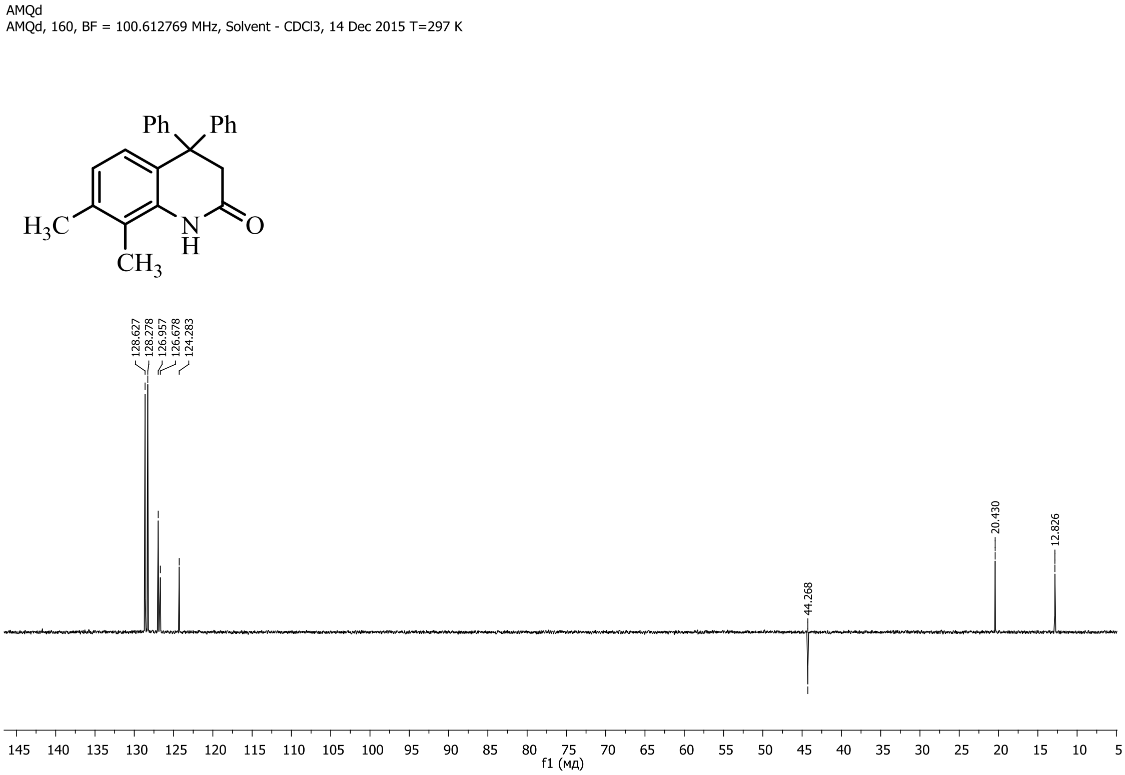

Figure S23. DEPT spectrum of the compound **2i** (100 MHz, $CDCl_3$).

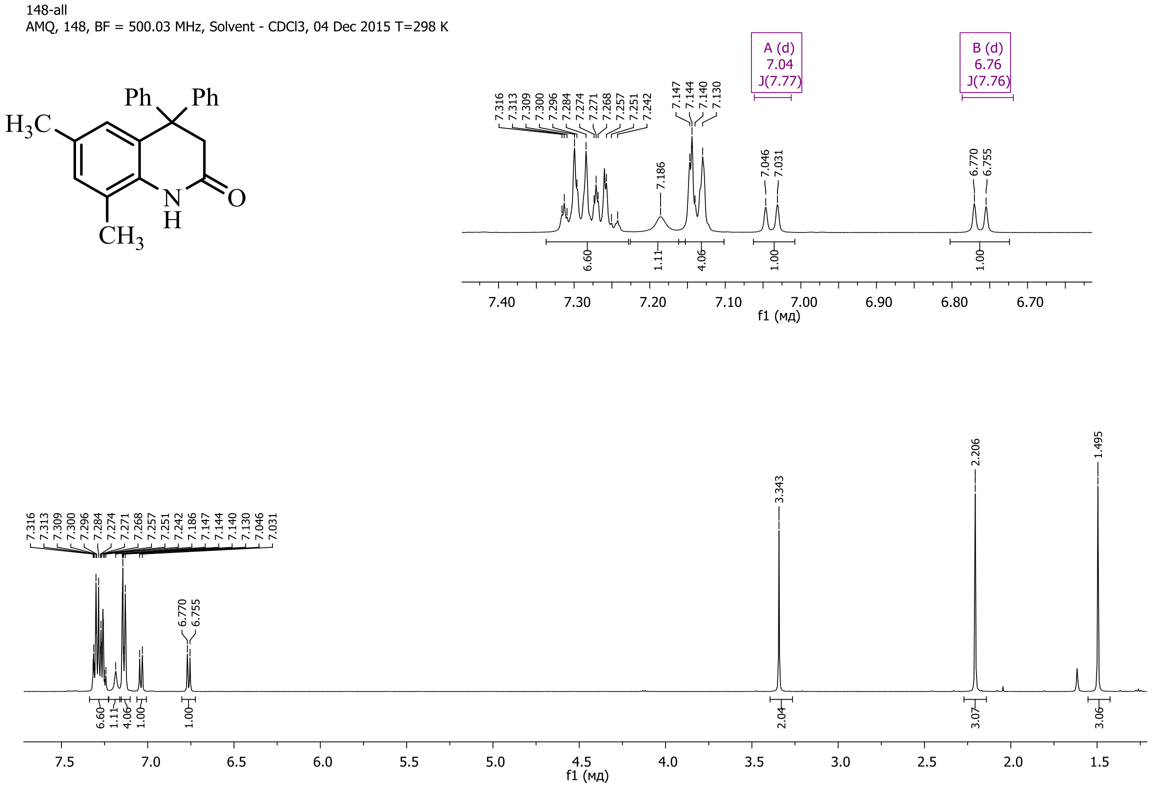

Figure S24. ^{1}H NMR spectrum of the compound **2j** (400 MHz, $CDCl_3$).

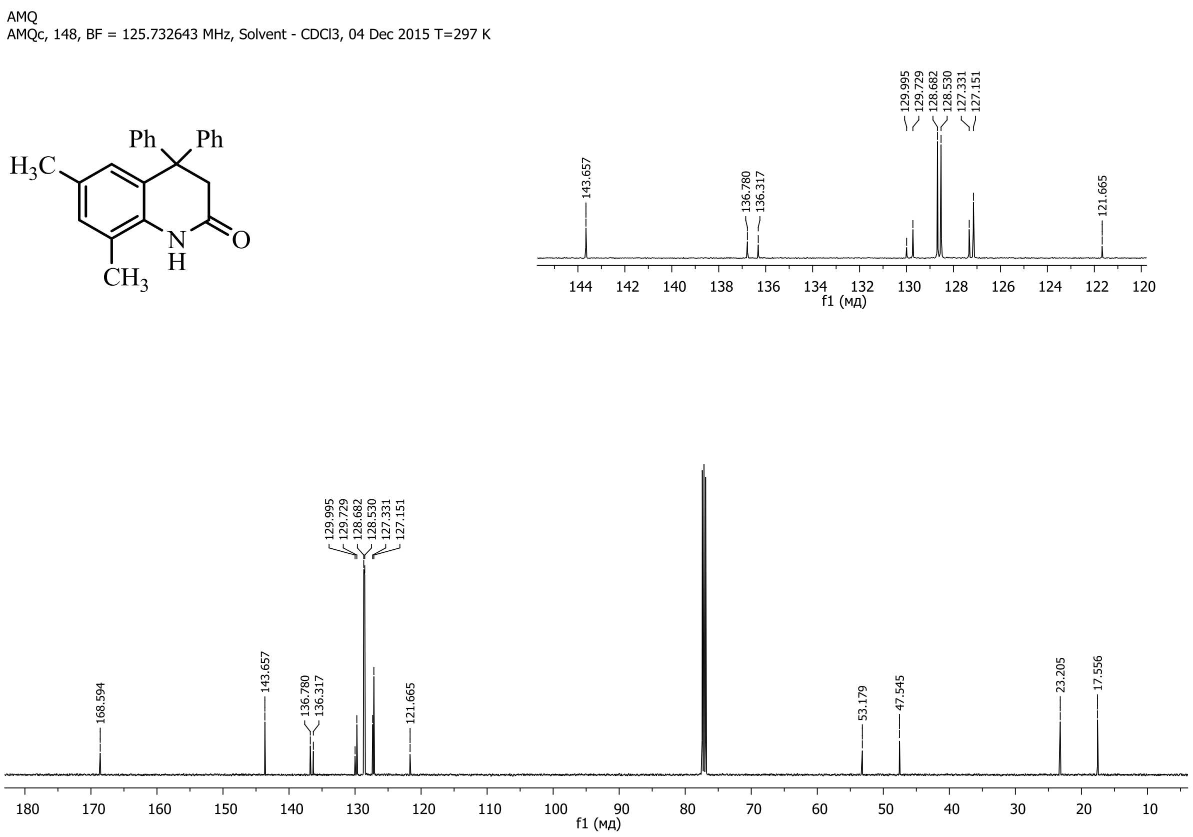

Figure S25. ^{13}C NMR spectrum of the compound **2j** (100 MHz, $CDCl_3$).

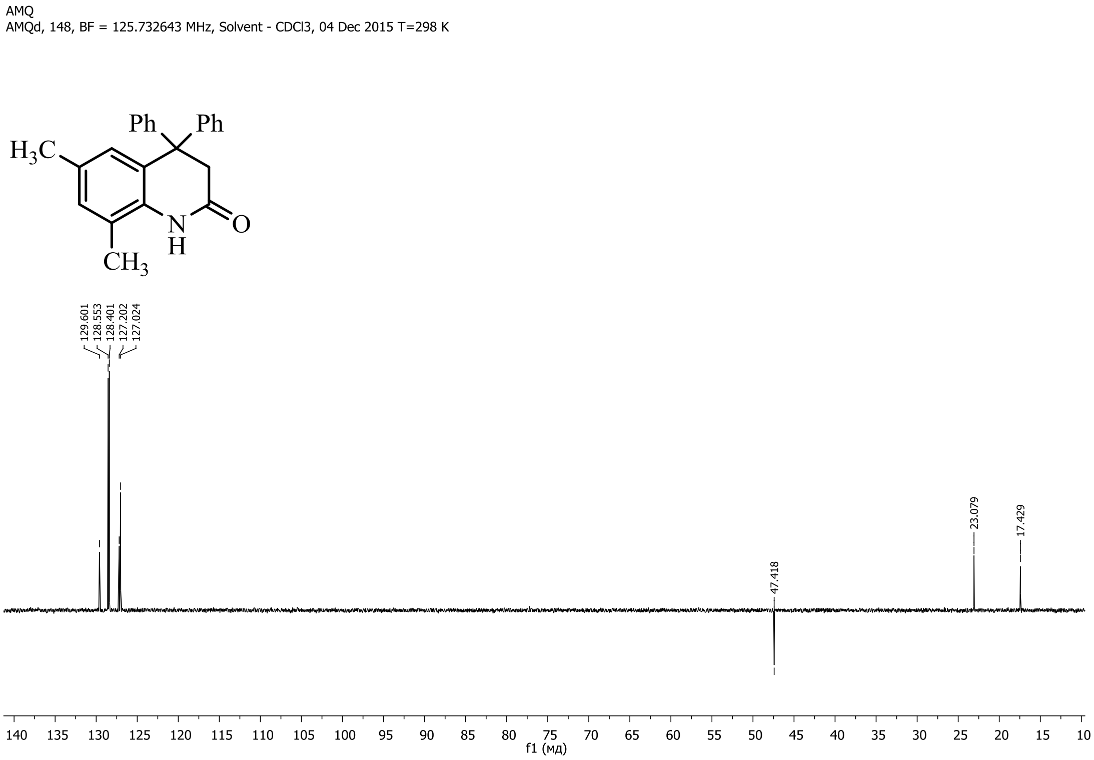

Figure S26. DEPT spectrum of the compound **2j** (100 MHz, $CDCl_3$).

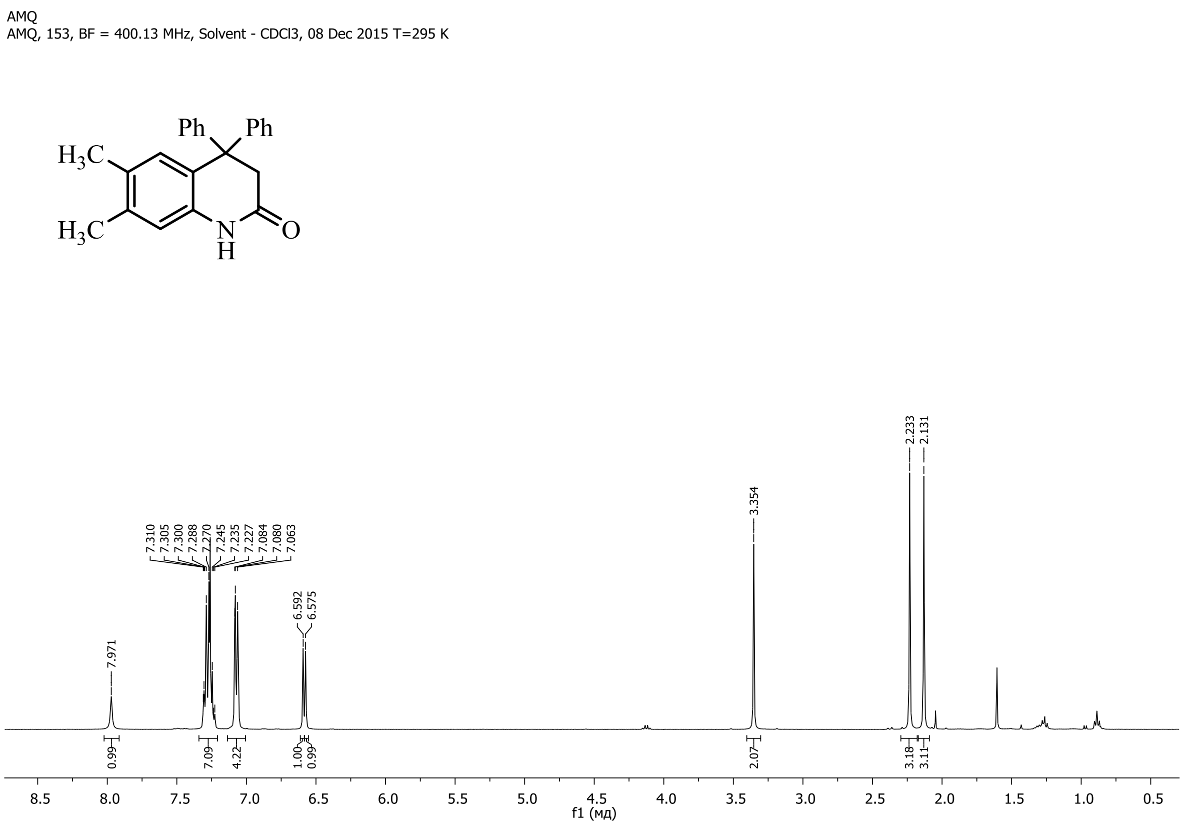

Figure S27. ^{1}H NMR spectrum of the compound **2k** (400 MHz, $CDCl_3$).

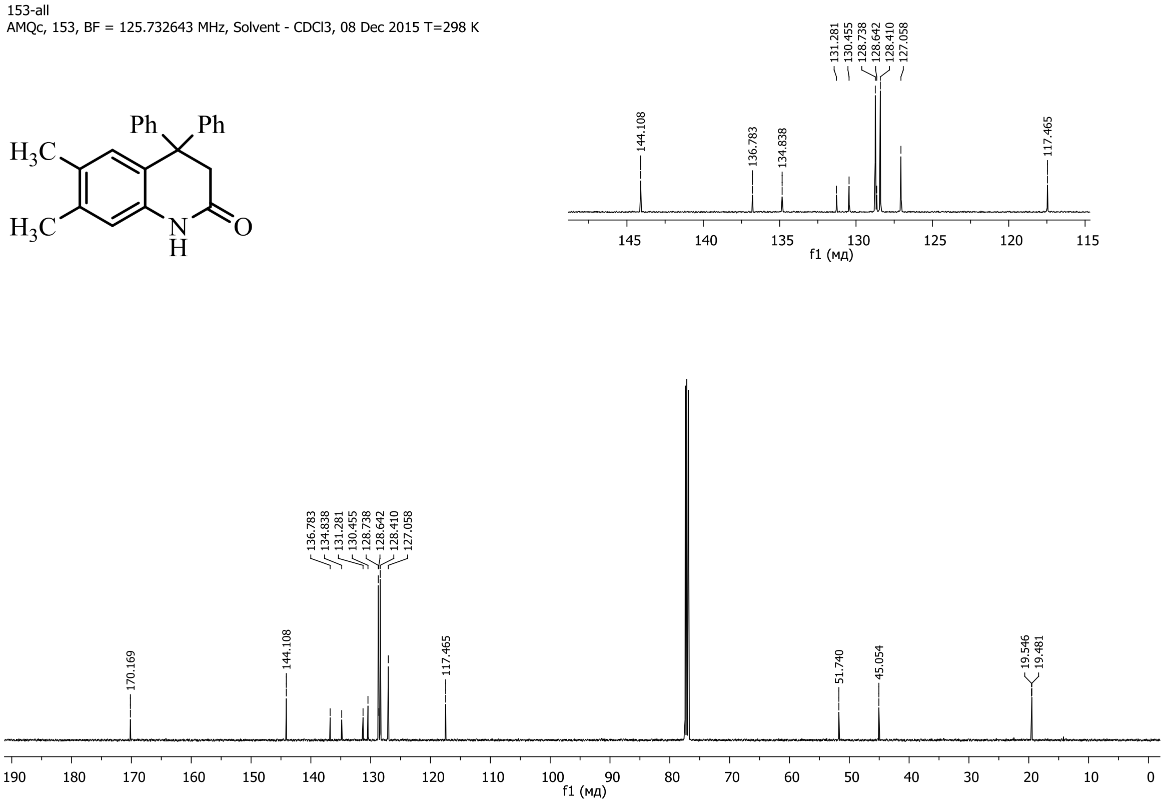

Figure S28. ^{13}C NMR spectrum of the compound **2k** (100 MHz, $CDCl_3$).

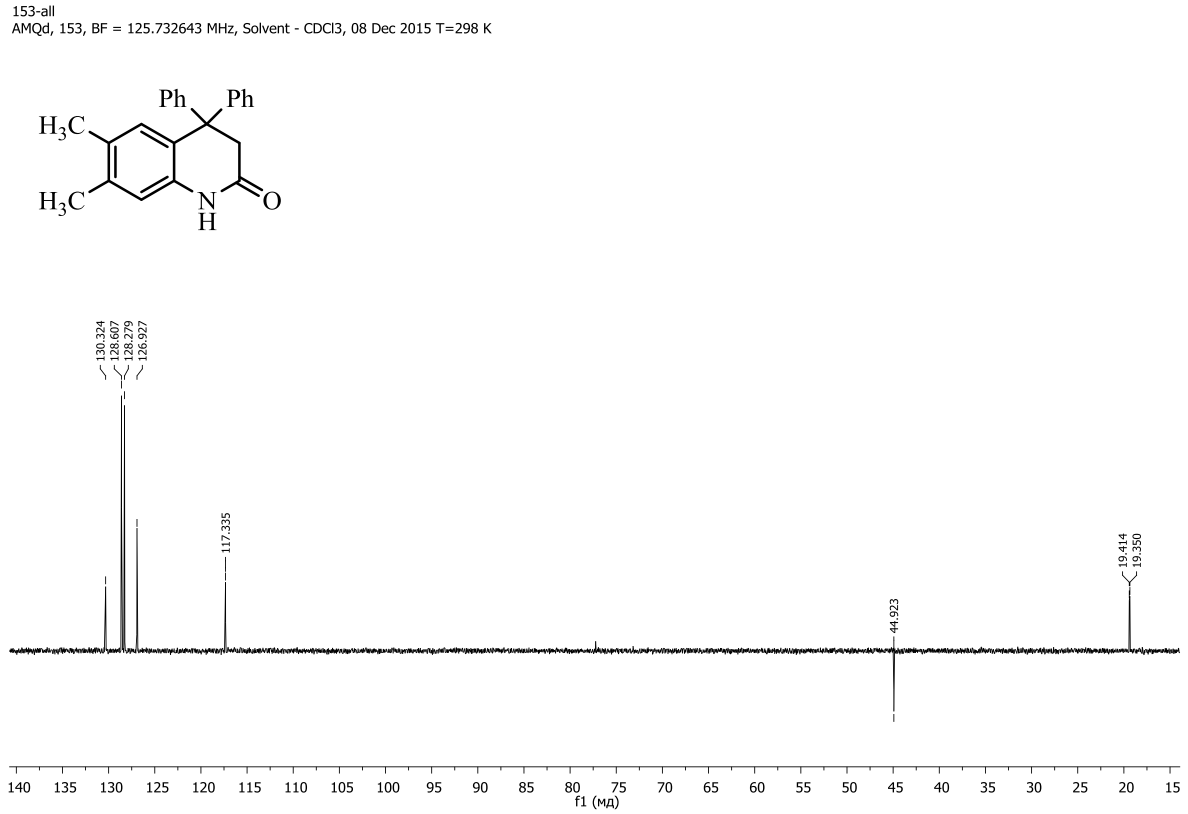

Figure S29. DEPT spectrum of the compound **2k** (100 MHz, $CDCl_3$).

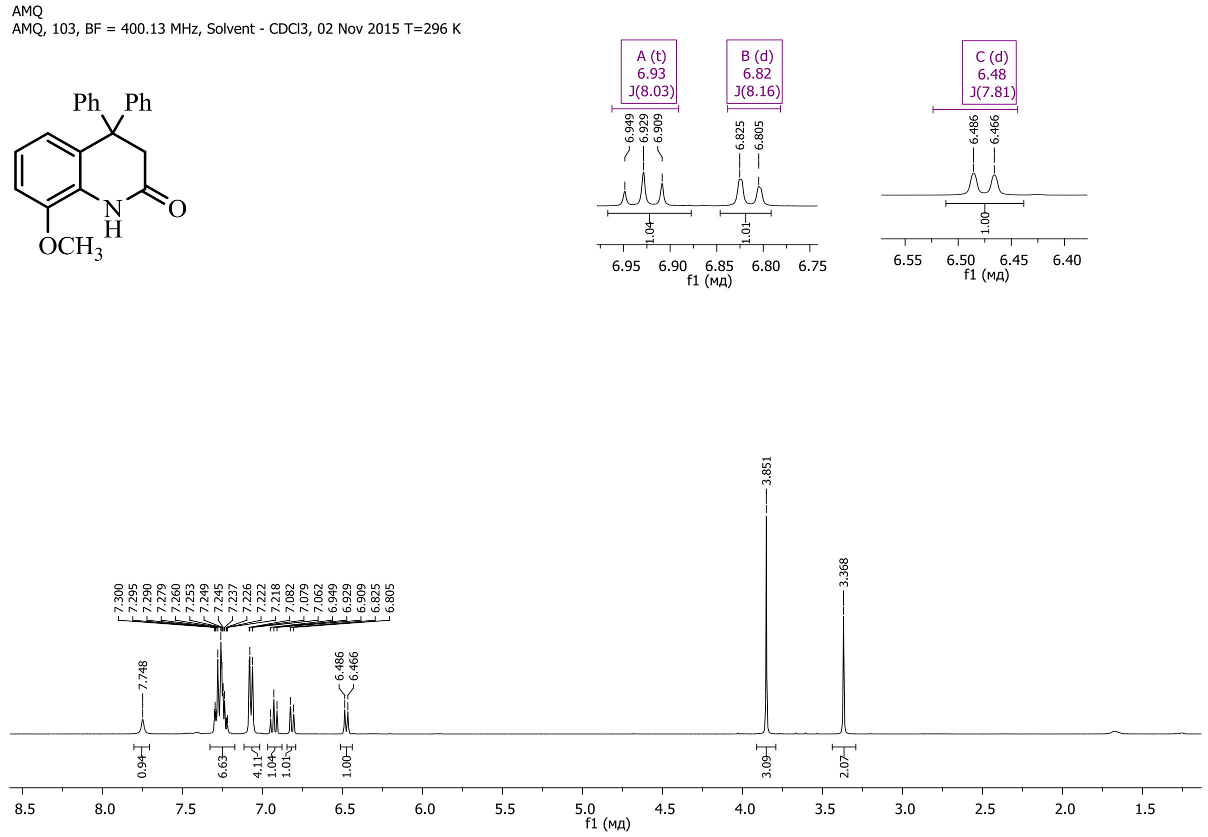

Figure S30. ^{1}H NMR spectrum of the compound **2l** (400 MHz, $CDCl_3$).

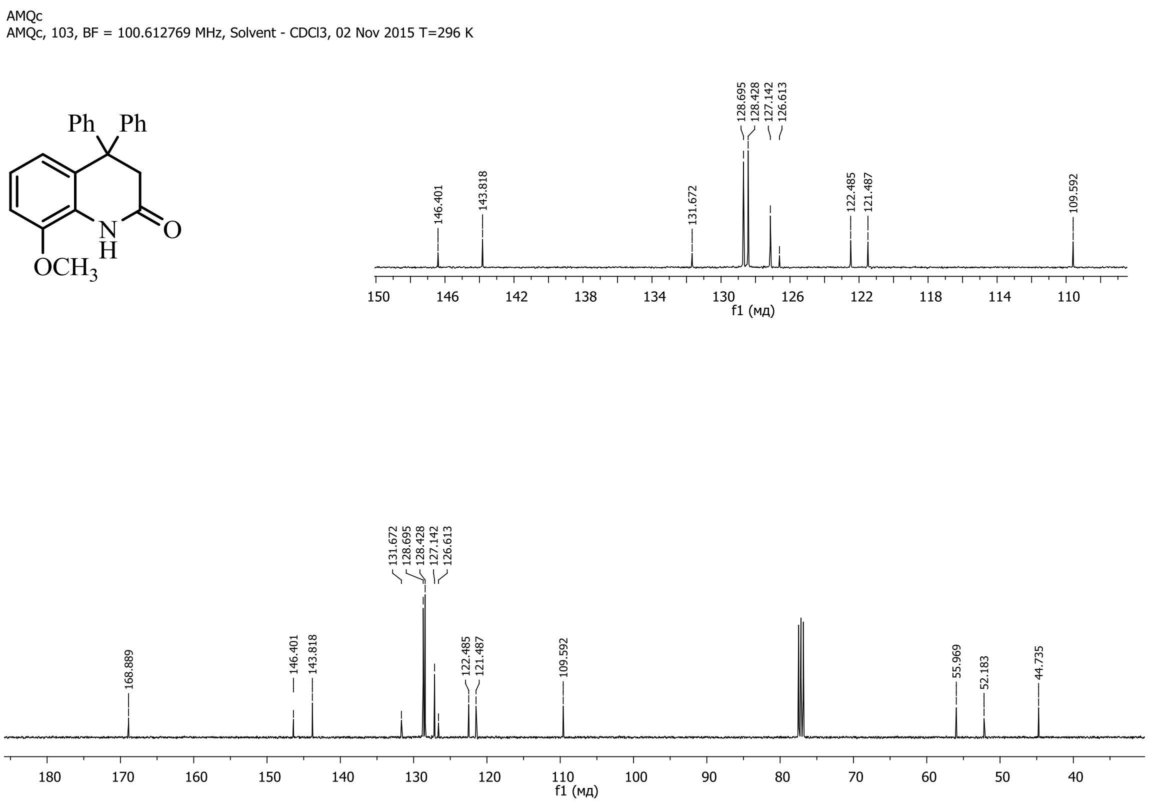

Figure S31. ^{13}C NMR spectrum of the compound **2l** (100 MHz, $CDCl_3$).

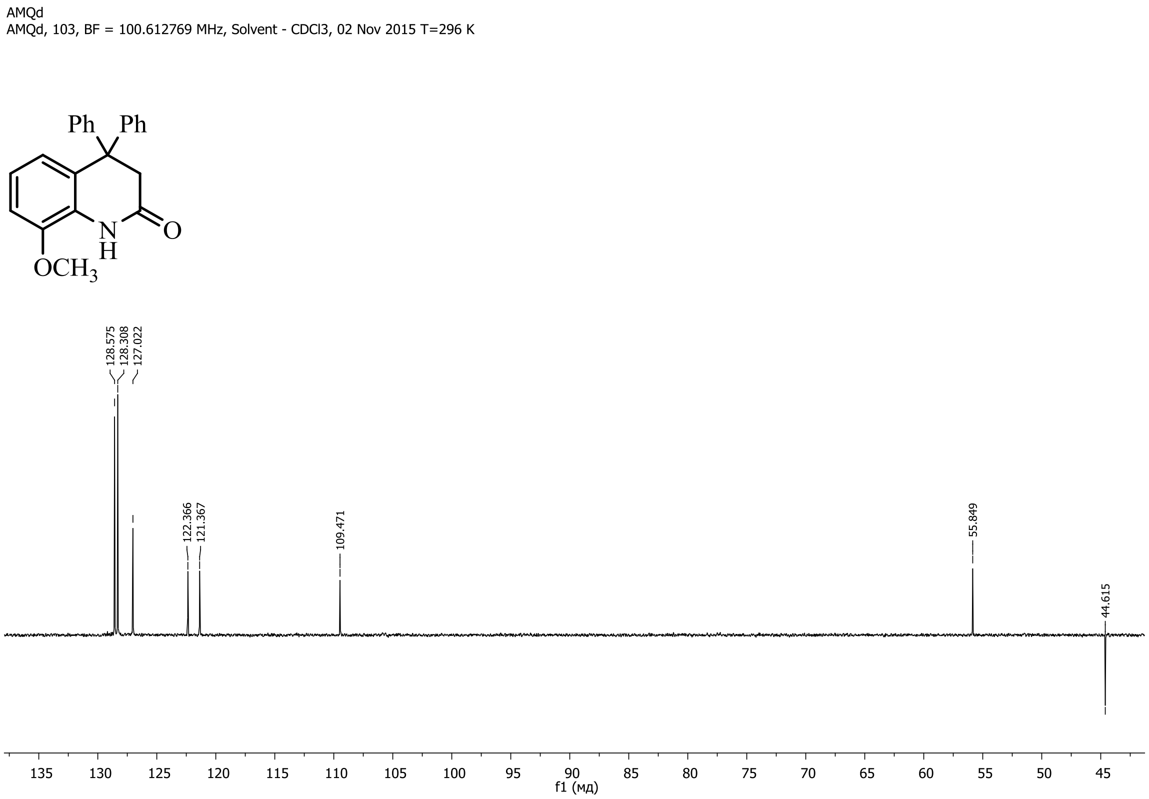

Figure S32. DEPT spectrum of the compound **2l** (100 MHz, $CDCl_3$).

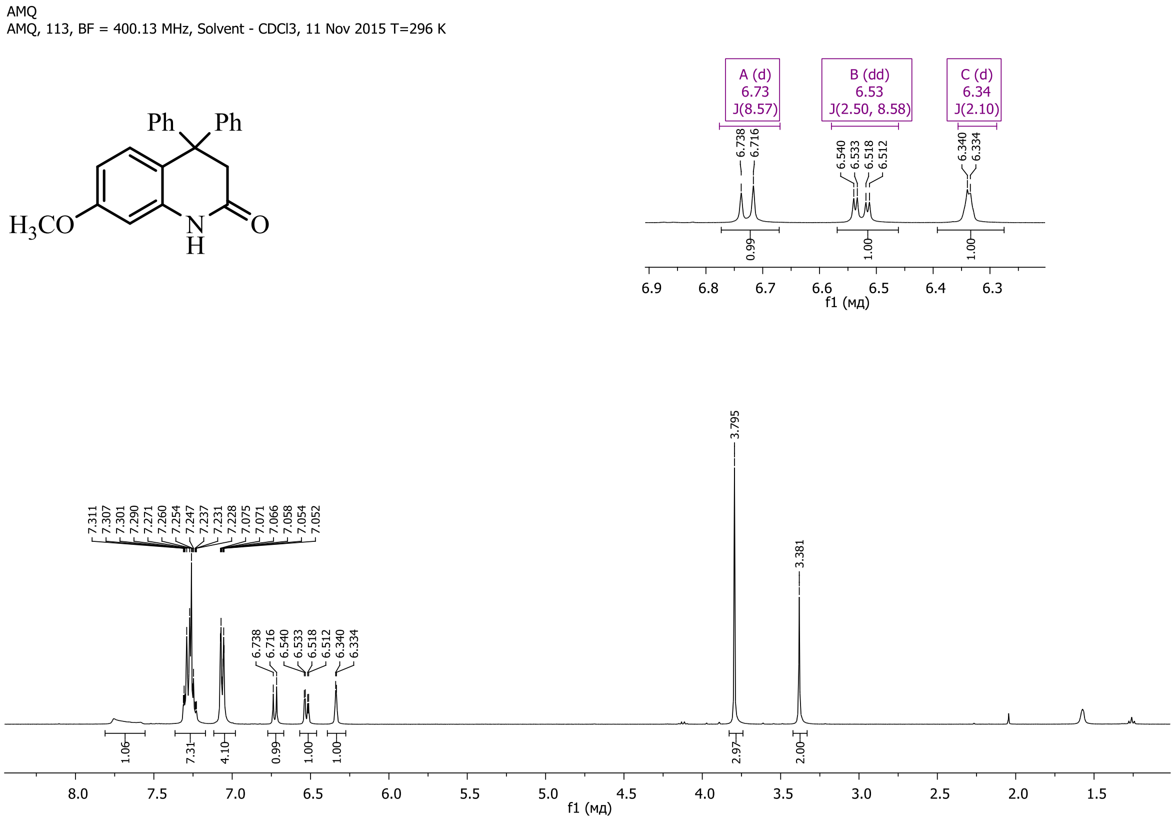

Figure S33. ^{1}H NMR spectrum of the compound **2m** (400 MHz, $CDCl_3$).

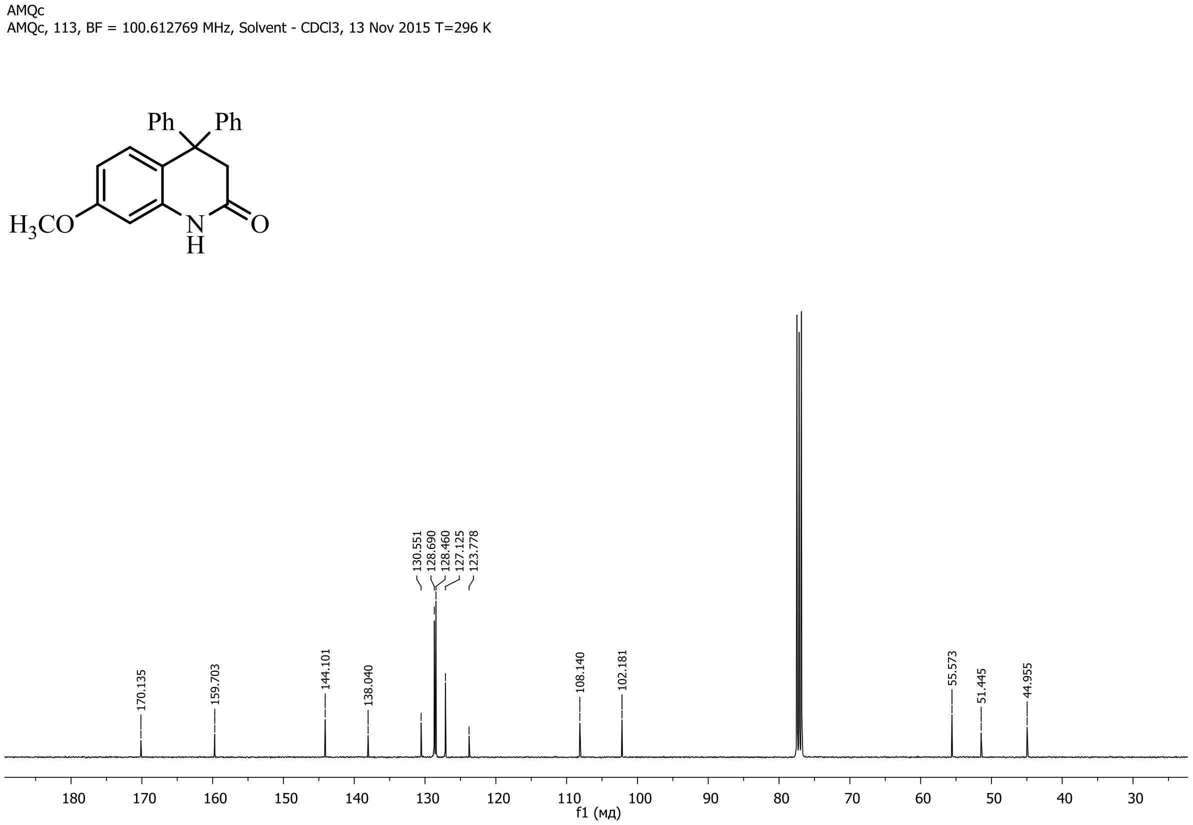

Figure S34. ^{13}C NMR spectrum of the compound **2m** (100 MHz, $CDCl_3$).

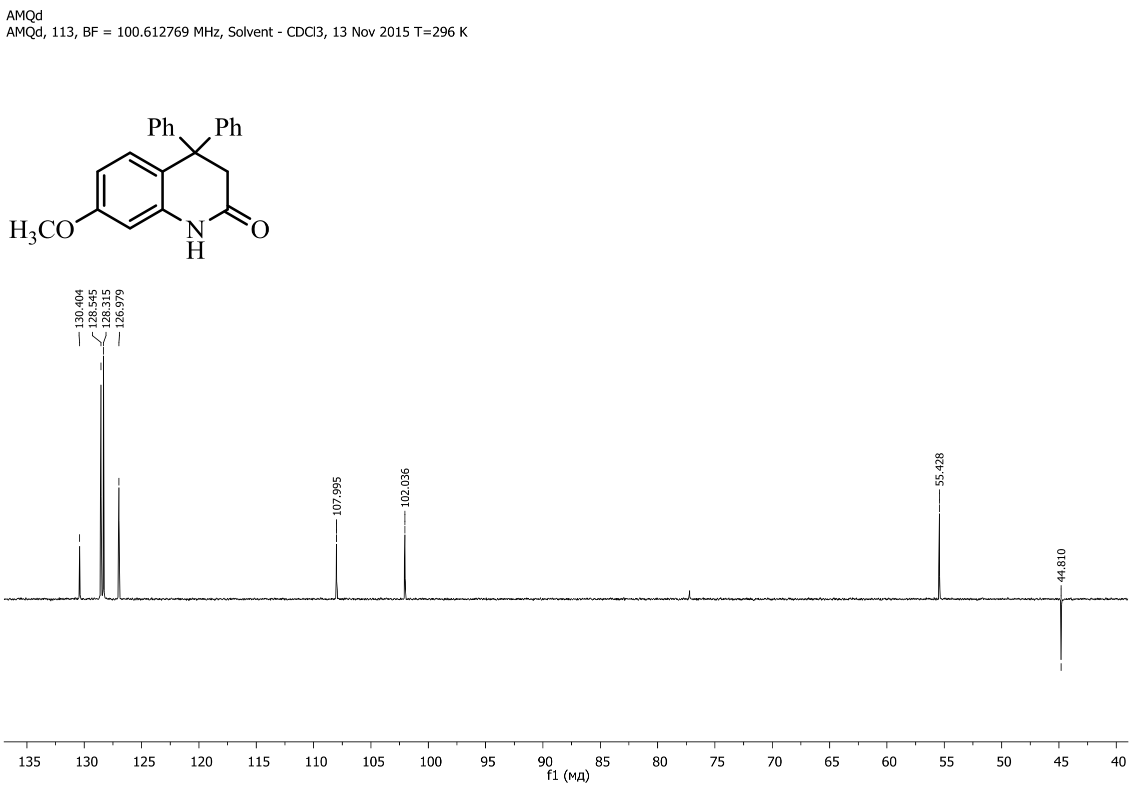

Figure S35. DEPT spectrum of the compound **2m** (100 MHz, $CDCl_3$).

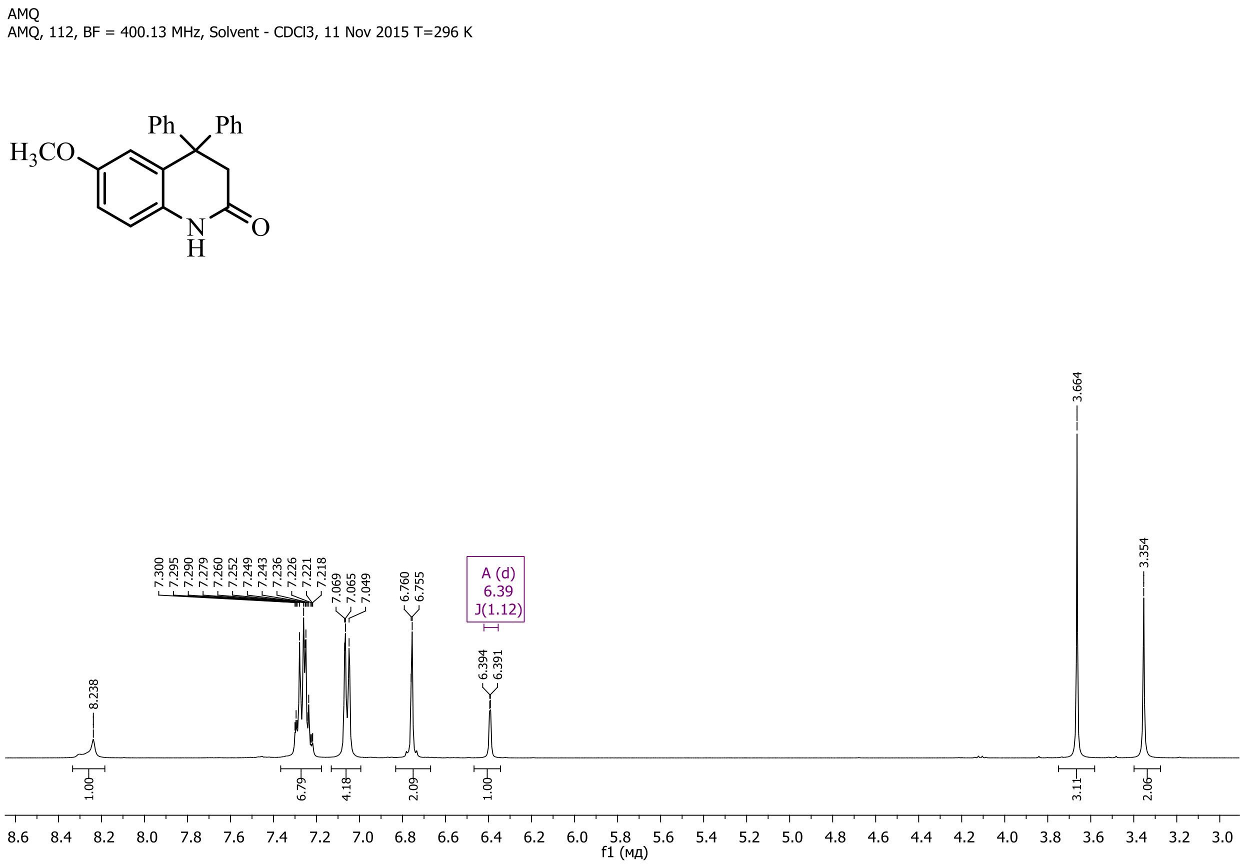

Figure S36. ^{1}H NMR spectrum of the compound **2n** (400 MHz, $CDCl_3$).

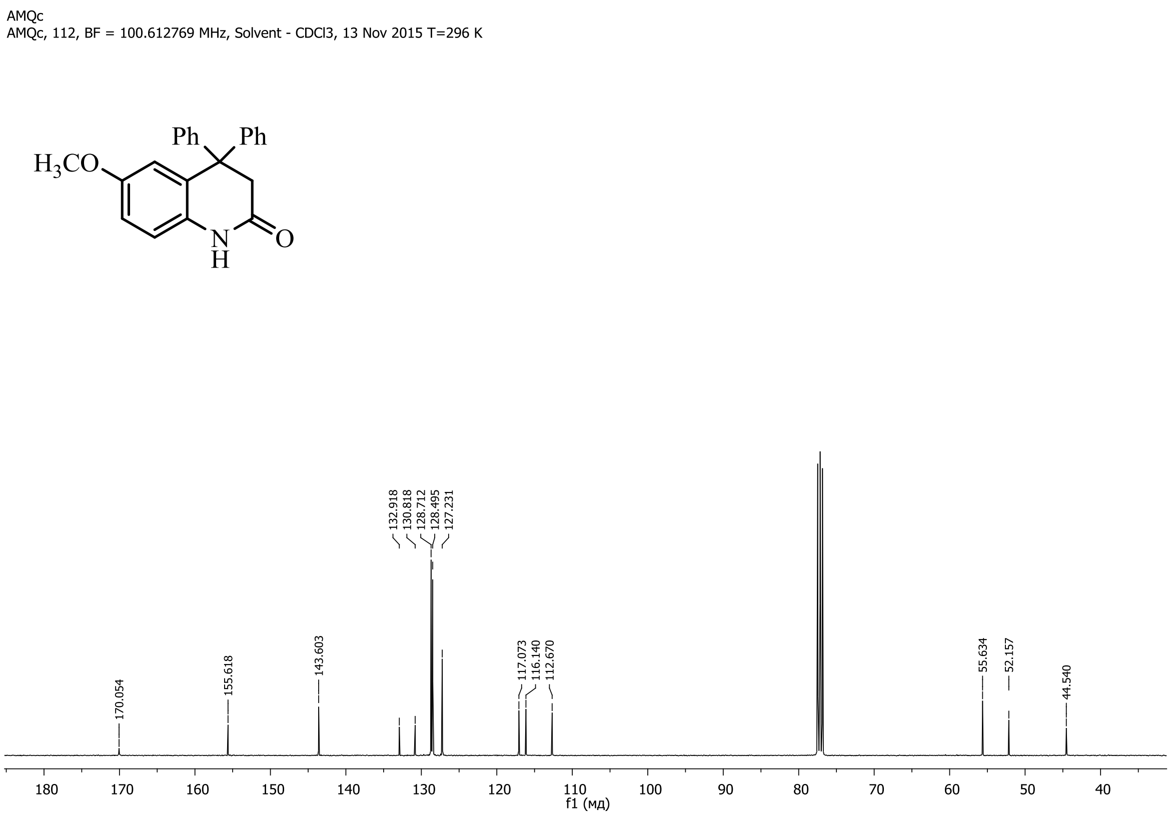

Figure S37. ^{13}C NMR spectrum of the compound **2n** (100 MHz, $CDCl_3$).

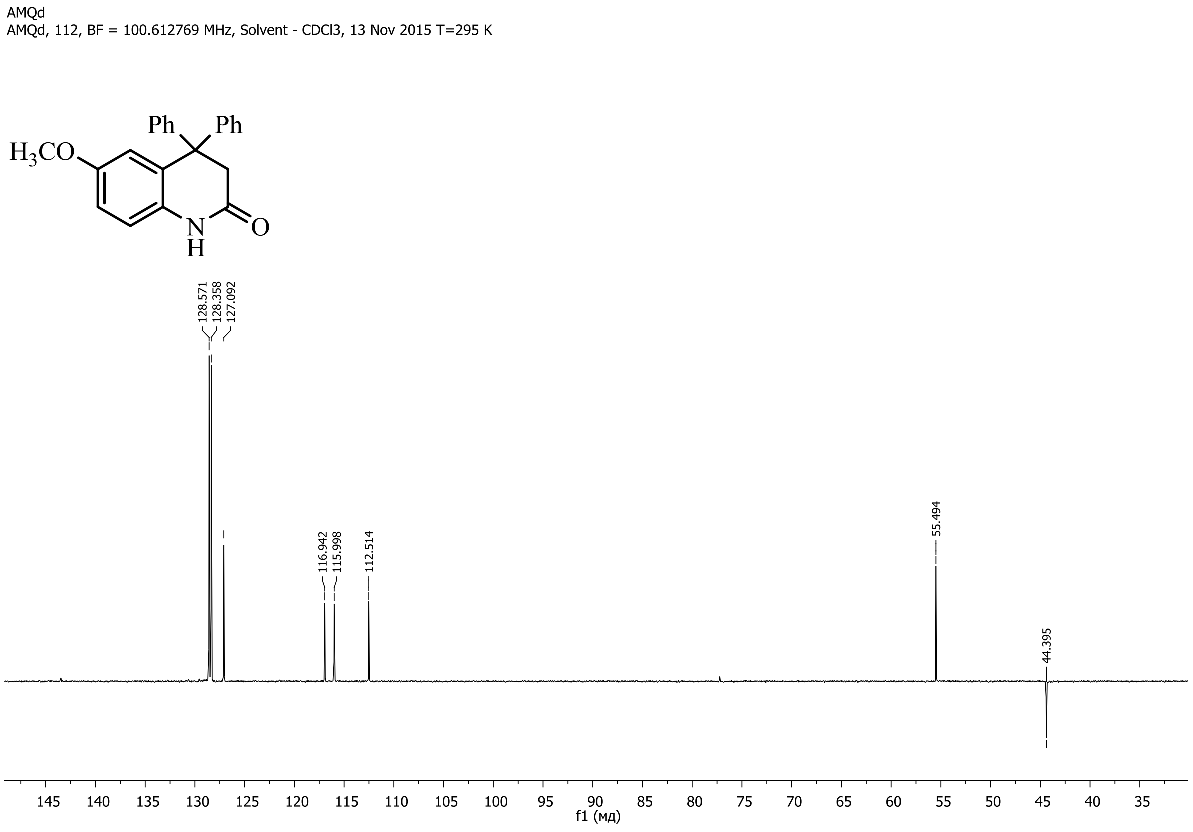

Figure S38. DEPT spectrum of the compound **2n** (100 MHz, $CDCl_3$).

AMQ
AMQ, 169, BF = 400.13 MHz, Solvent - CDCl3, 18 Dec 2015 T=296 K

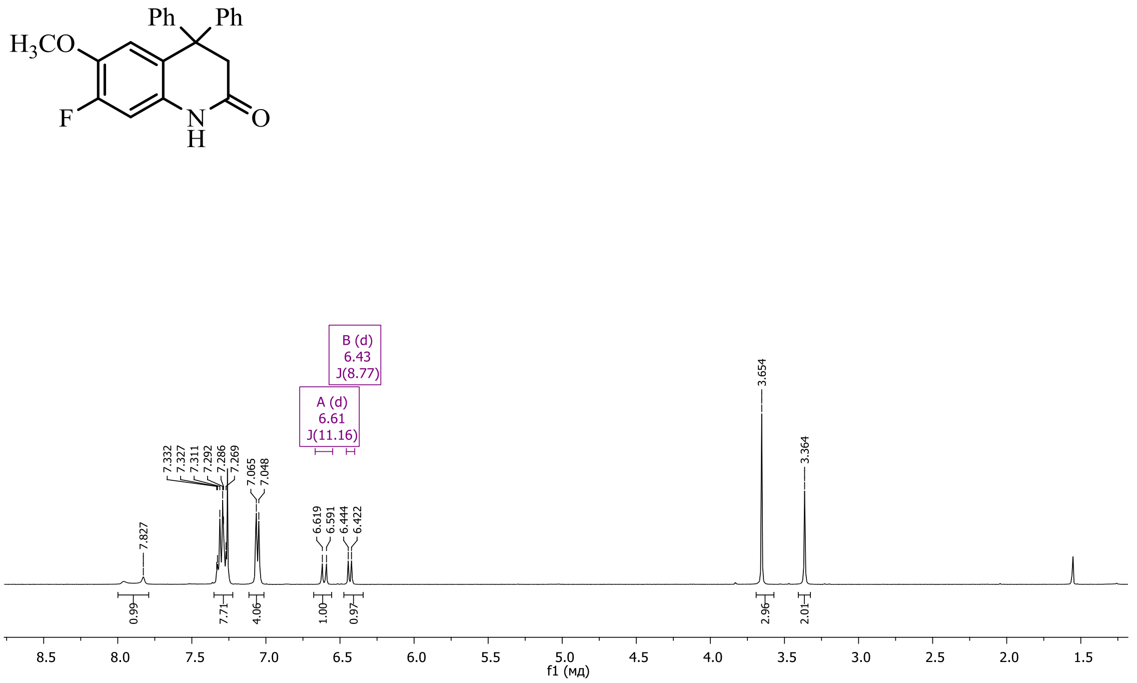

Figure S39. ^{1}H NMR spectrum of the compound **2o** (400 MHz, $CDCl_3$).

AMQfnd
AMQfnd, 173, BF = 376.498366 MHz, Solvent - CDCl3, 22 Dec 2015 T=296 K

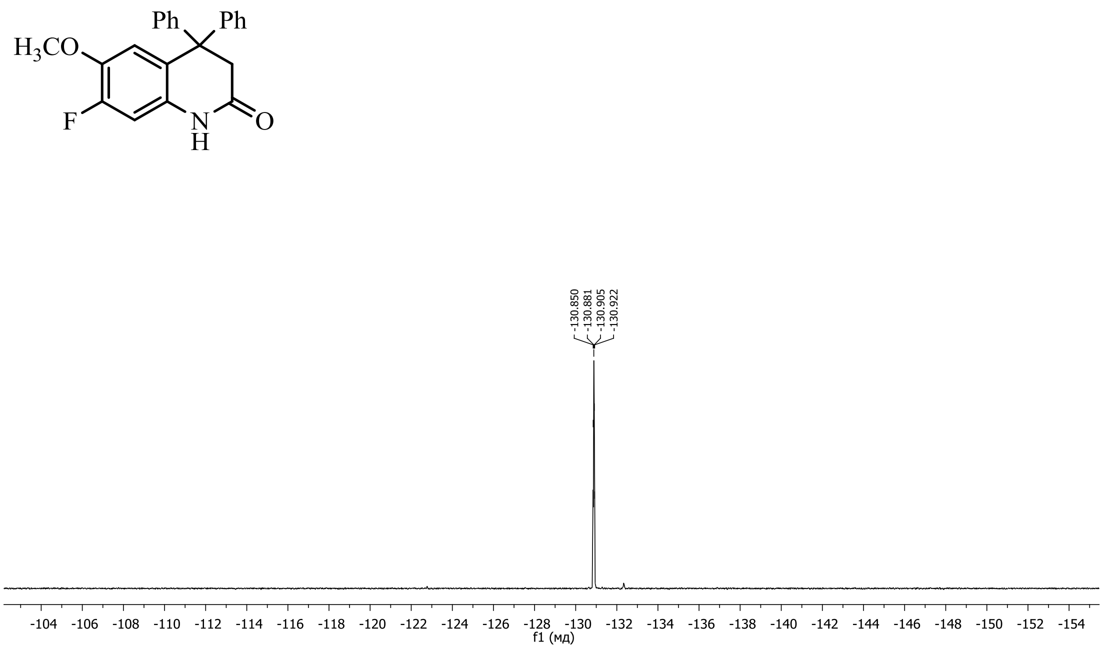

Figure S40. ^{19}F NMR spectrum of the compound **2o** (376 MHz, DMSO-d6).

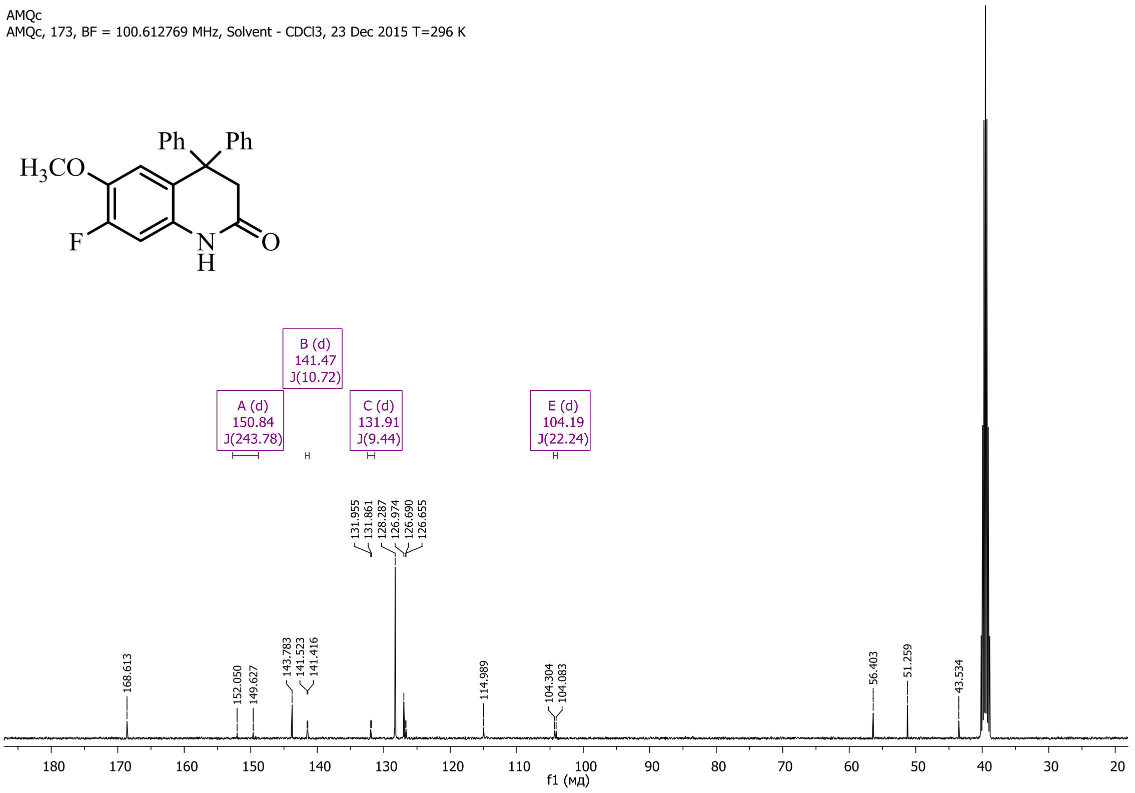

Figure S41. ^{13}C NMR spectrum of the compound **2o** (100 MHz, DMSO-d*6*).

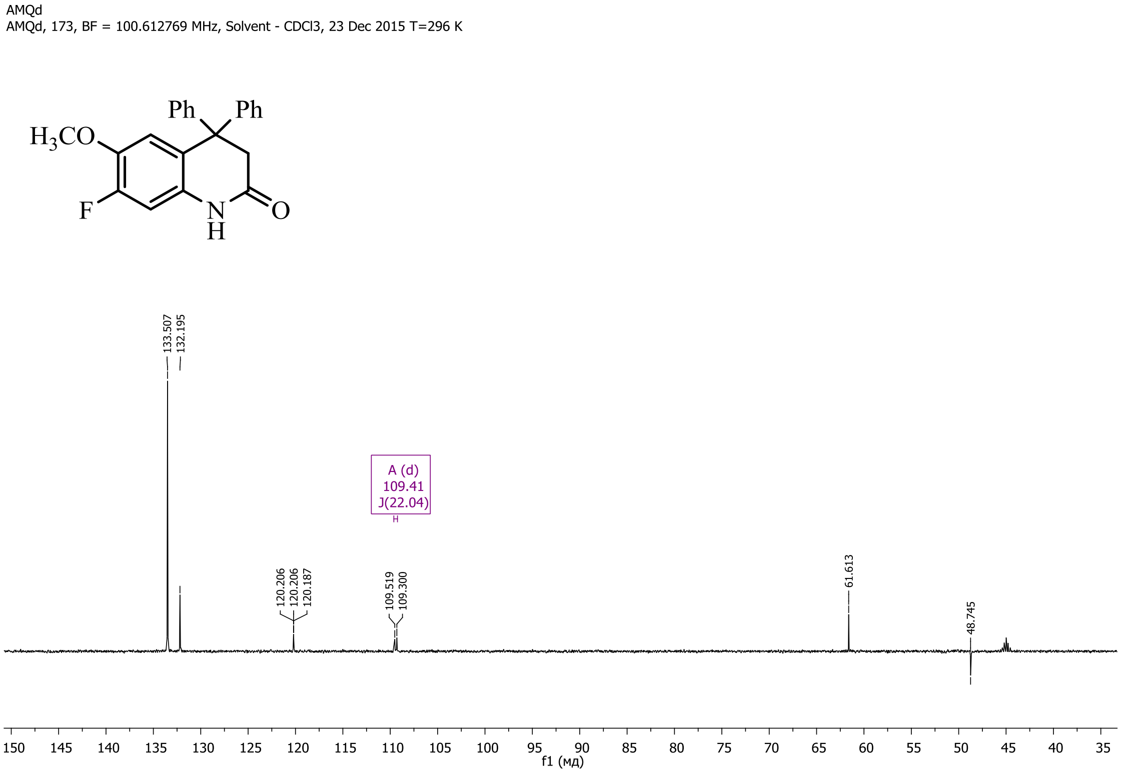

Figure S42. DEPT spectrum of the compound **2o** (100 MHz, DMSO-d*6*).

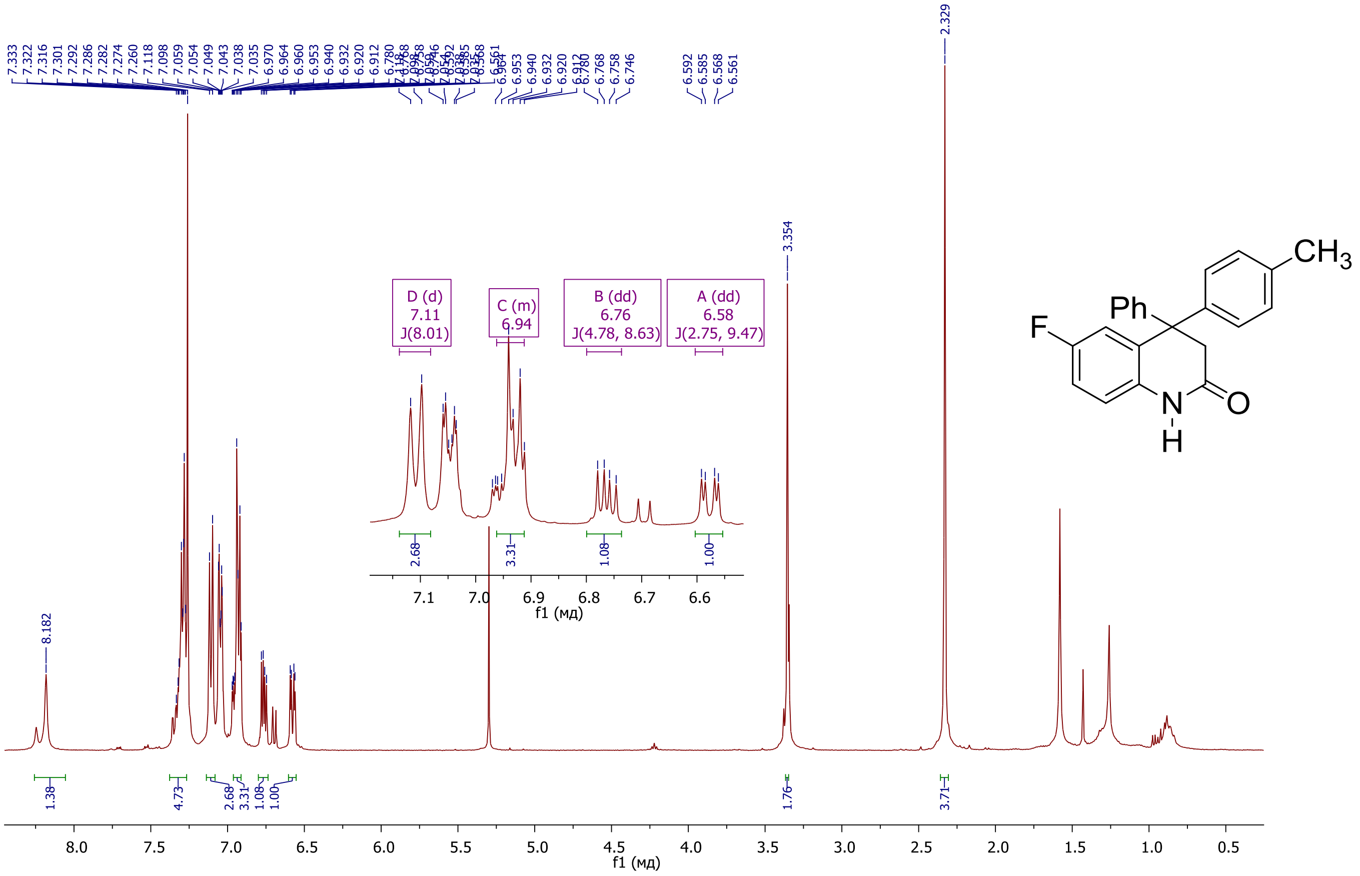

Figure S43. ^{1}H NMR spectrum of the compound **2q** (400 MHz, $CDCl_3$).

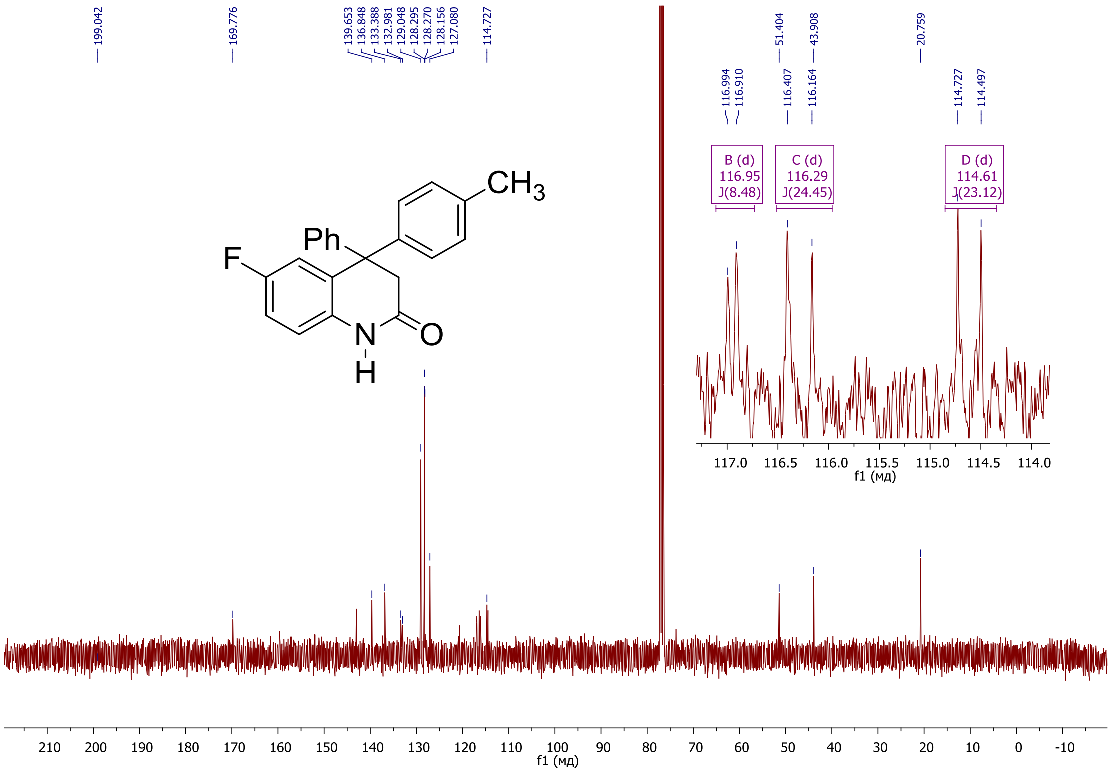

Figure S44. ^{13}C NMR spectrum of the compound **2q** (100 MHz, $CDCl_3$).

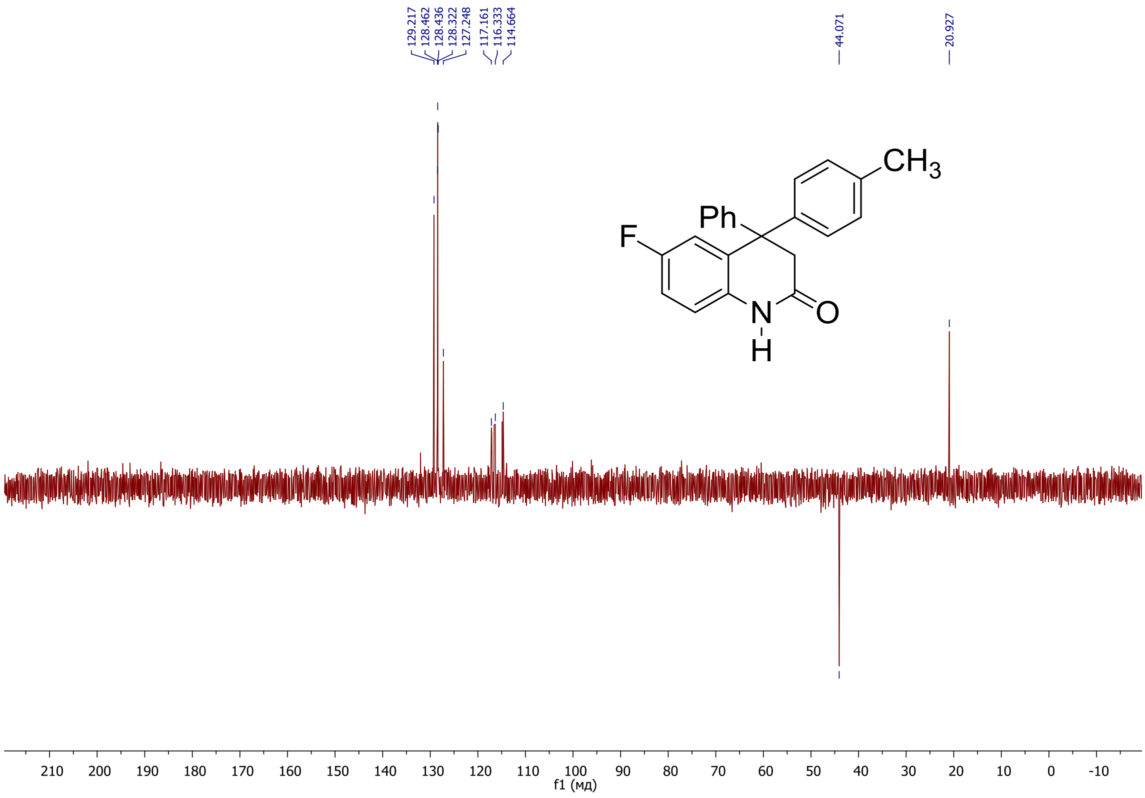

Figure S45. DEPT spectrum of the compound **2q** (100 MHz, $CDCl_3$).

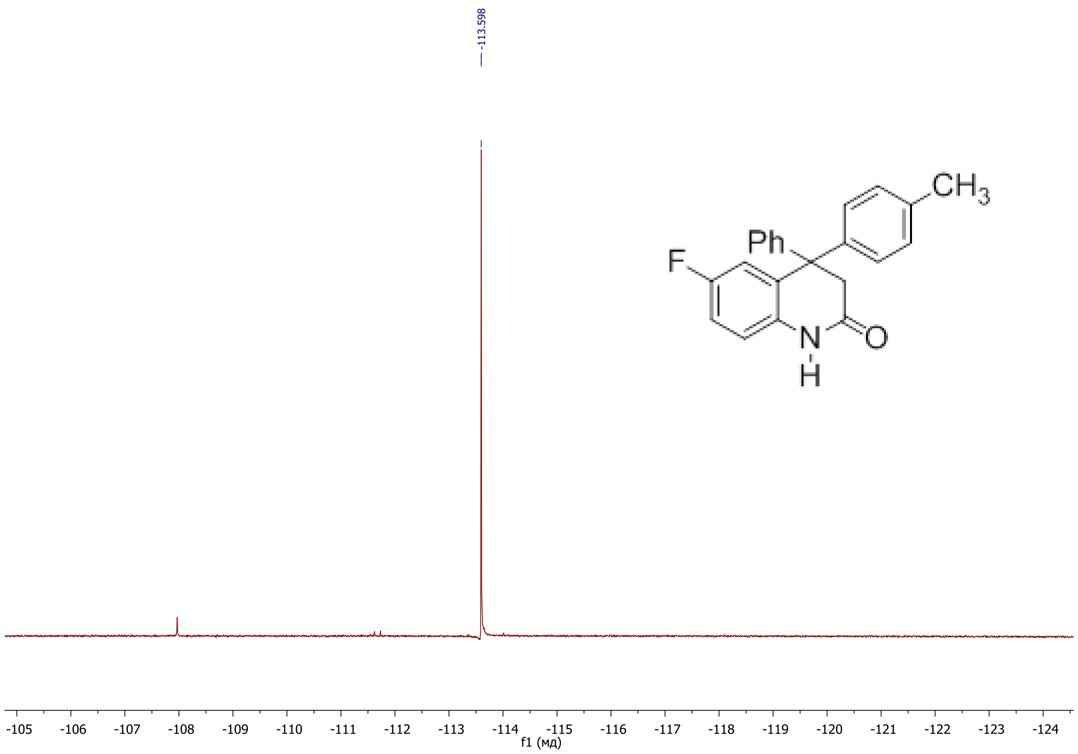

Figure S46. $^{19}F\{^1H\}$ NMR spectrum of the compound **2q** (376 MHz, $CDCl_3$).

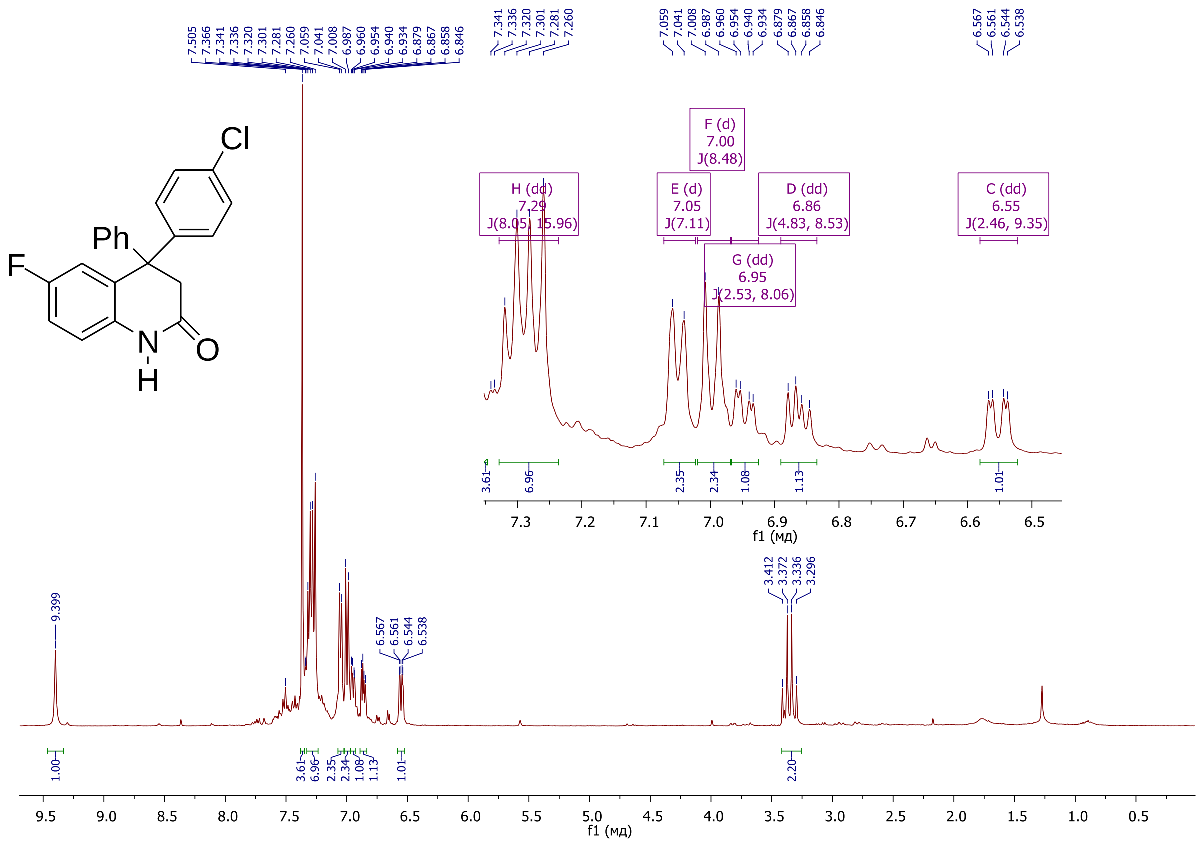

Figure S47. ^{1}H NMR spectrum of the compound **2s** (400 MHz, $CDCl_3$).

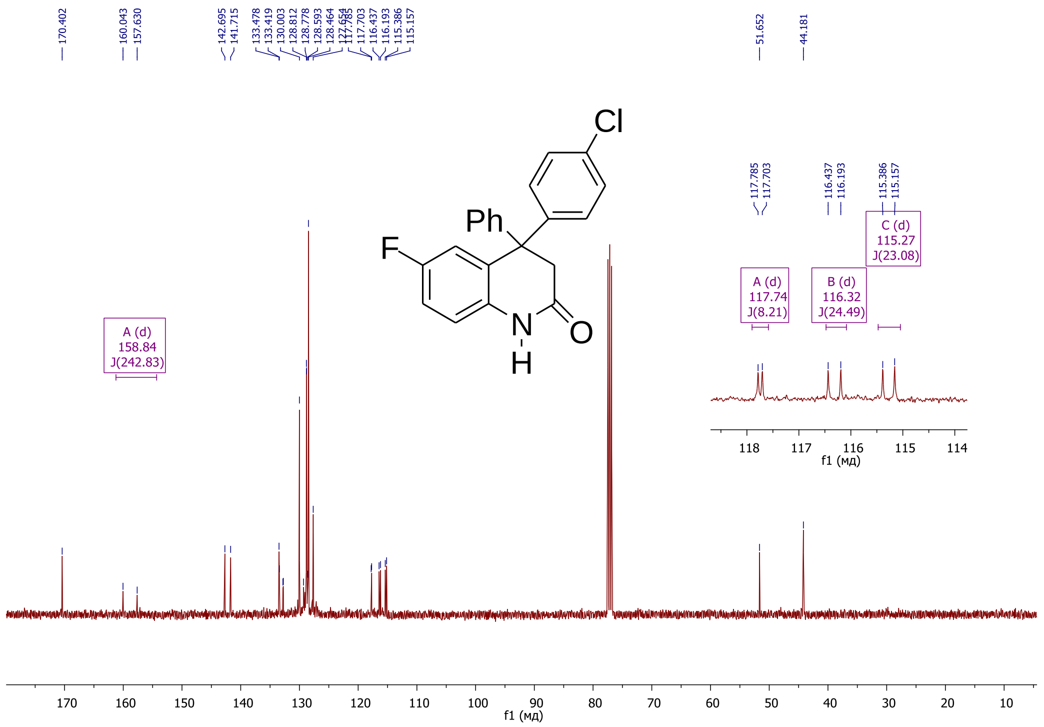

Figure S48. ^{13}C NMR spectrum of the compound **2s** (100 MHz, $CDCl_3$).

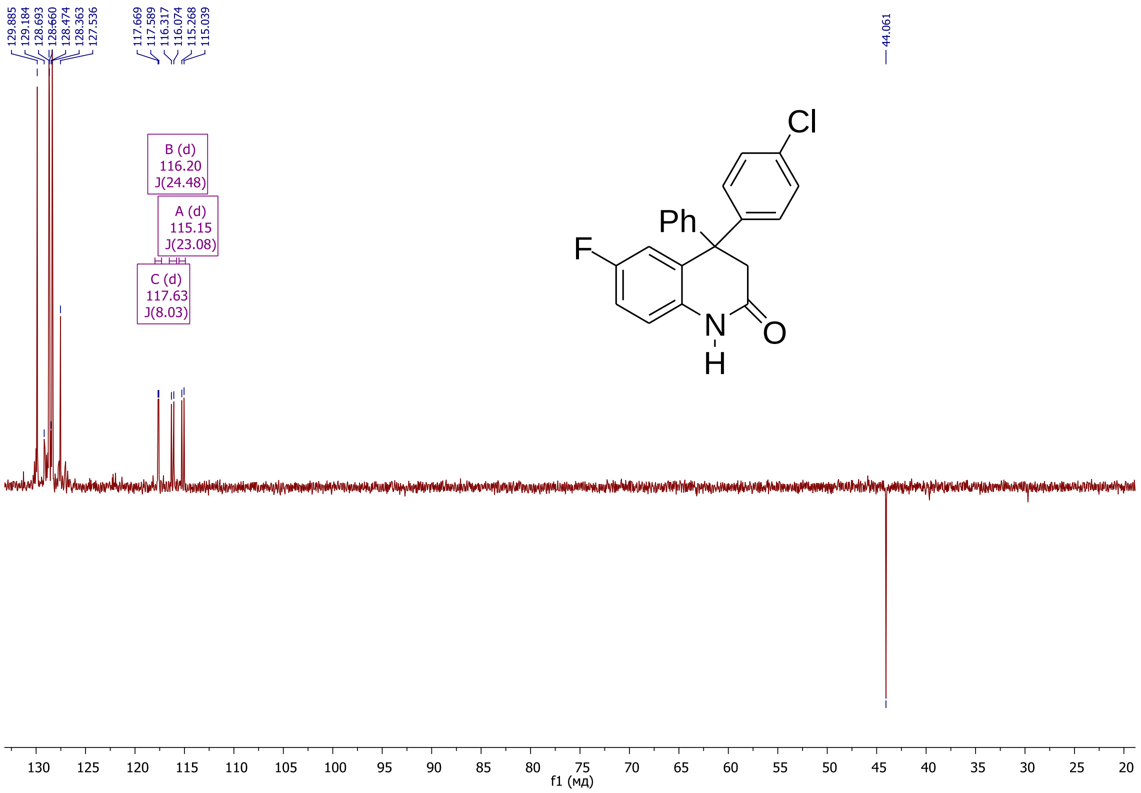

Figure S49. DEPT spectrum of the compound **2s** (100 MHz, $CDCl_3$).

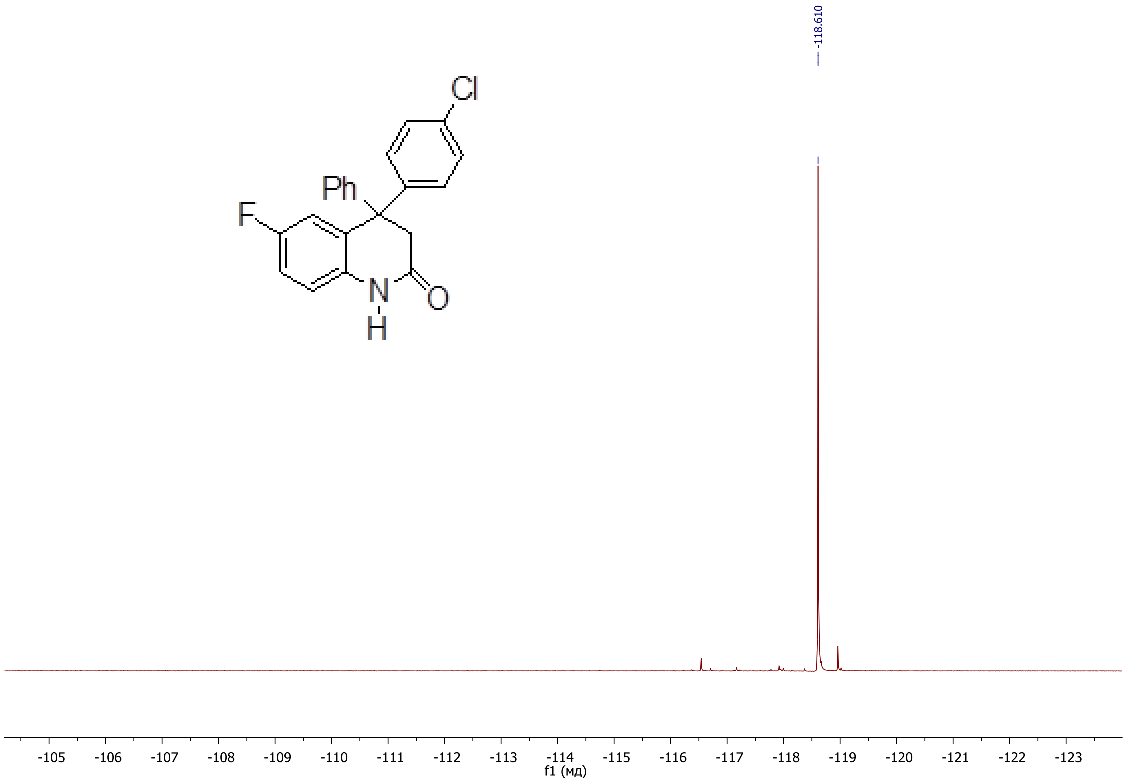

Figure S50. ^{19}F {^{1}H} NMR spectrum of the compound **2s** (376 MHz, $CDCl_3$).

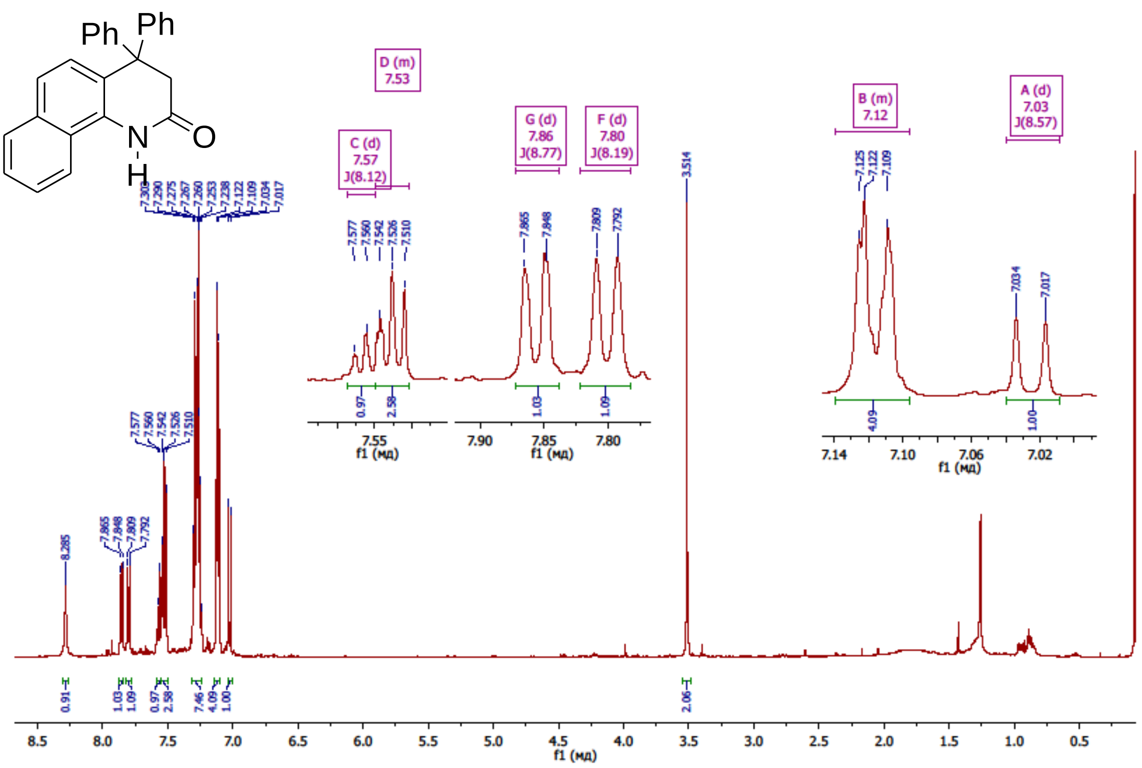

Figure S51. ^{1}H NMR spectrum of the compound **2t** (400 MHz, $CDCl_3$).

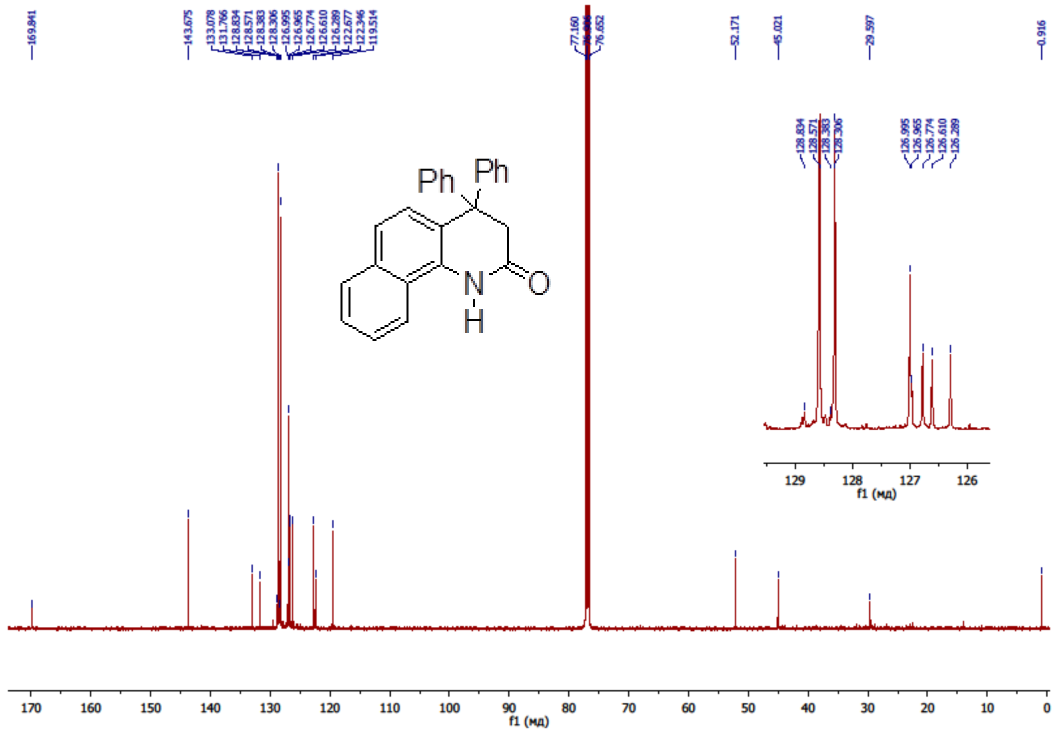

Figure S52. ^{13}C NMR spectrum of the compound **2t** (100 MHz, $CDCl_3$).

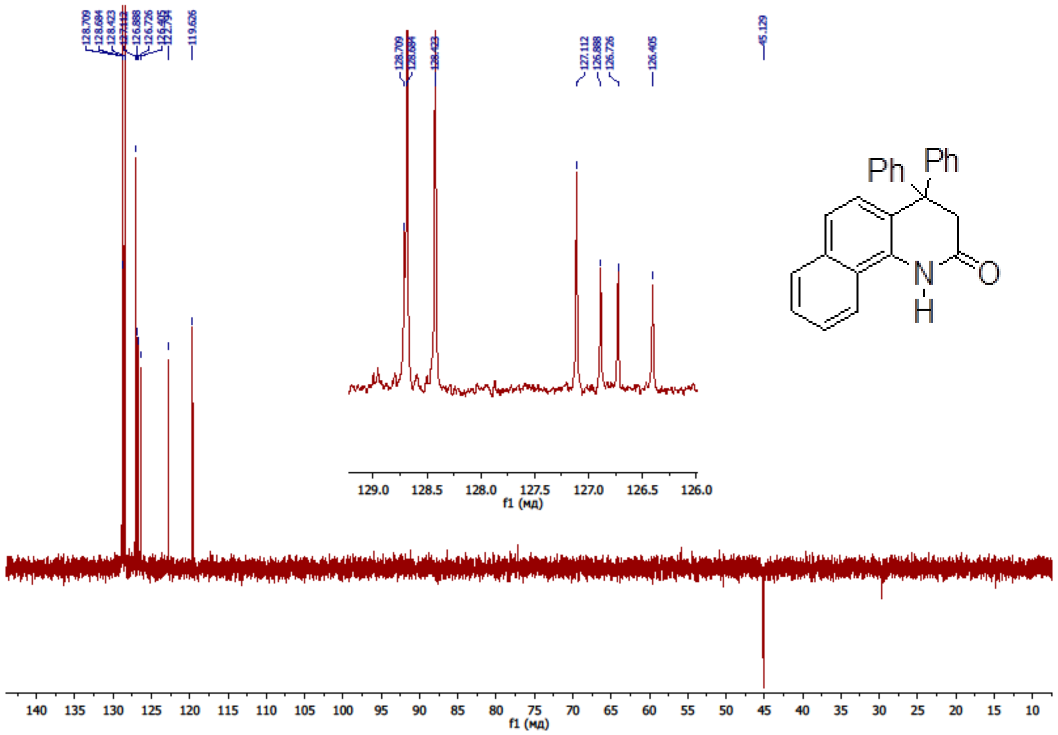

Figure S53. DEPT spectrum of the compound **2t** (100 MHz, $CDCl_3$).

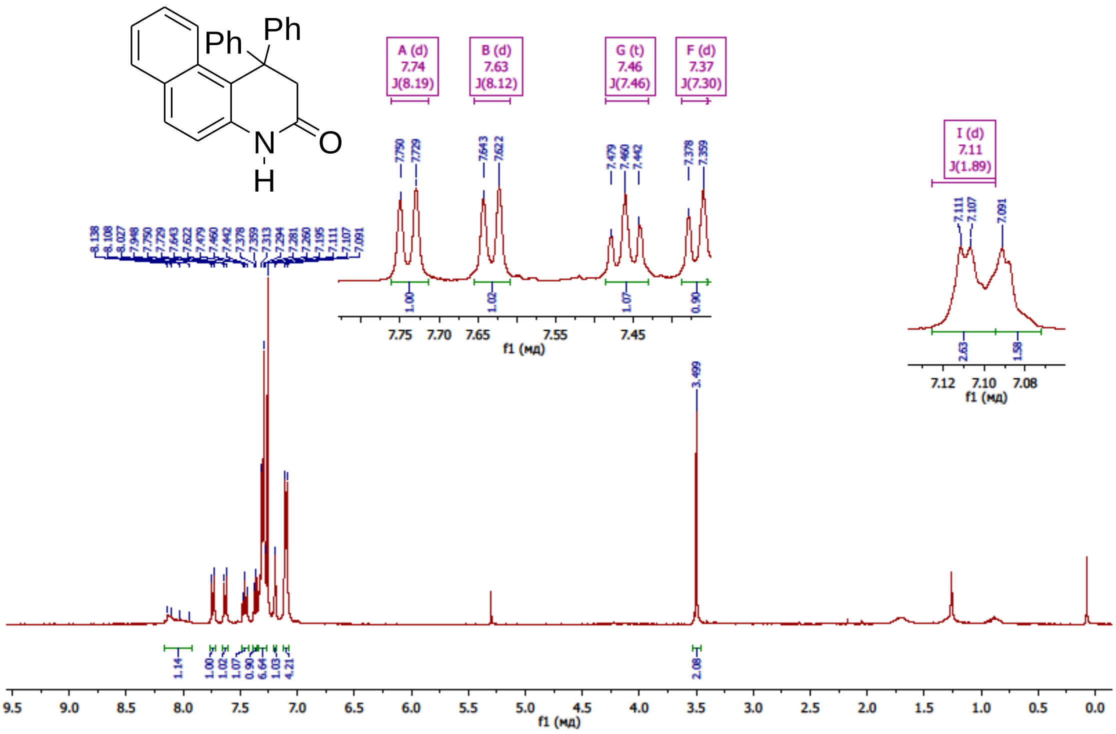

Figure S54. ^{1}H NMR spectrum of the compound **2u** (400 MHz, $CDCl_3$).

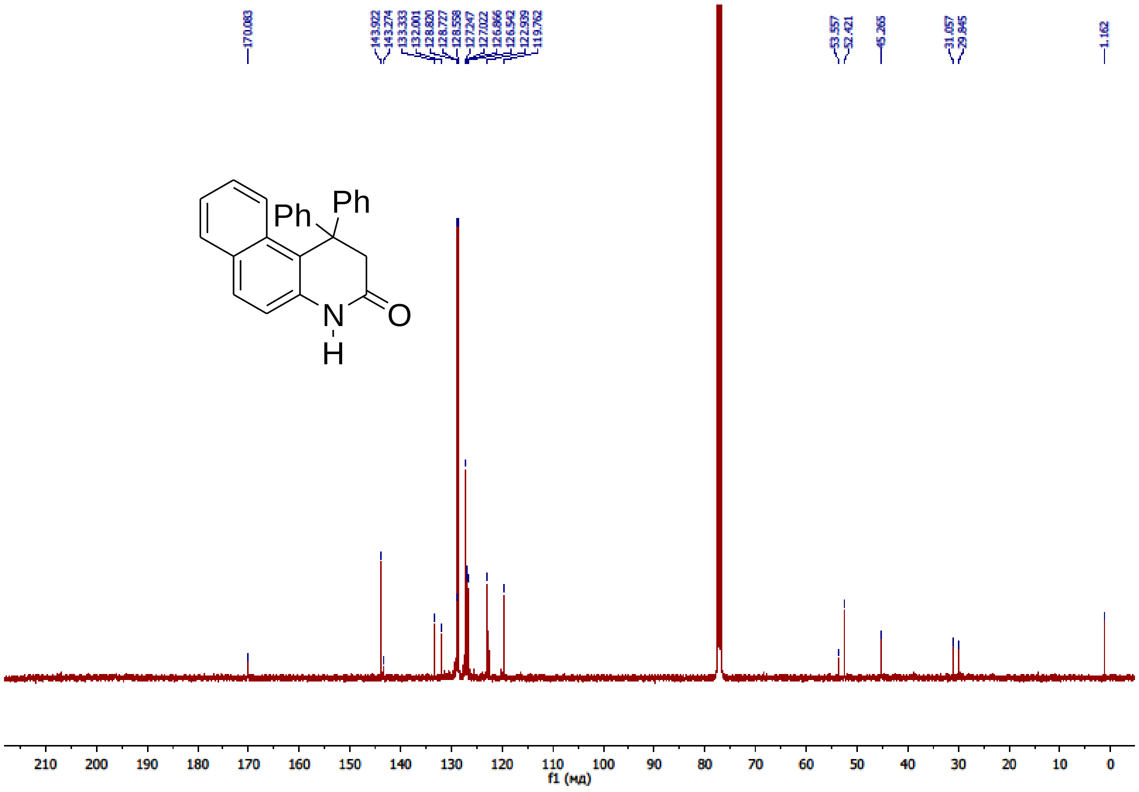

Figure S55. ^{13}C NMR spectrum of the compound **2u** (100 MHz, $CDCl_3$).

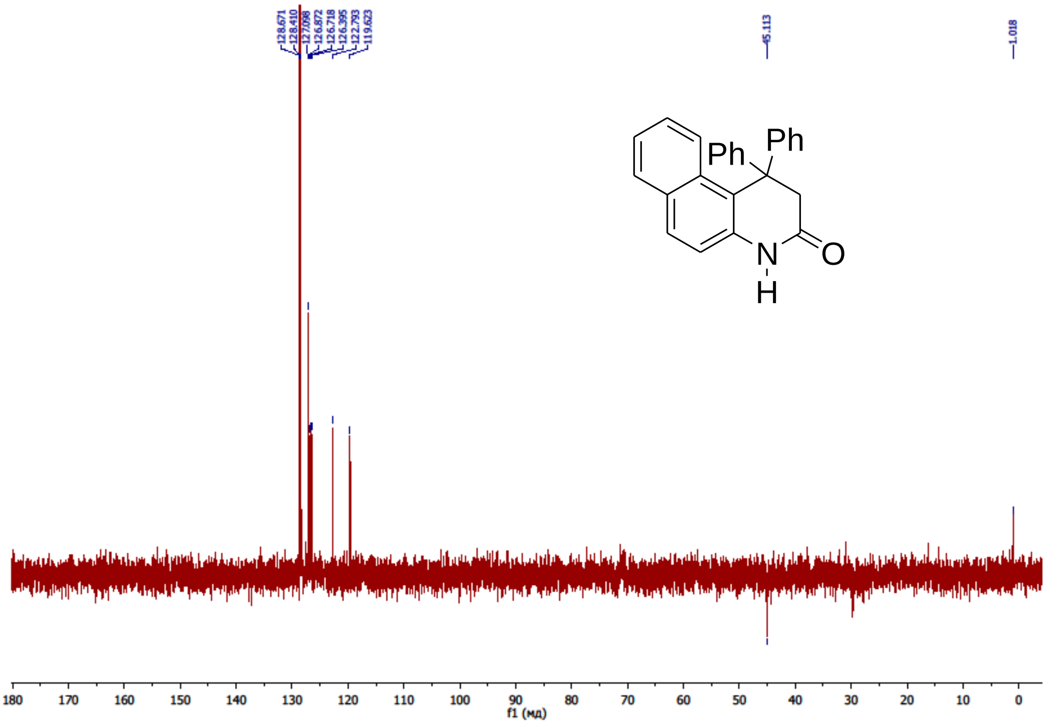

Figure S56. DEPT spectrum of the compound **2u** (100 MHz, $CDCl_3$).

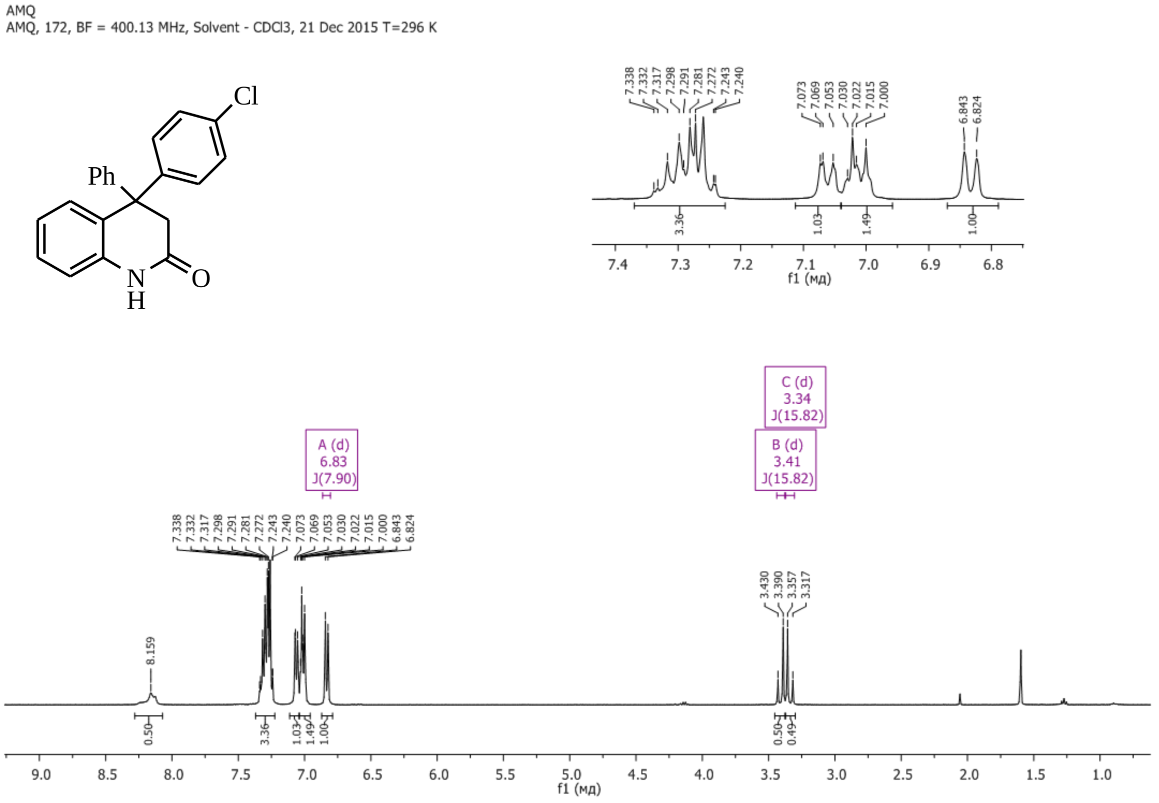

Figure S57. ^{1}H NMR spectrum of the compound **2v** (400 MHz, $CDCl_3$).

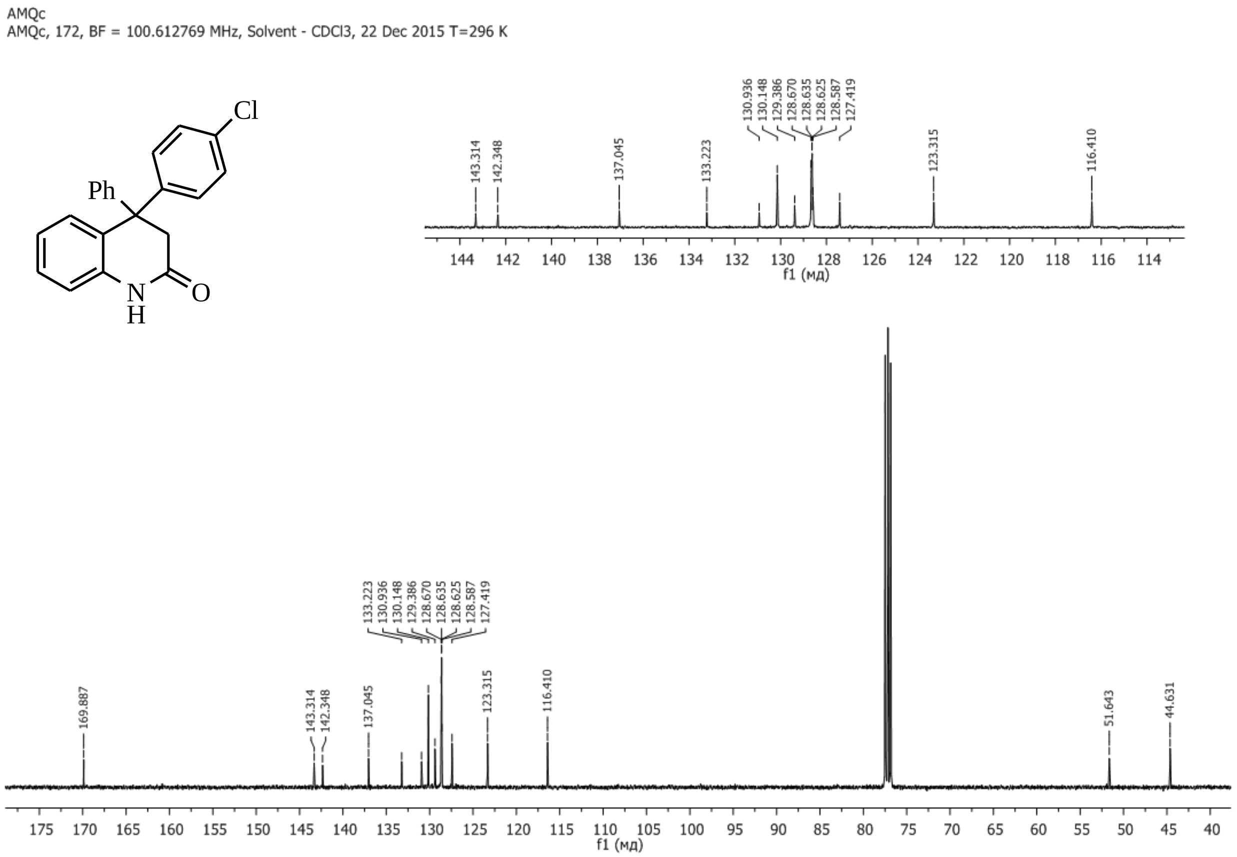

Figure S58. ^{13}C NMR spectrum of the compound **2v** (100 MHz, $CDCl_3$).

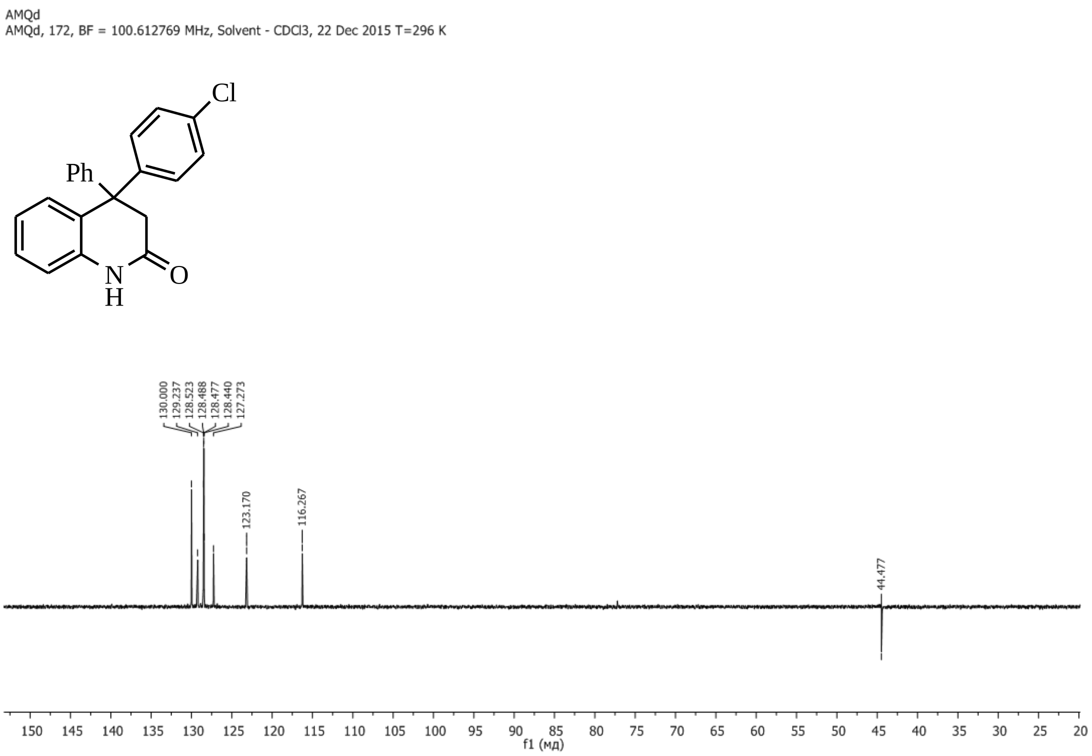

Figure S59. DEPT spectrum of the compound **2v** (100 MHz, $CDCl_3$).

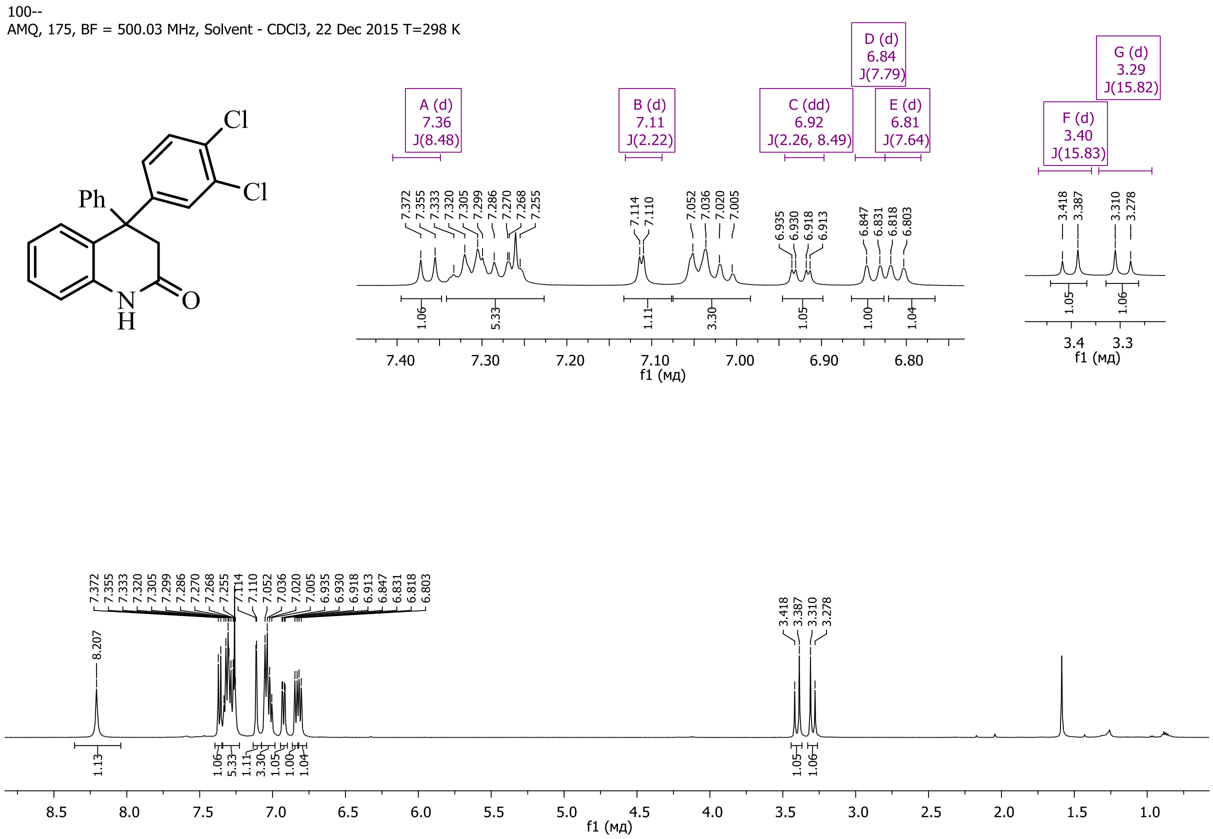

Figure S60. ^{1}H NMR spectrum of the compound **2x** (400 MHz, $CDCl_3$).

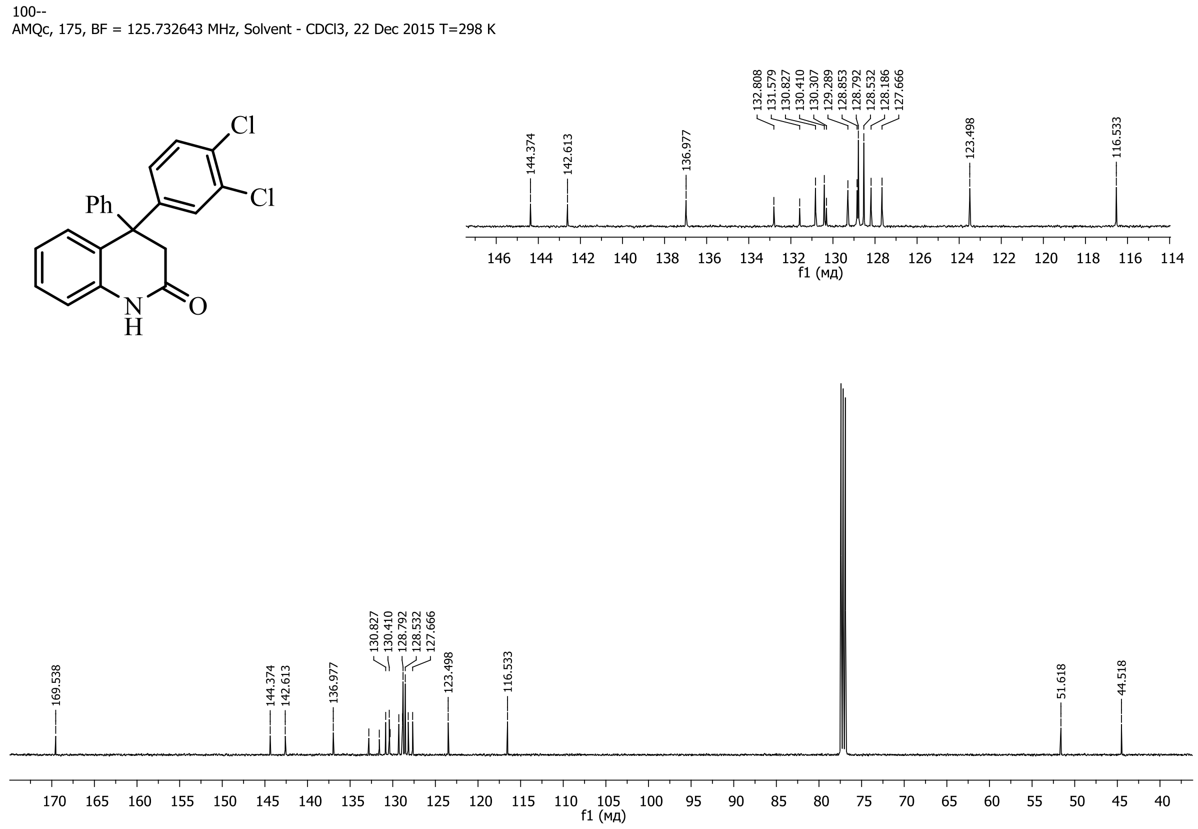

Figure S61. ^{13}C NMR spectrum of the compound **2x** (100 MHz, $CDCl_3$).

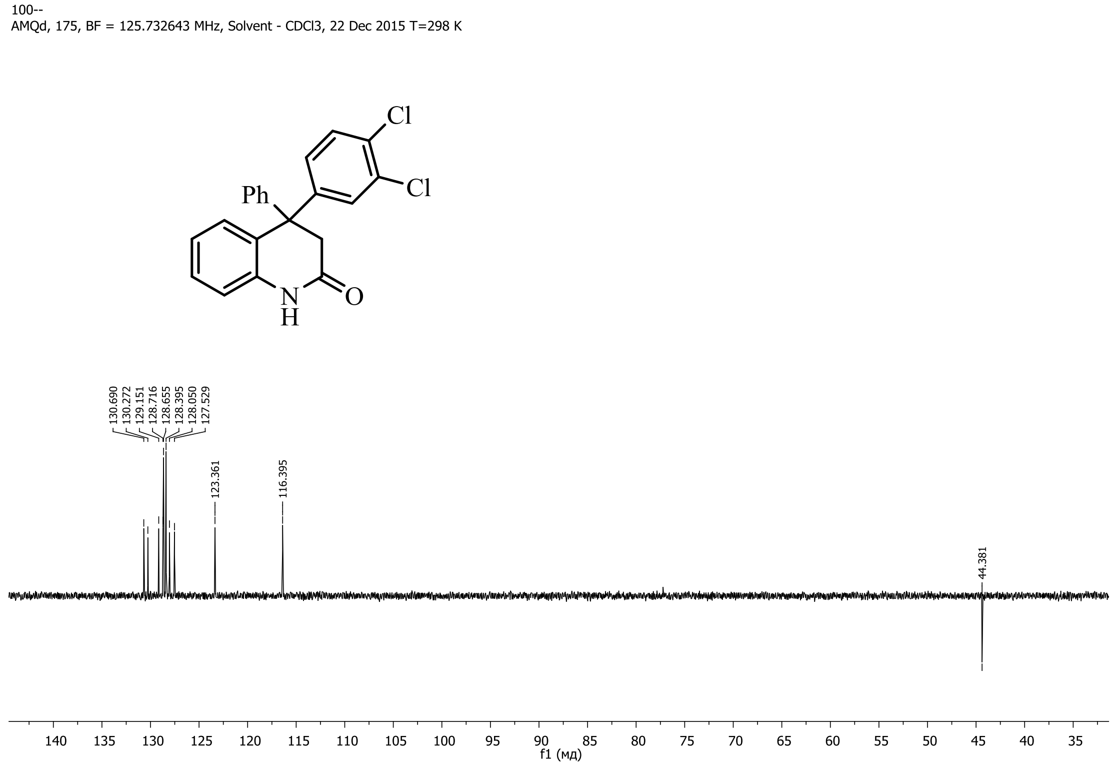

Figure S62. DEPT spectrum of the compound **2x** (100 MHz, $CDCl_3$).

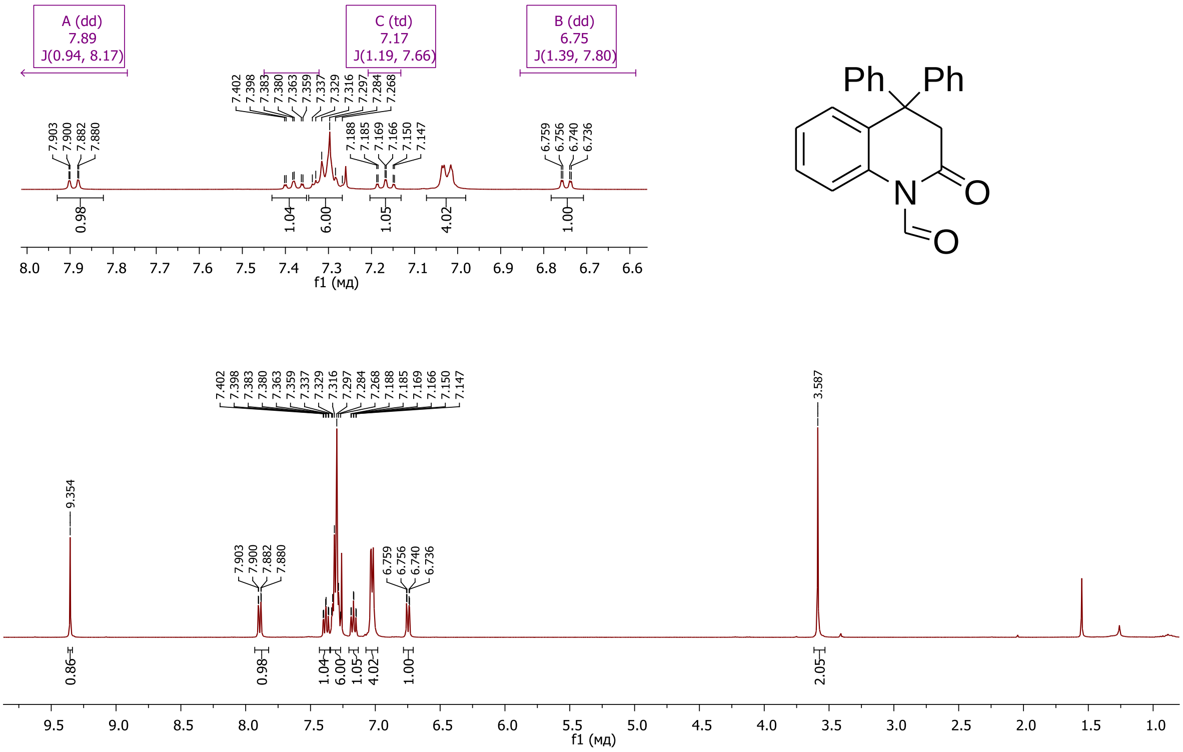

Figure S63. ^{1}H NMR spectrum of the compound **5a** (400 MHz, $CDCl_3$).

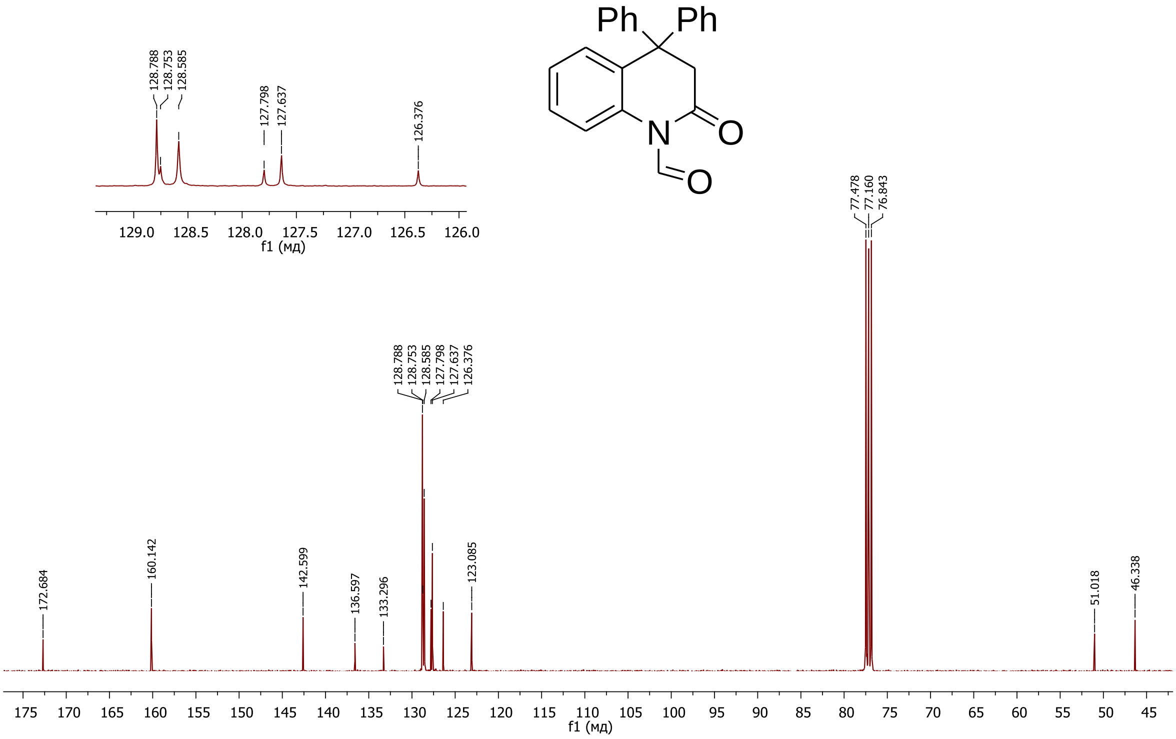

Figure S64. ^{13}C NMR spectrum of the compound **5a** (100 MHz, $CDCl_3$).

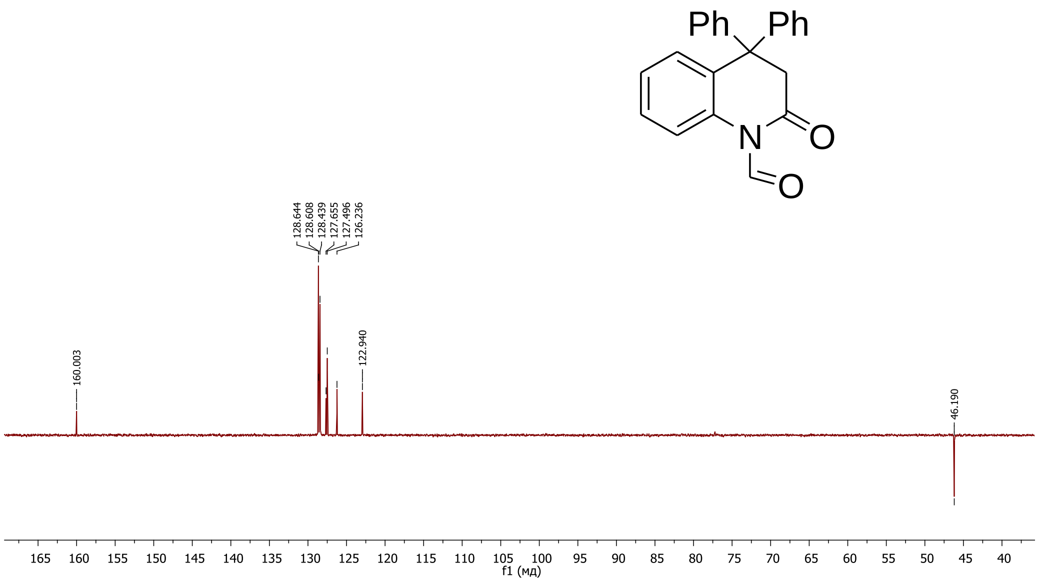

Figure S65. DEPT spectrum of the compound **5a** (100 MHz, $CDCl_3$).

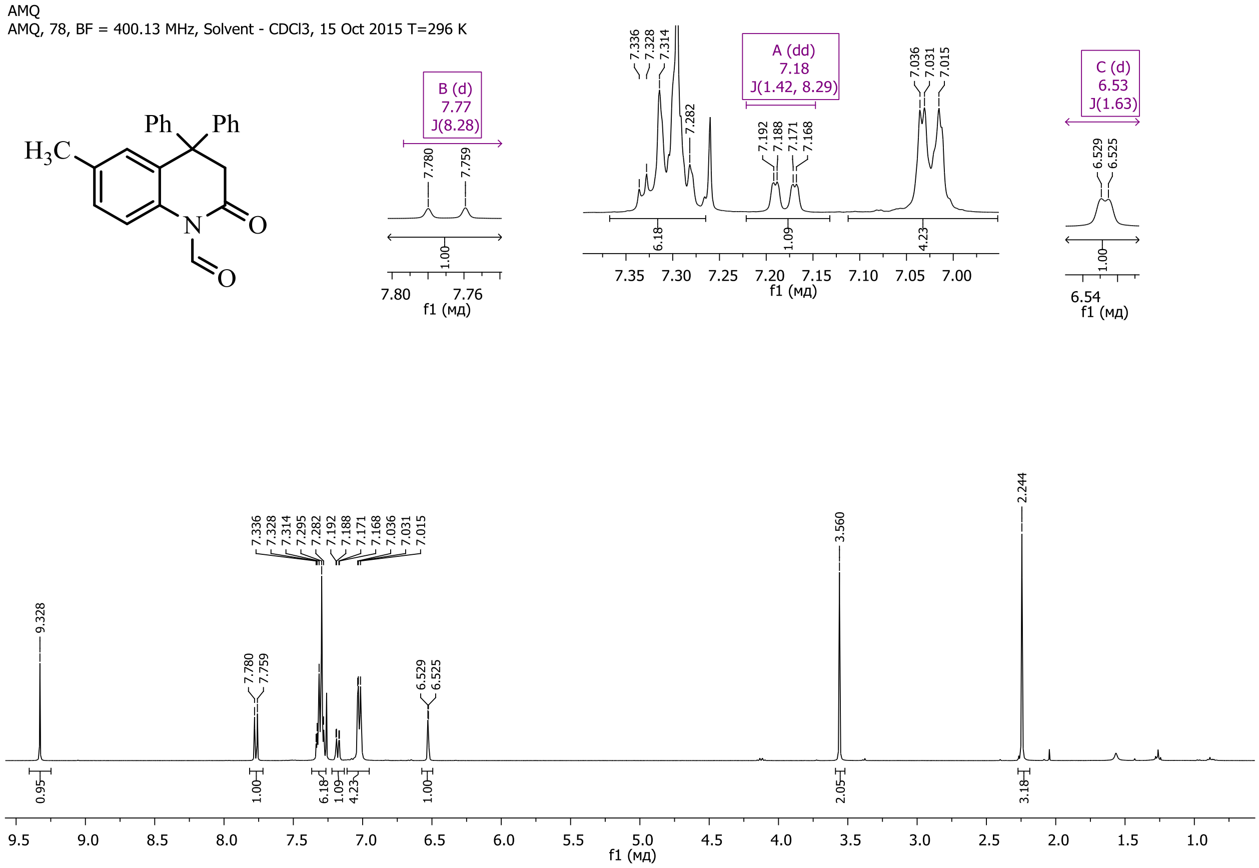

Figure S66. ^{1}H NMR spectrum of the compound **5b** (400 MHz, $CDCl_3$).

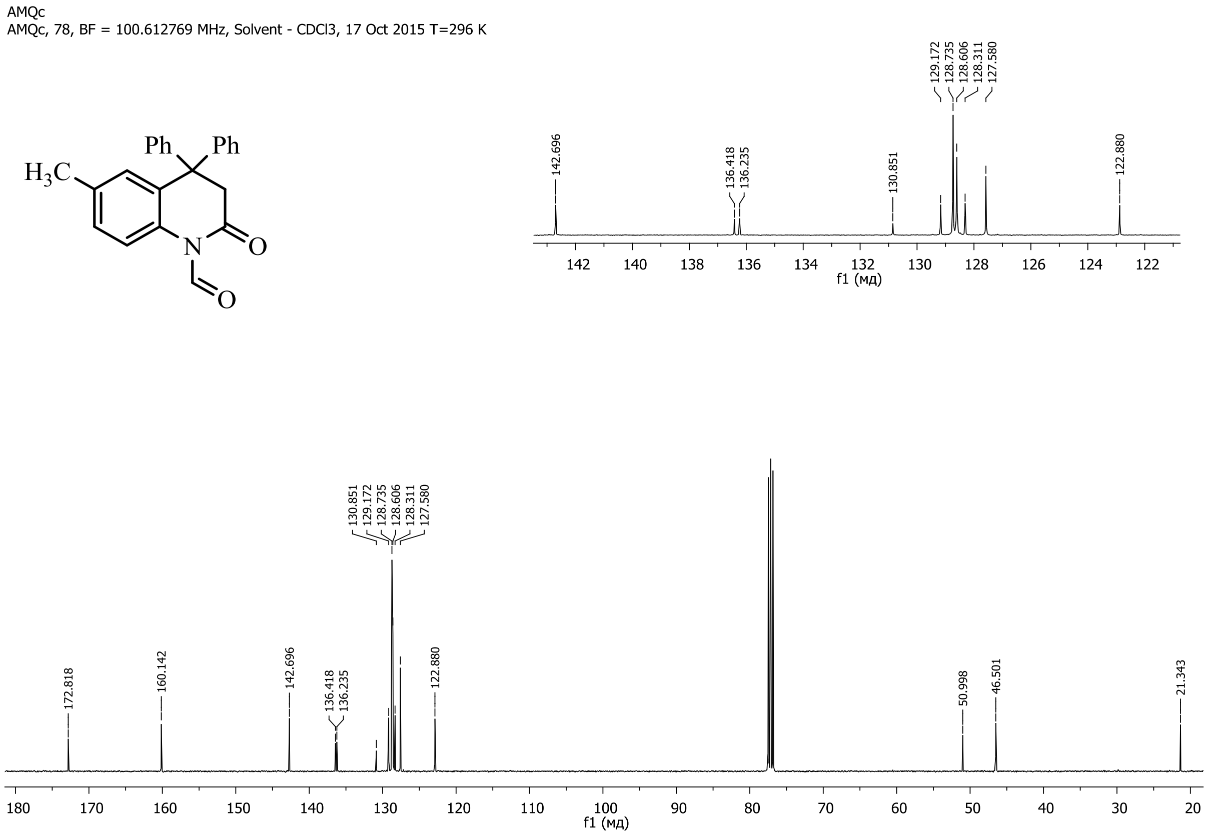

Figure S67. ^{13}C NMR spectrum of the compound **5b** (100 MHz, $CDCl_3$).

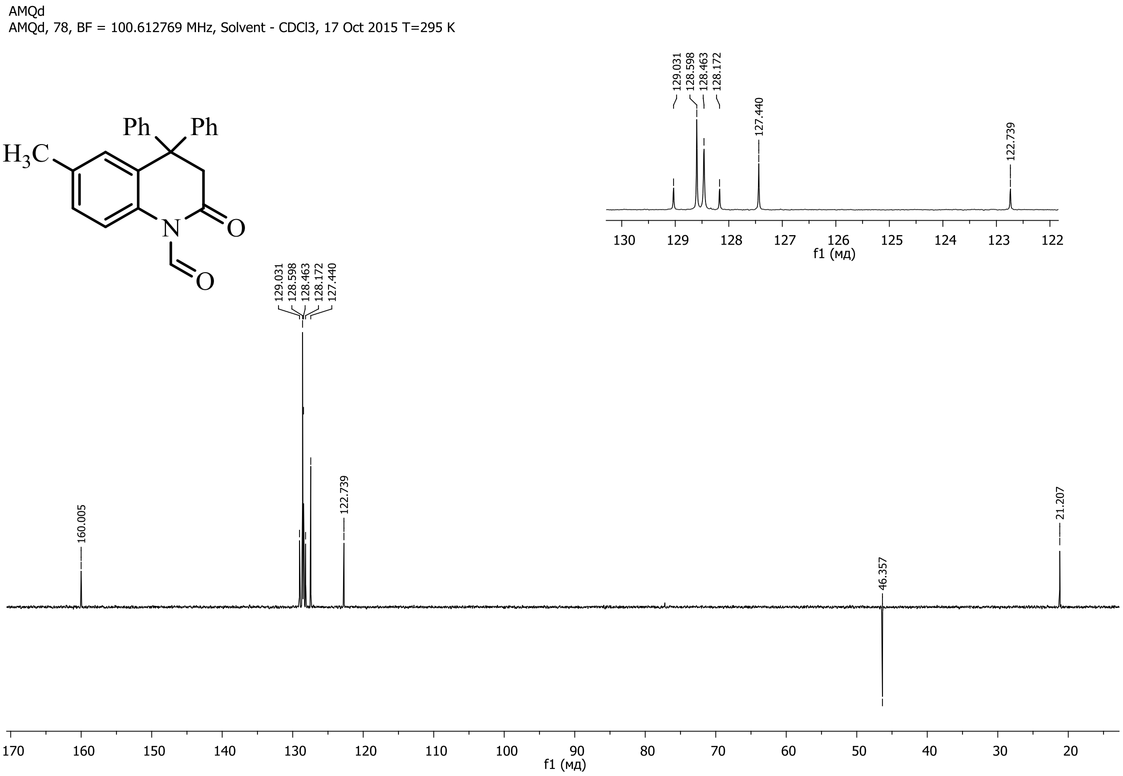

Figure S68. DEPT spectrum of the compound **5b** (100 MHz, $CDCl_3$).

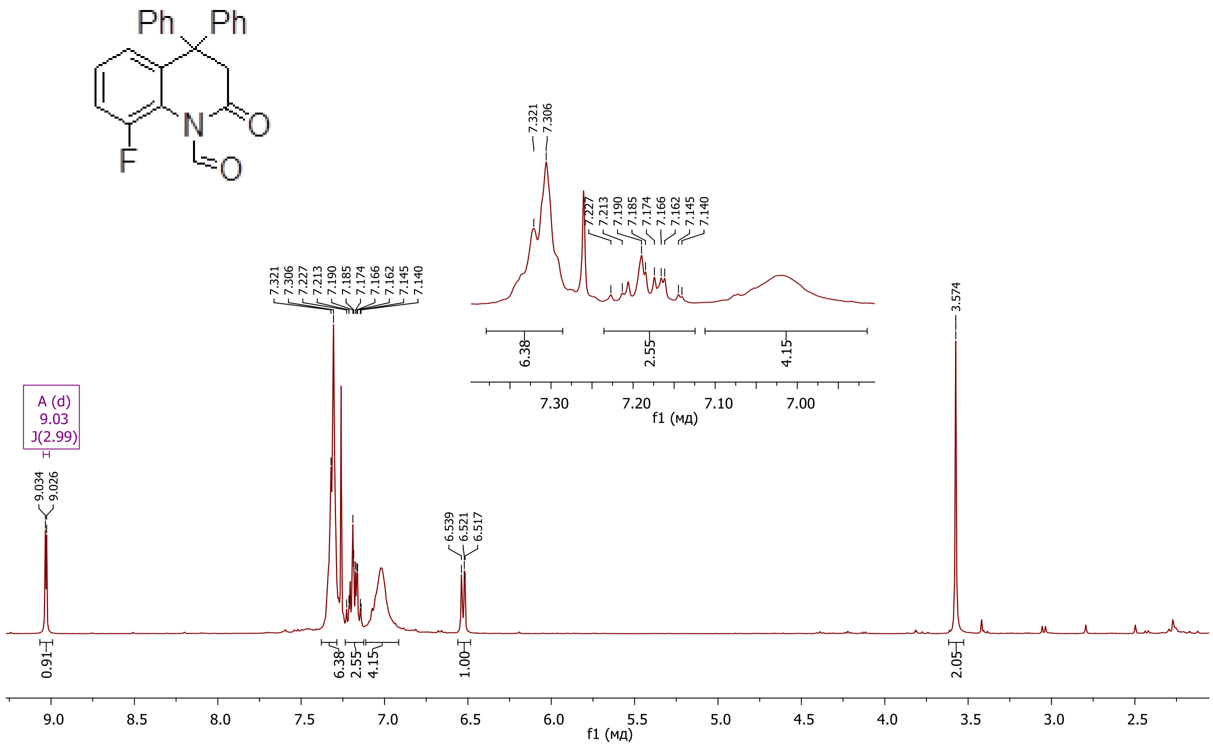

Figure S69. ^{1}H NMR spectrum of the compound **5c** (400 MHz, $CDCl_3$).

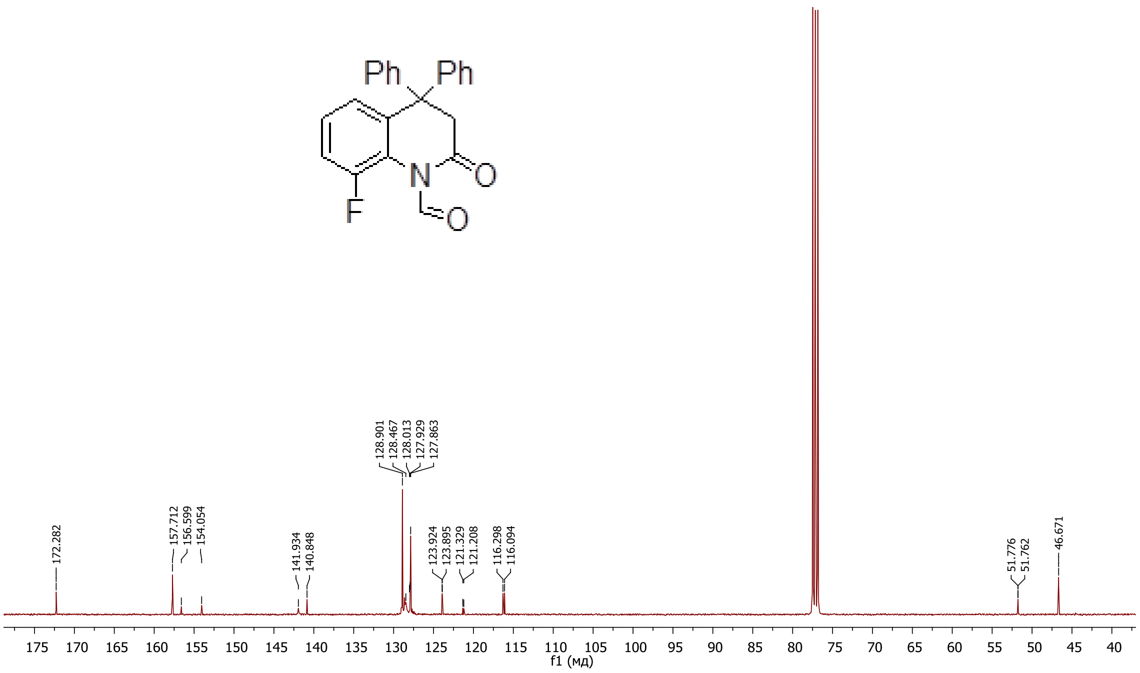

Figure S70. ^{13}C NMR spectrum of the compound **5c** (100 MHz, $CDCl_3$).

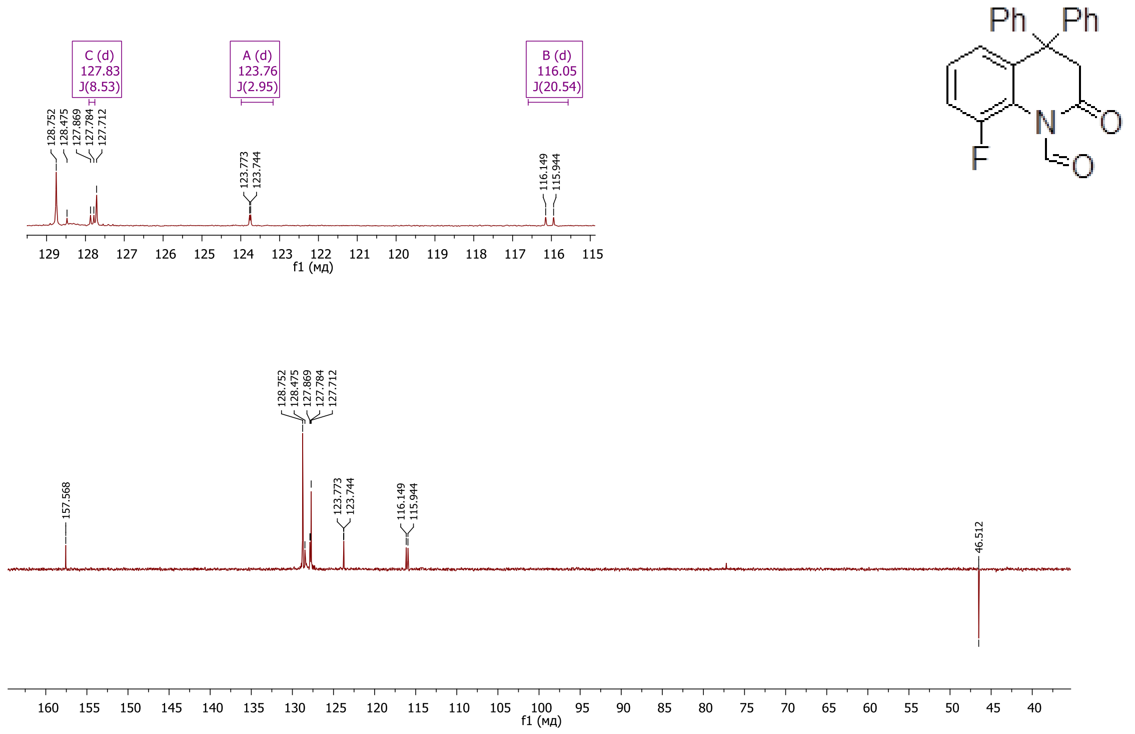

Figure S71. DEPT spectrum of the compound **5c** (100 MHz, $CDCl_3$).

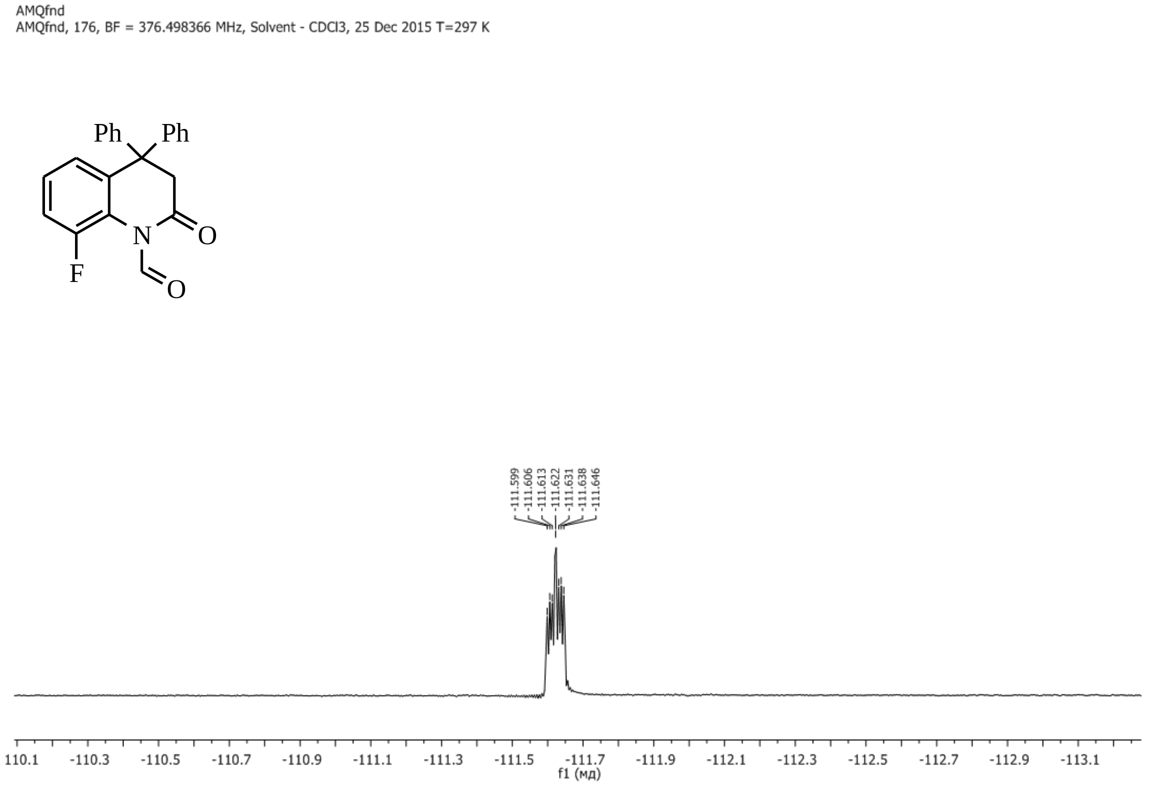

Figure S72. ^{19}F NMR spectrum of the compound **5c** (376 MHz, $CDCl_3$).

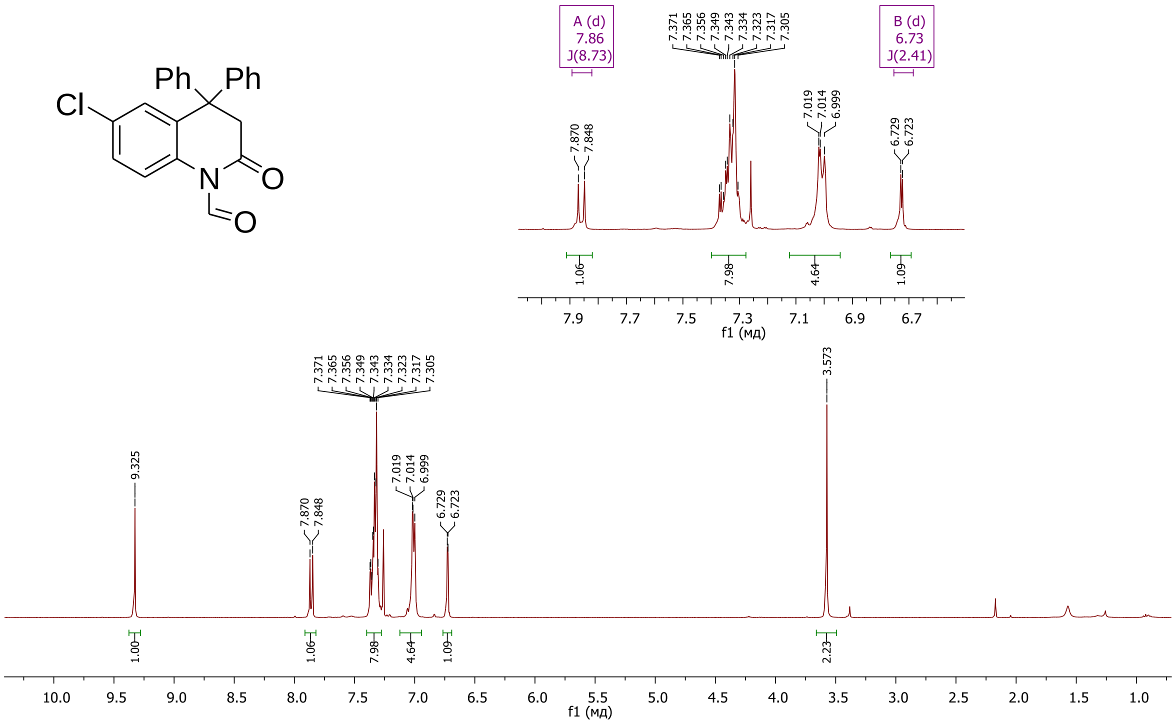

Figure S73. ^{1}H NMR spectrum of the compound **5d** (400 MHz, $CDCl_3$).

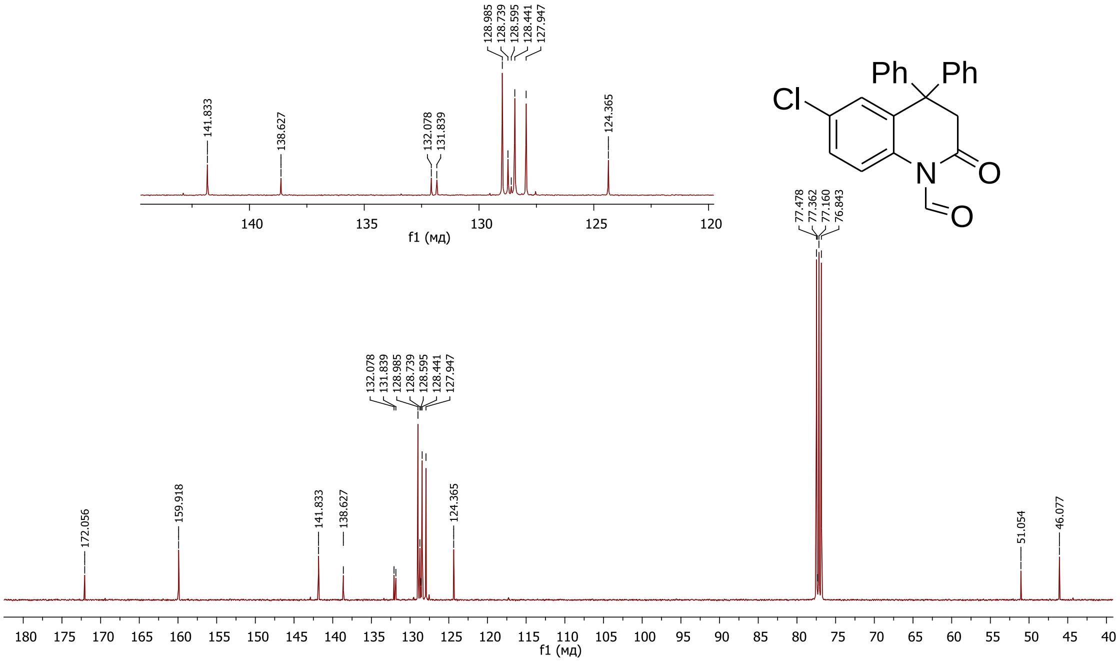

Figure S74. ^{13}C NMR spectrum of the compound **5d** (100 MHz, $CDCl_3$).

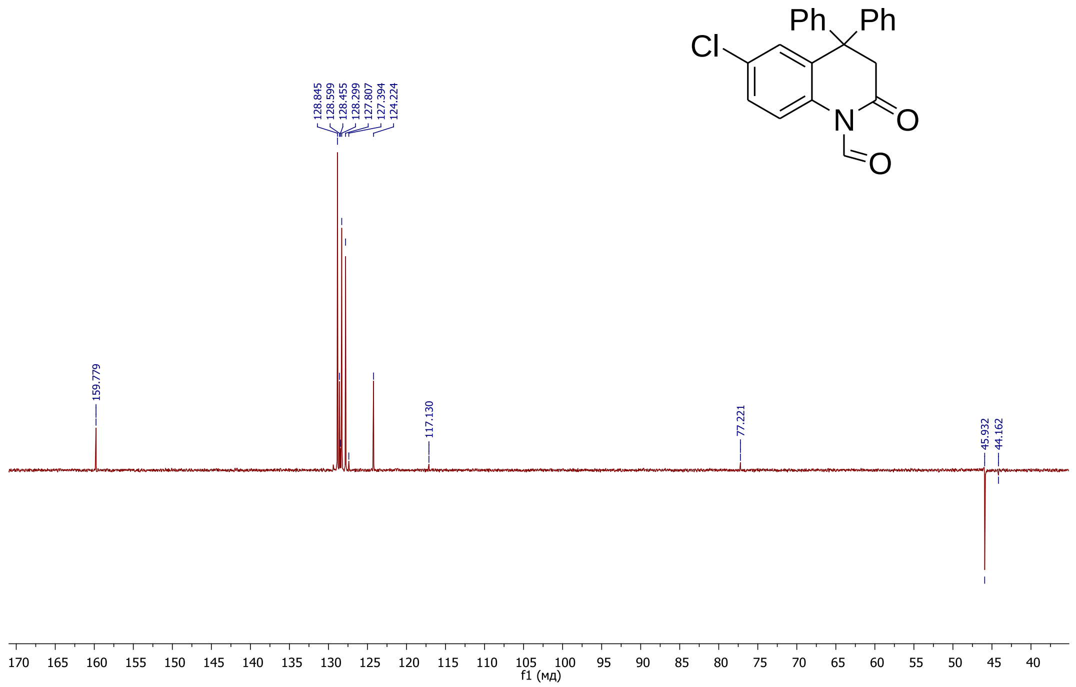

Figure S75. DEPT spectrum of the compound **5d** (100 MHz, $CDCl_3$).

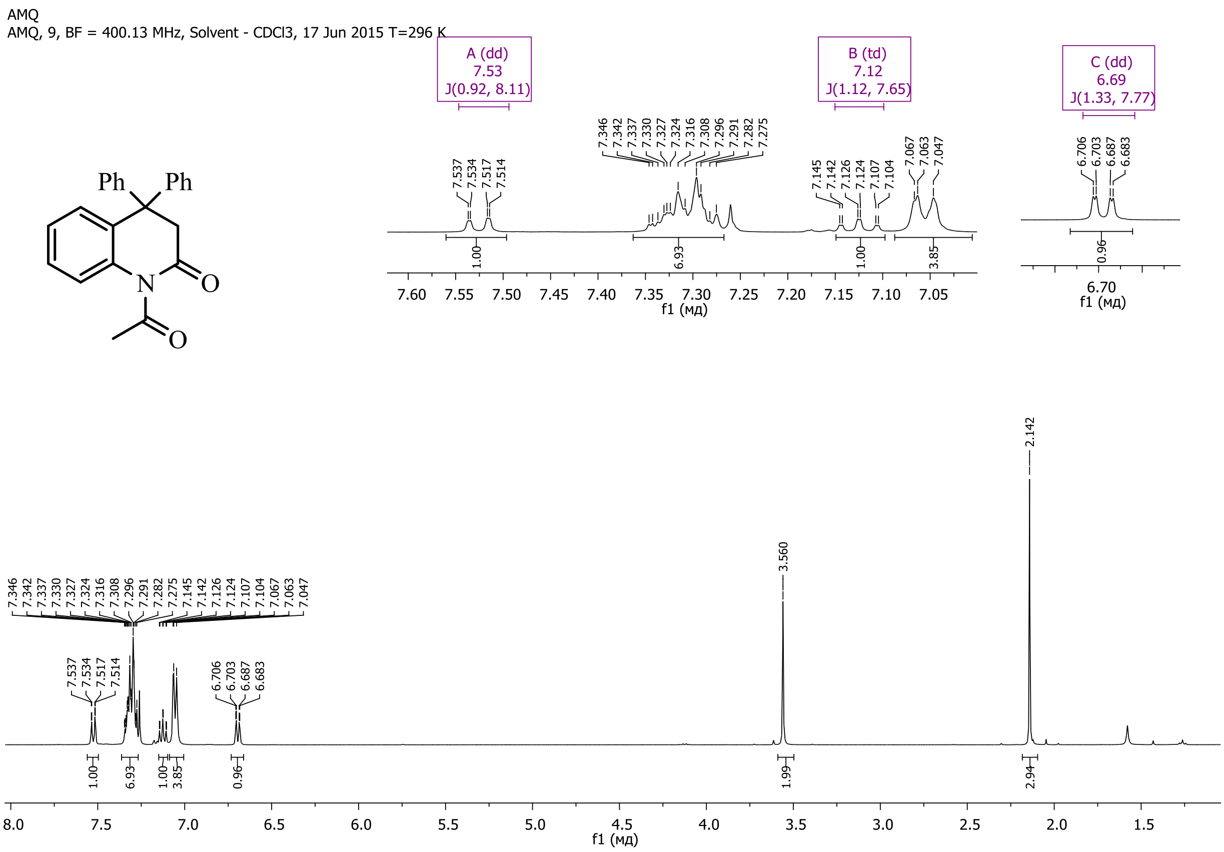

Figure S76. ^{1}H NMR spectrum of the compound **6a** (400 MHz, $CDCl_3$).

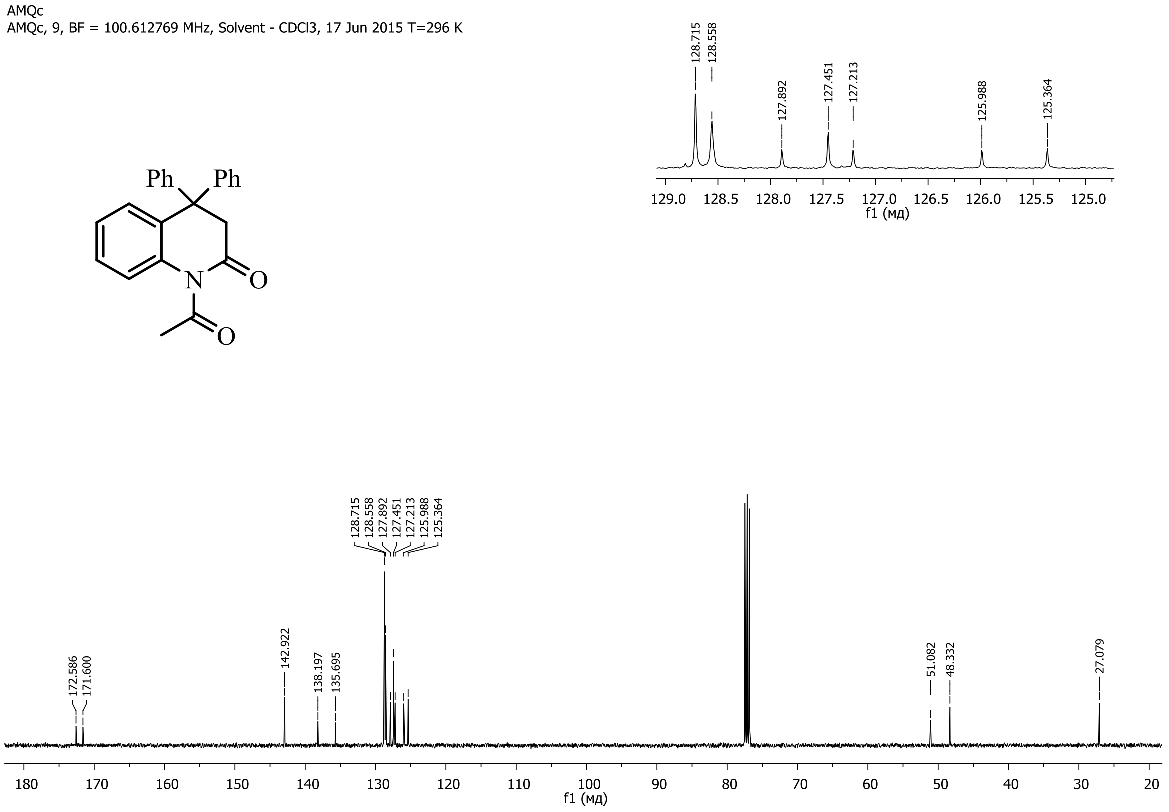

Figure S77. ^{13}C NMR spectrum of the compound **6a** (100 MHz, $CDCl_3$).

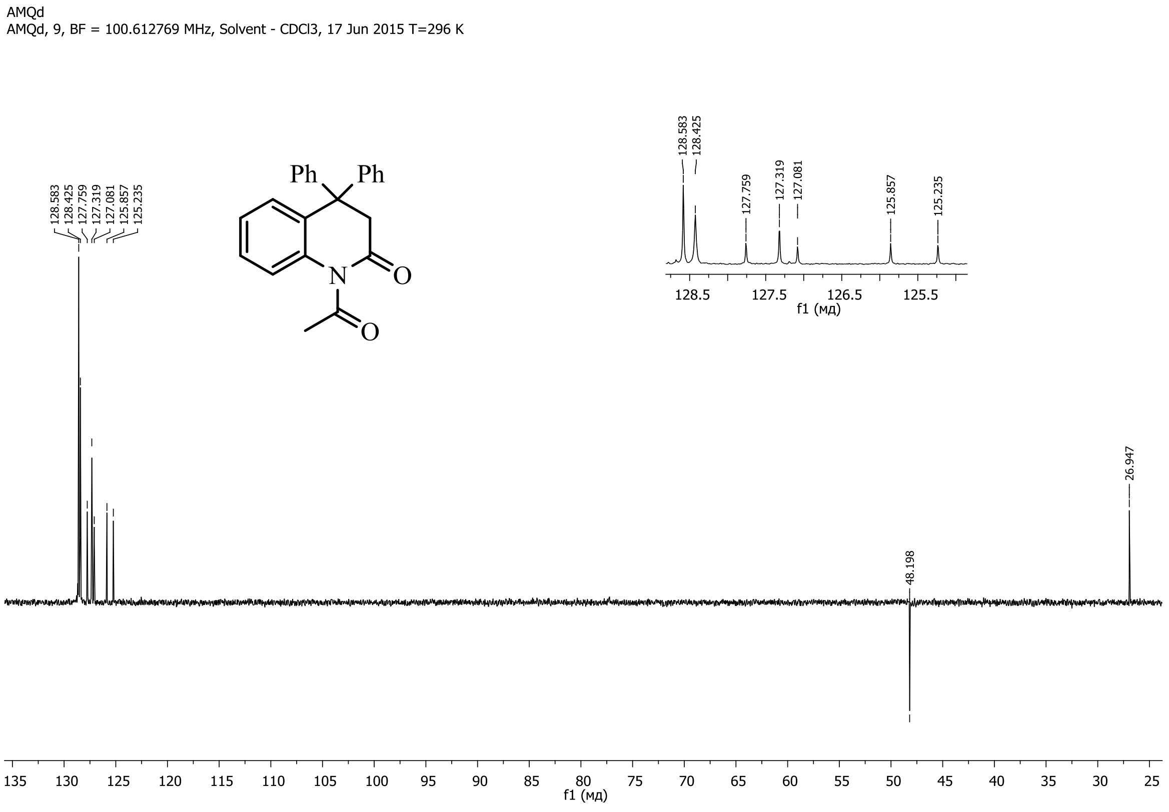

Figure S78. DEPT spectrum of the compound **6a** (100 MHz, $CDCl_3$).

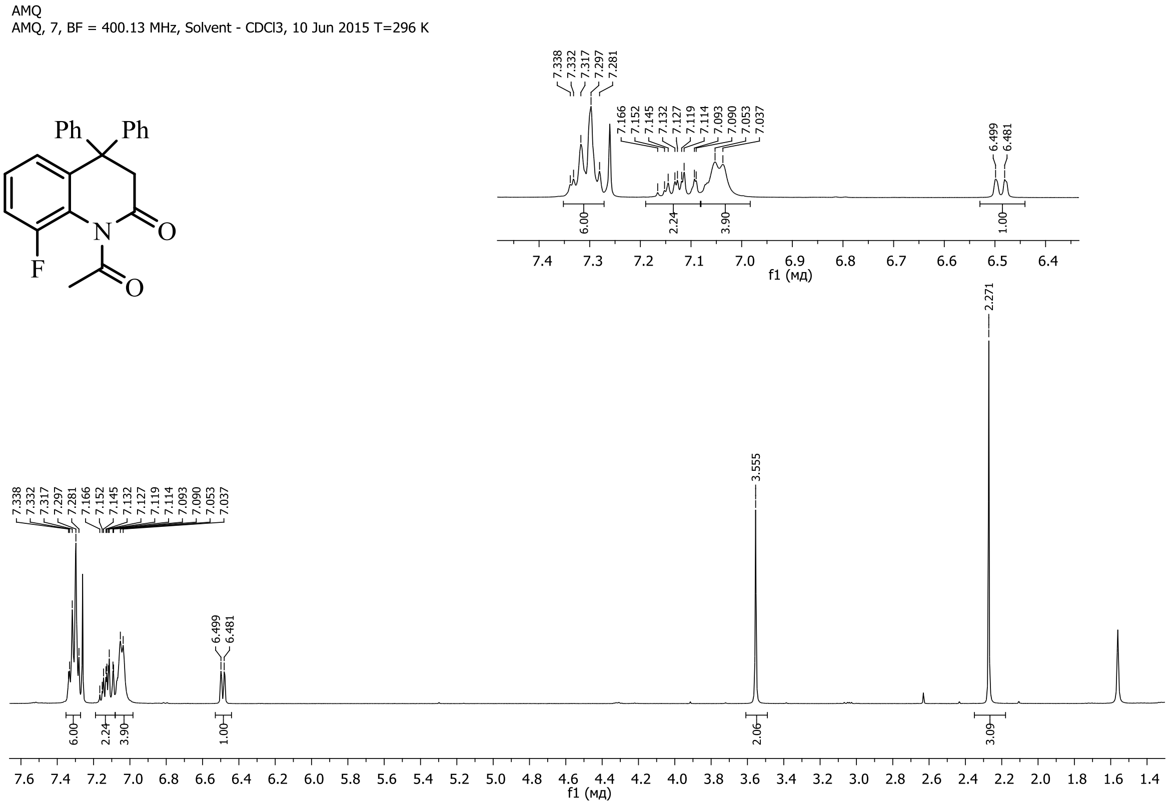

Figure S79. ^{1}H NMR spectrum of the compound **6b** (400 MHz, $CDCl_3$).

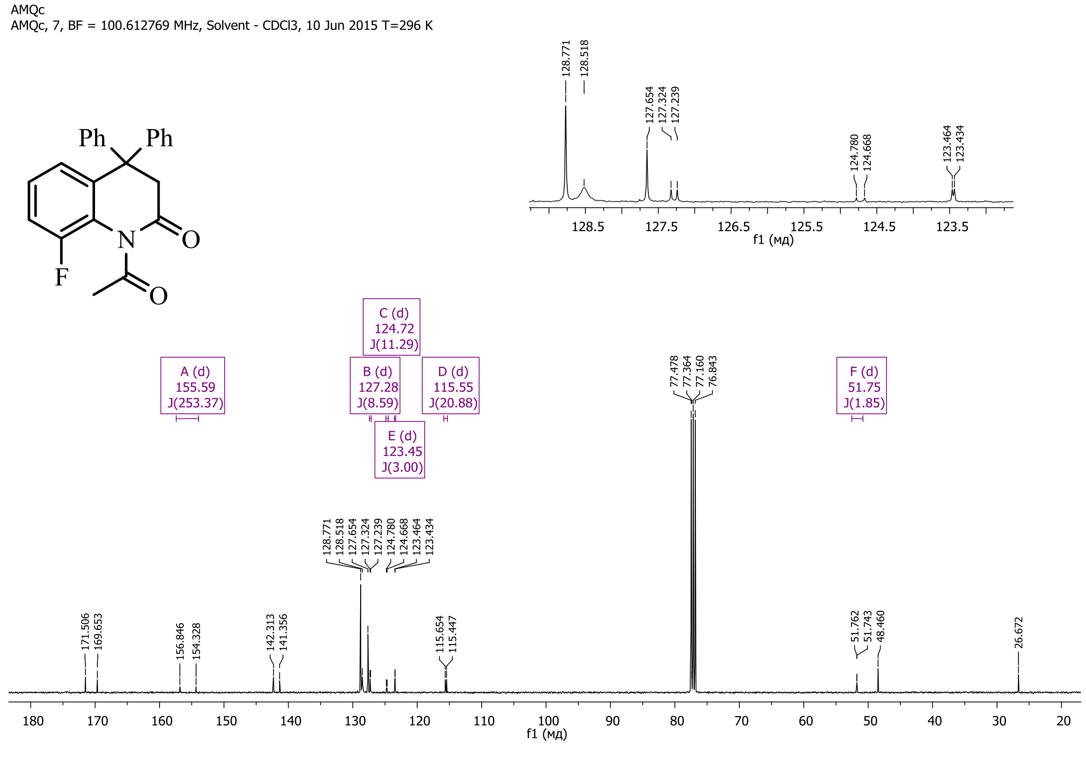

Figure S80. ^{13}C NMR spectrum of the compound **6b** (100 MHz, $CDCl_3$).

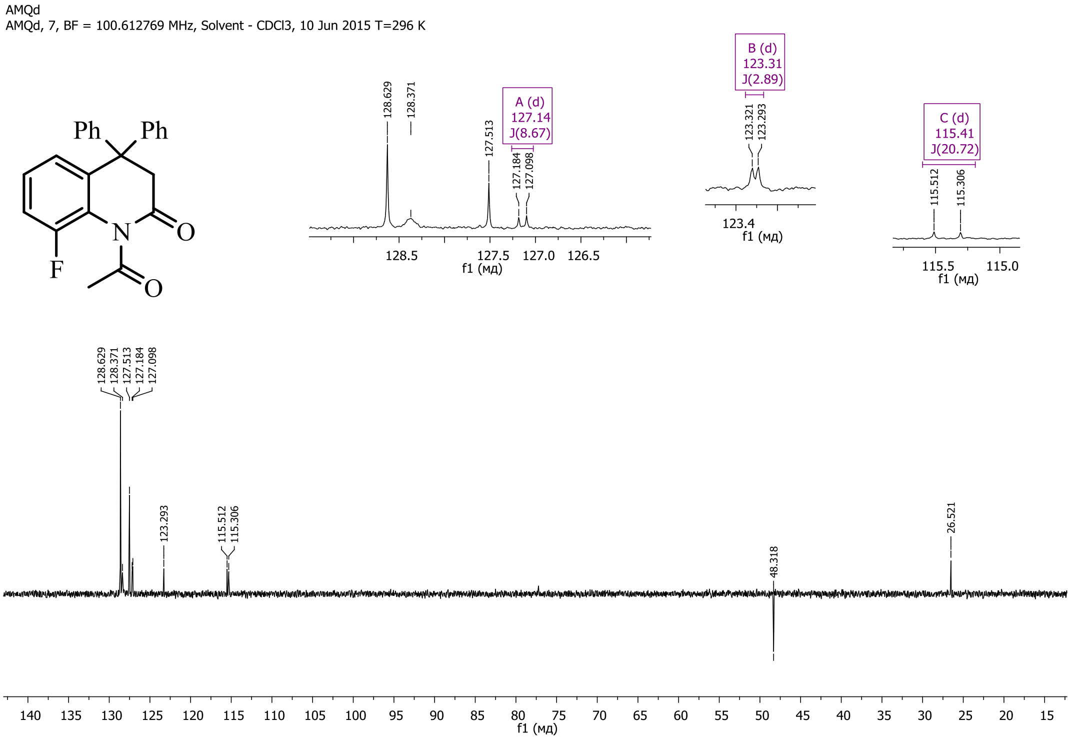

Figure S81. DEPT spectrum of the compound **6b** (100 MHz, $CDCl_3$).

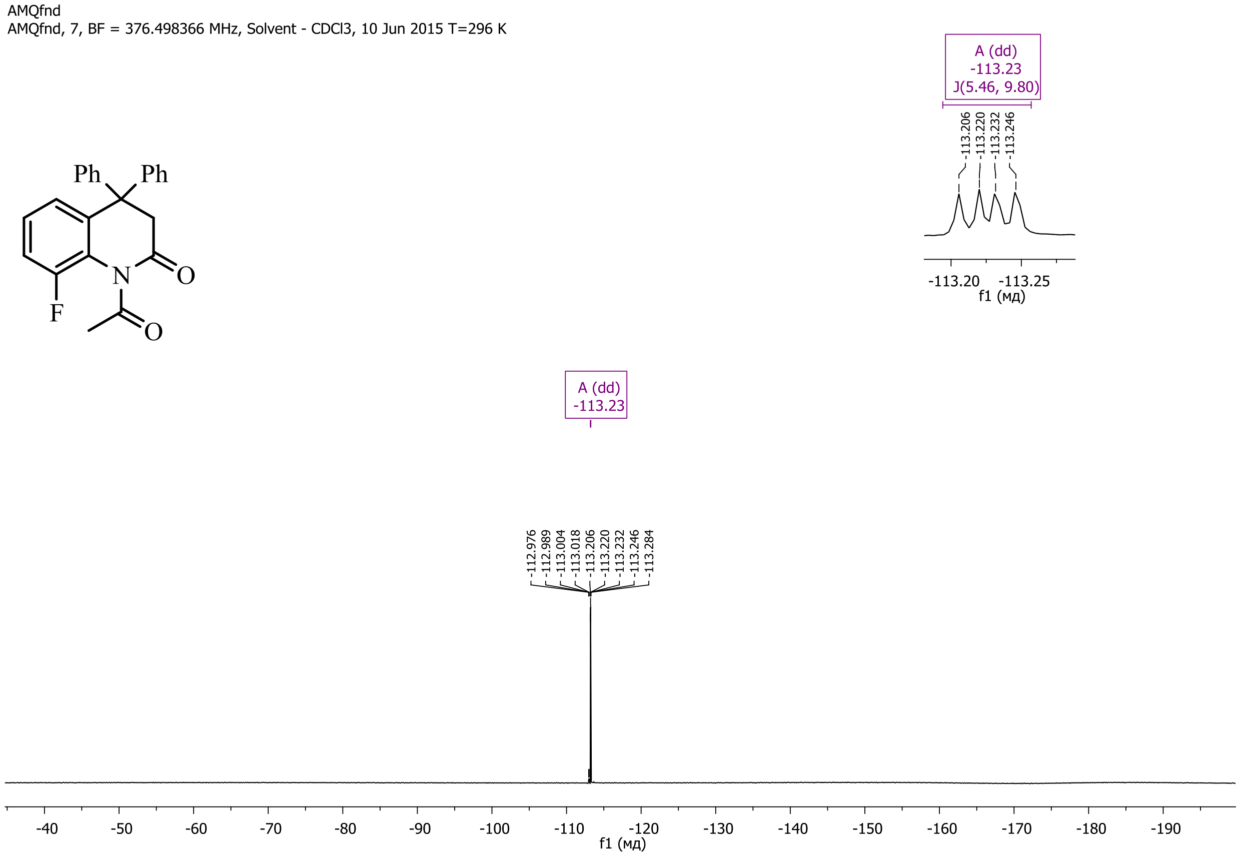

Figure S82. ^{19}F NMR spectrum of the compound **6b** (376 MHz, $CDCl_3$).

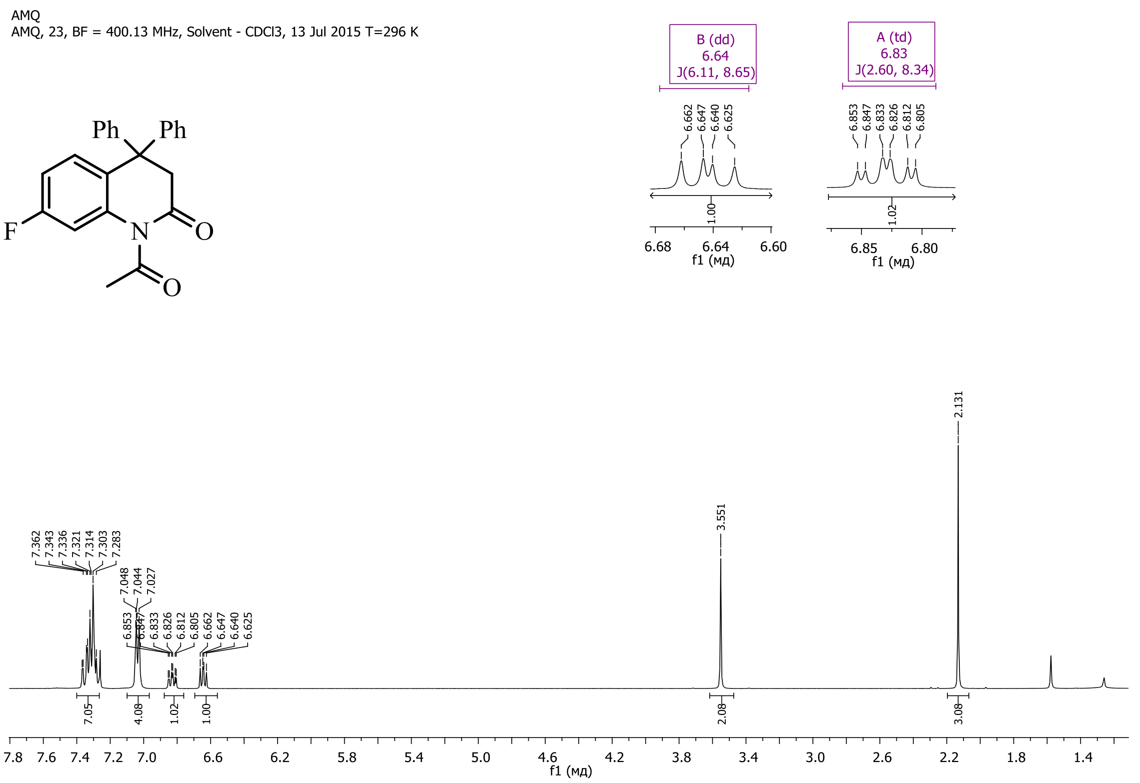

Figure S83. ^{1}H NMR spectrum of the compound **6c** (400 MHz, $CDCl_3$).

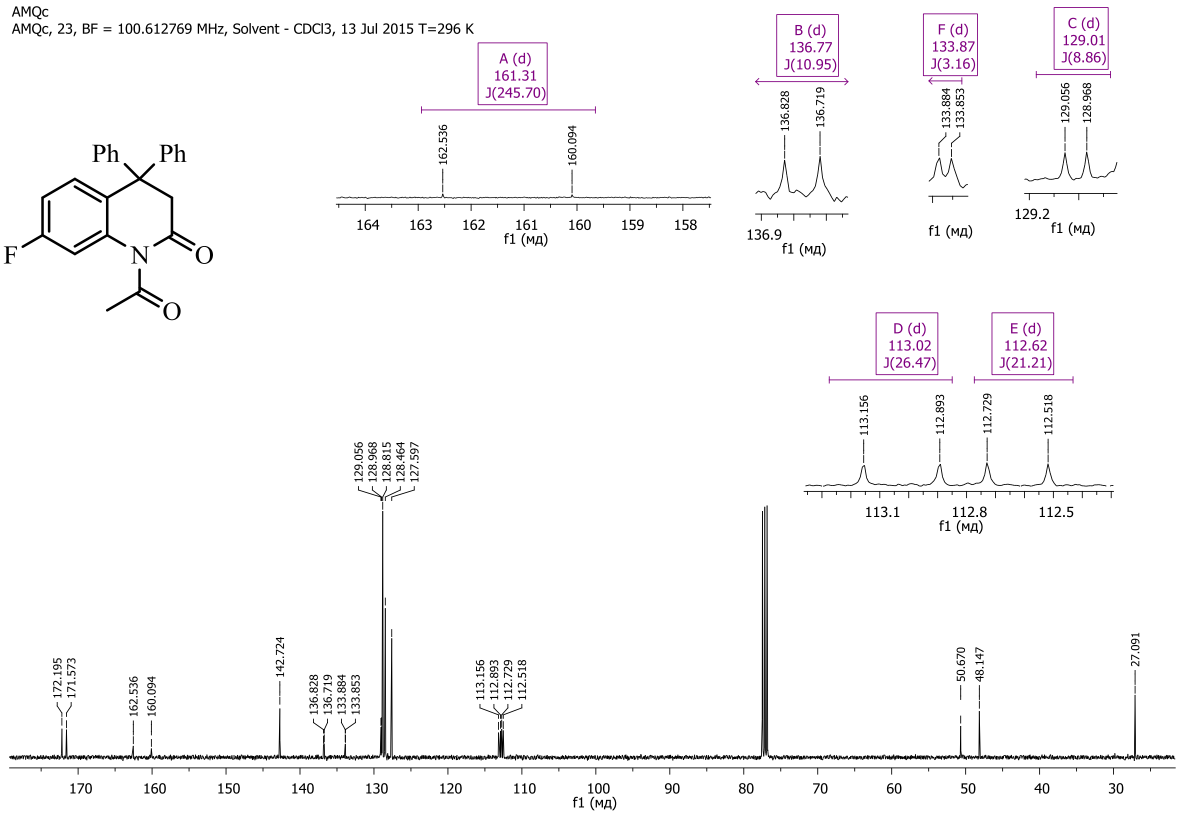

Figure S84. ^{13}C NMR spectrum of the compound **6c** (100 MHz, $CDCl_3$).

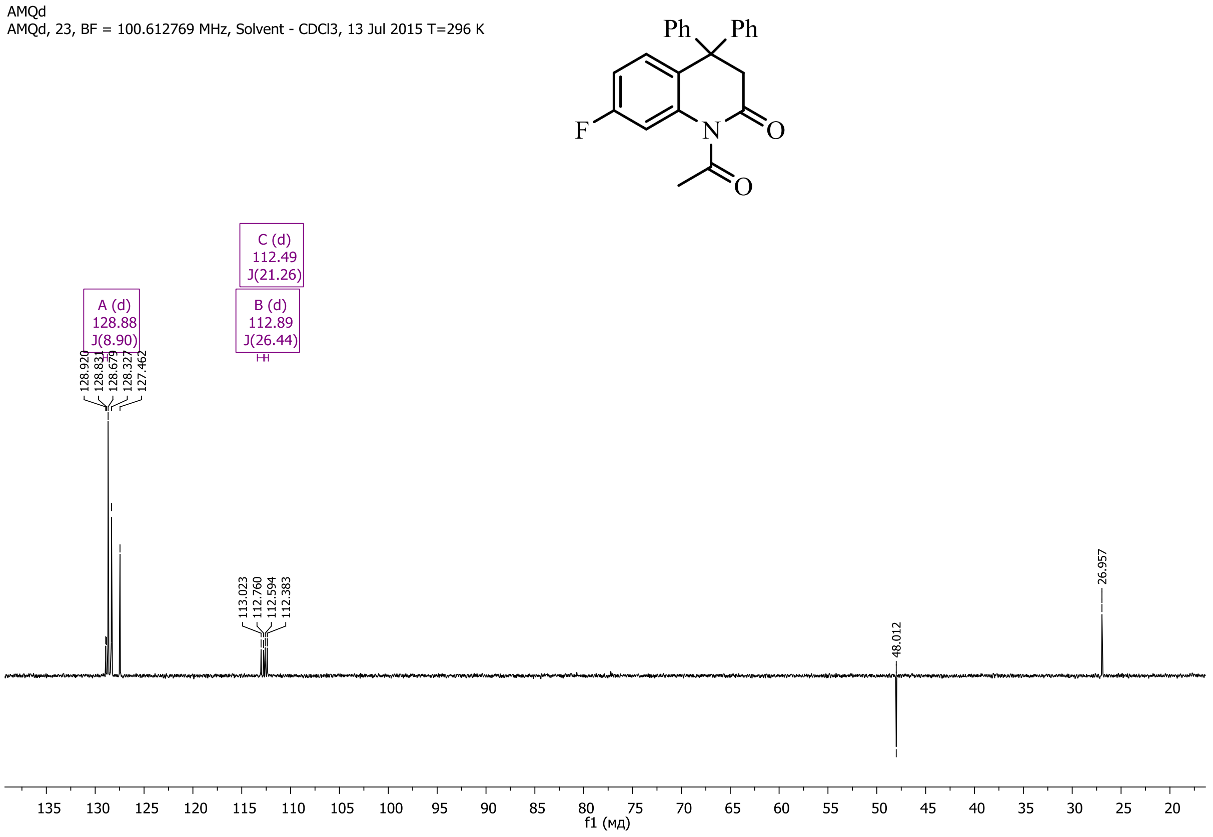

Figure S85. DEPT spectrum of the compound **6c** (100 MHz, $CDCl_3$).

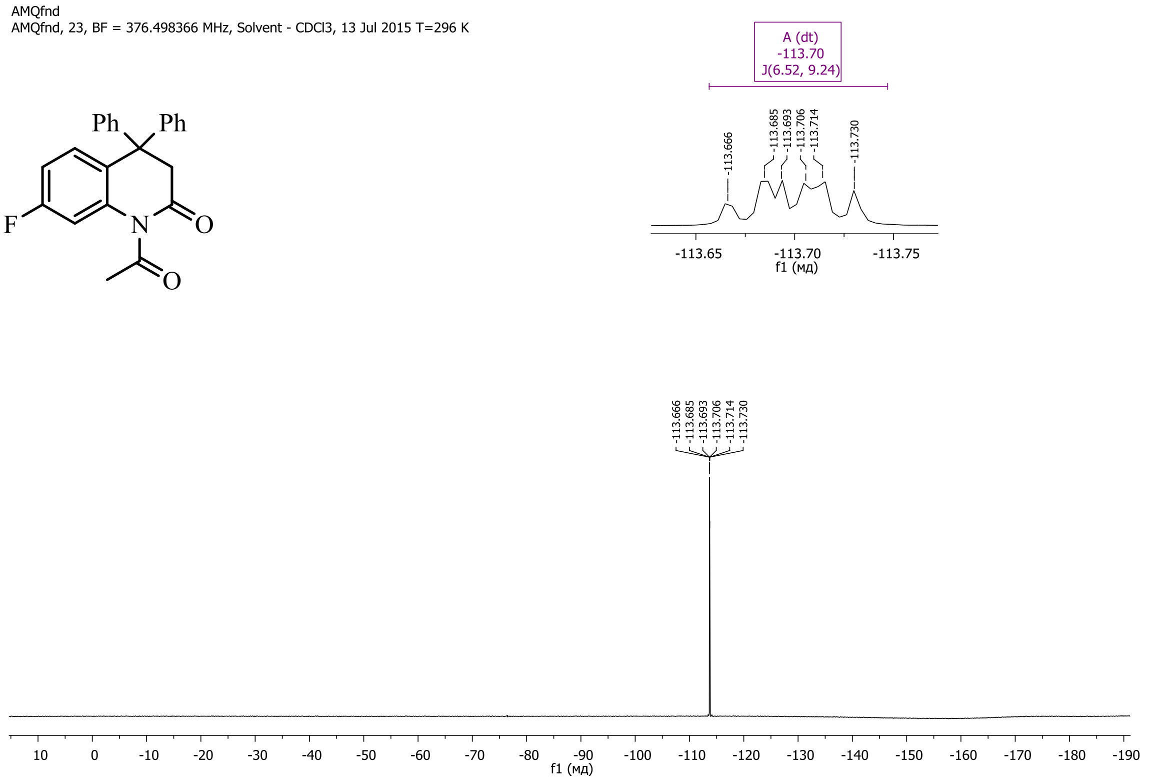

Figure S86. ^{19}F NMR spectrum of the compound **6c** (376 MHz, $CDCl_3$).

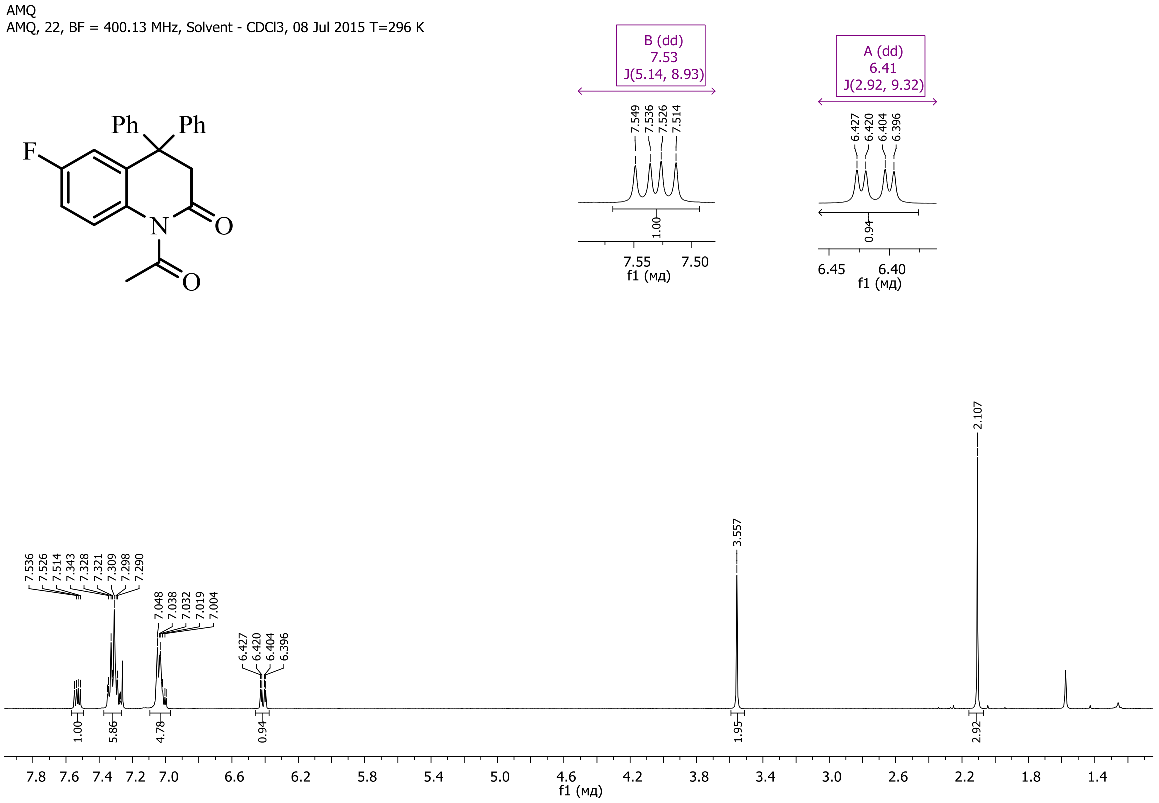

Figure S87. ^{1}H NMR spectrum of the compound **6d** (400 MHz, $CDCl_3$).

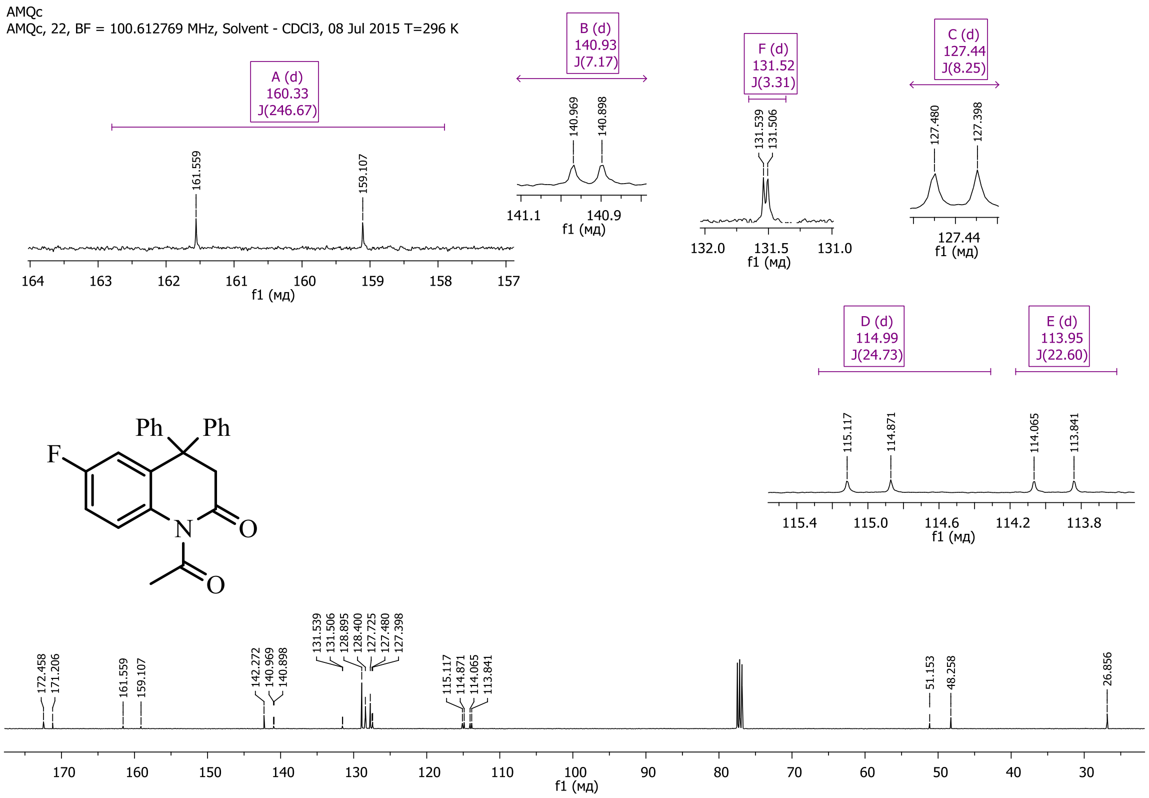

Figure S88. ^{13}C NMR spectrum of the compound **6d** (100 MHz, $CDCl_3$).

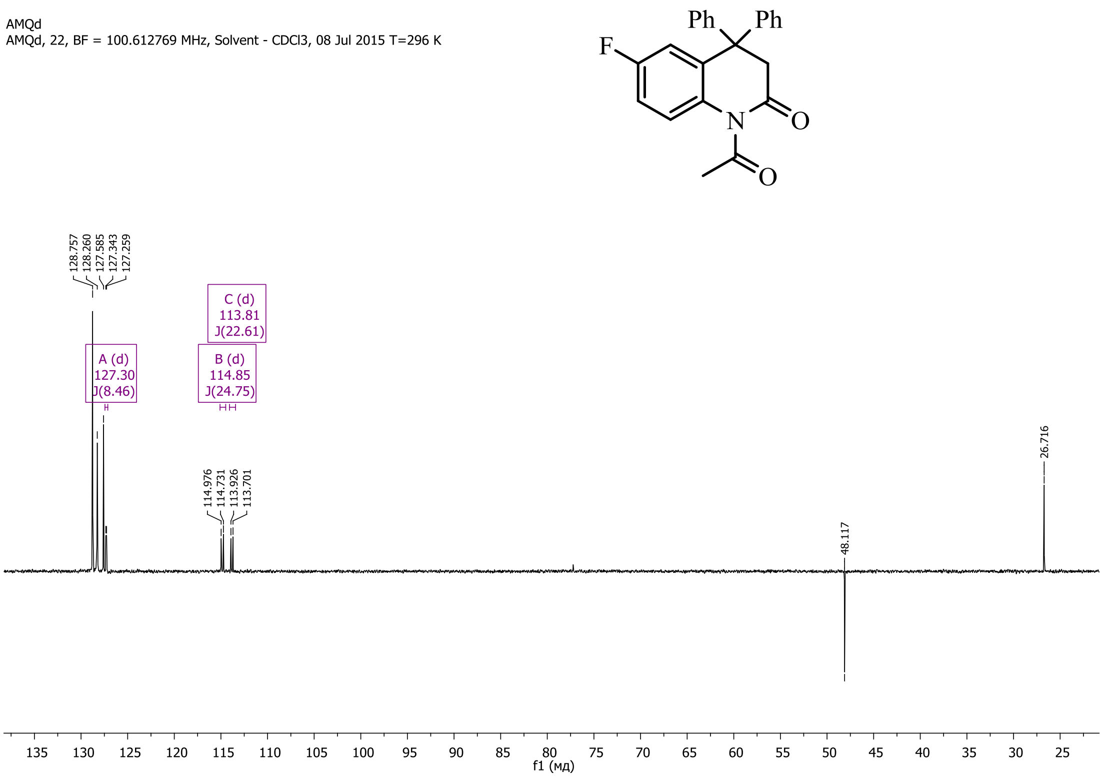

Figure S89. DEPT spectrum of the compound **6d** (100 MHz, $CDCl_3$).

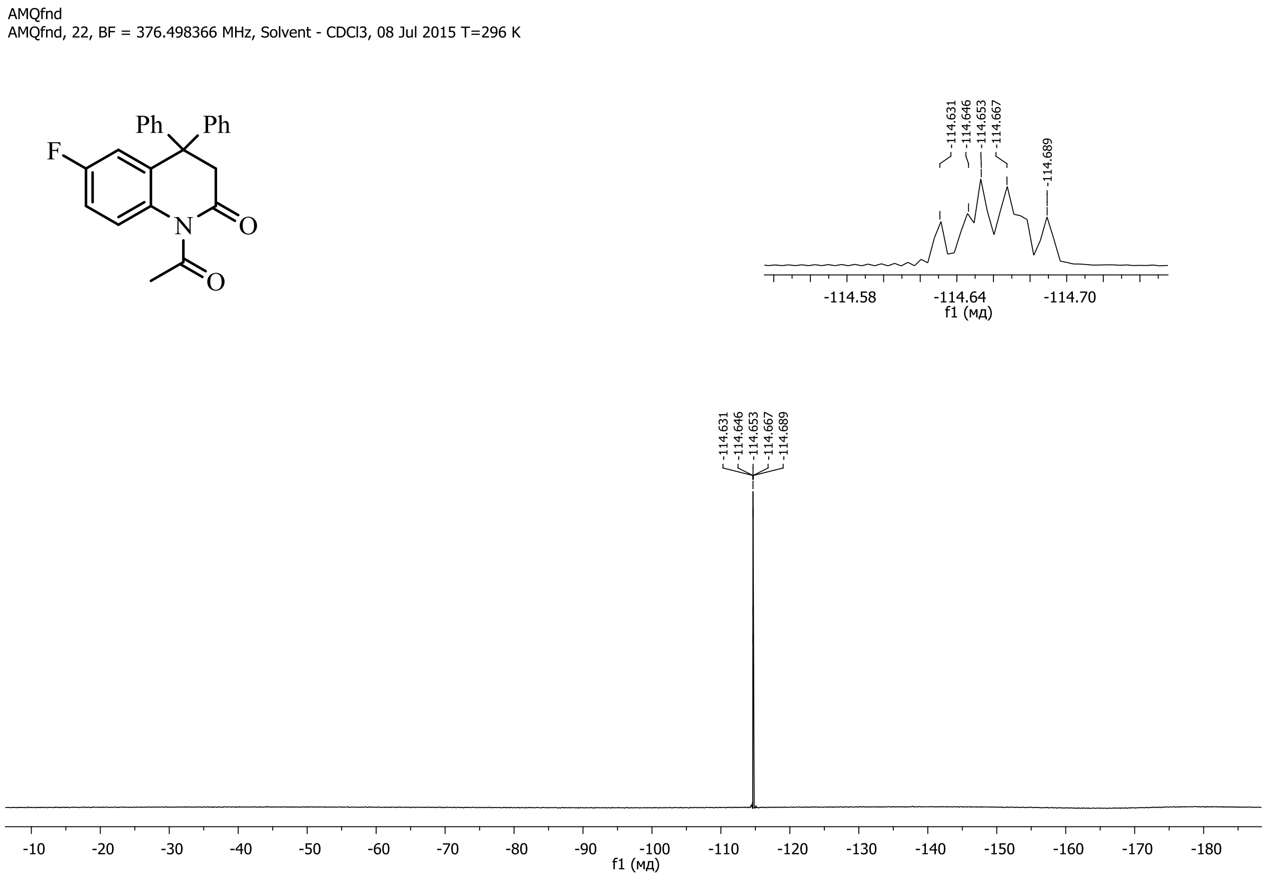

Figure S90. ^{19}F NMR spectrum of the compound **6d** (376 MHz, $CDCl_3$).

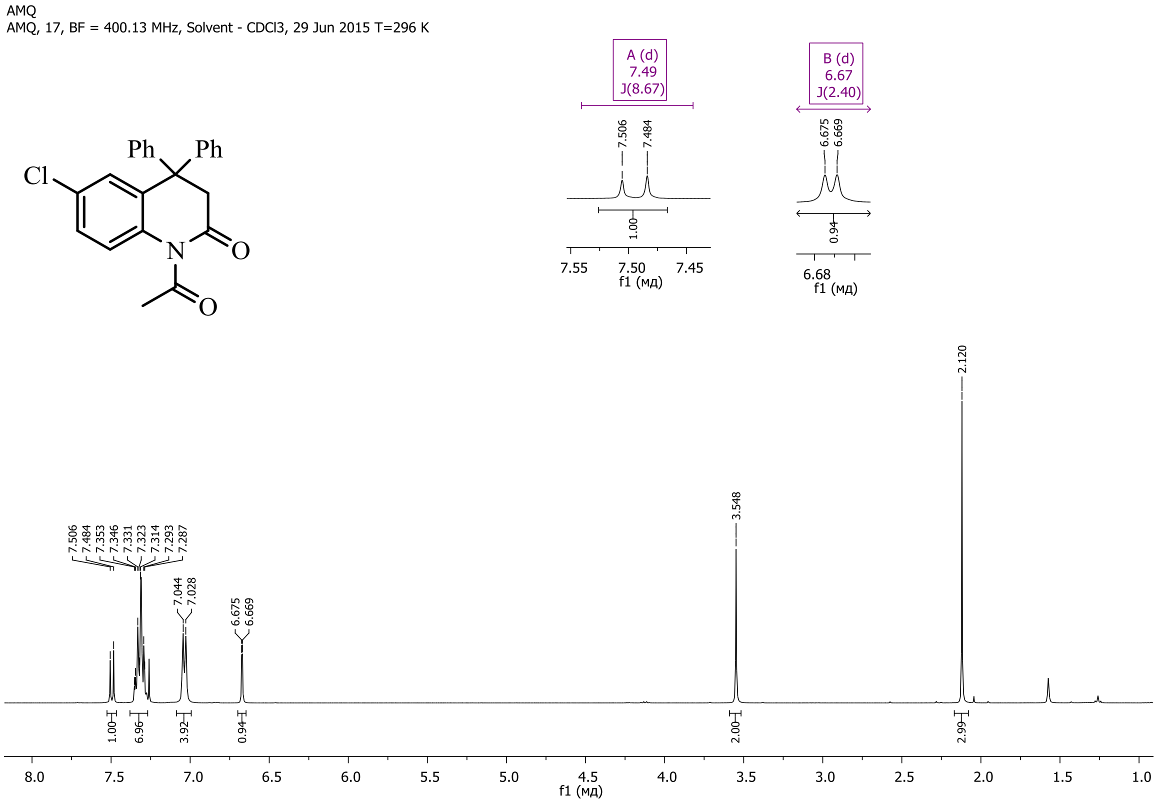

Figure S91. ^{1}H NMR spectrum of the compound **6e** (400 MHz, $CDCl_3$).

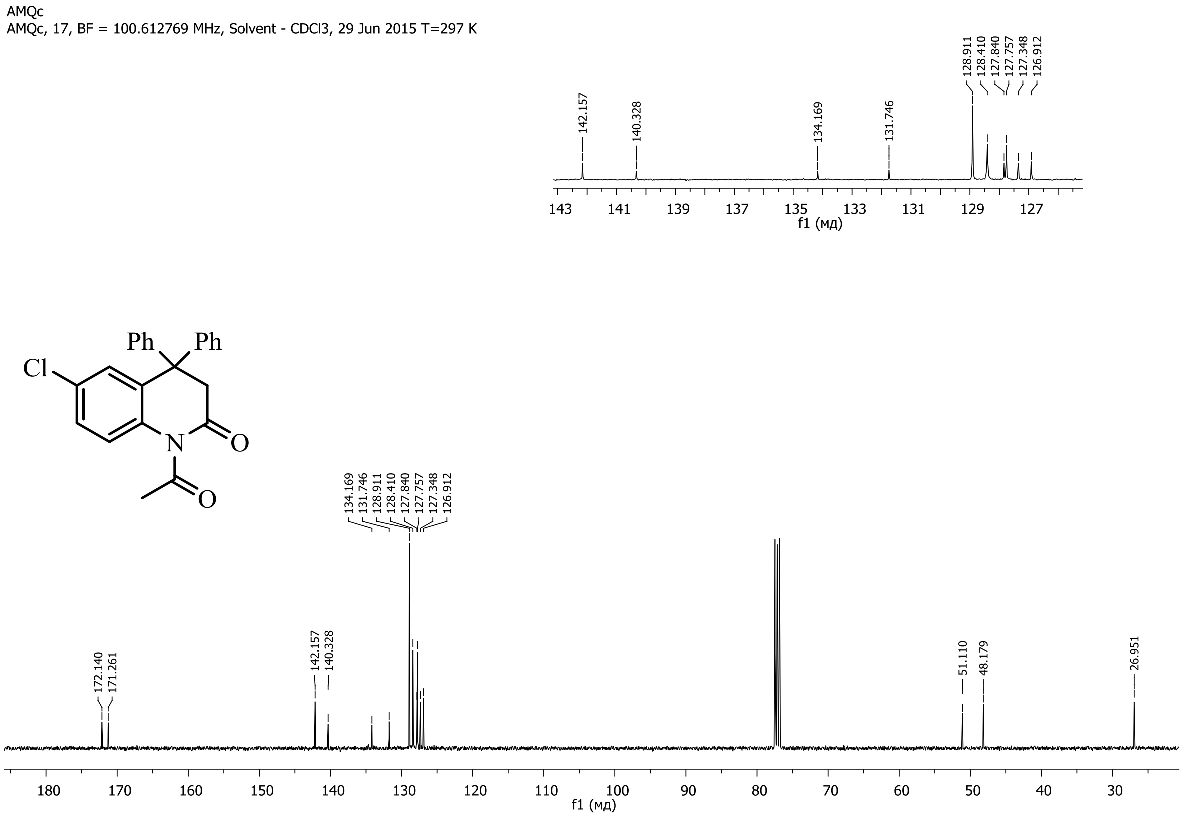

Figure S92. ^{13}C NMR spectrum of the compound **6e** (100 MHz, $CDCl_3$).

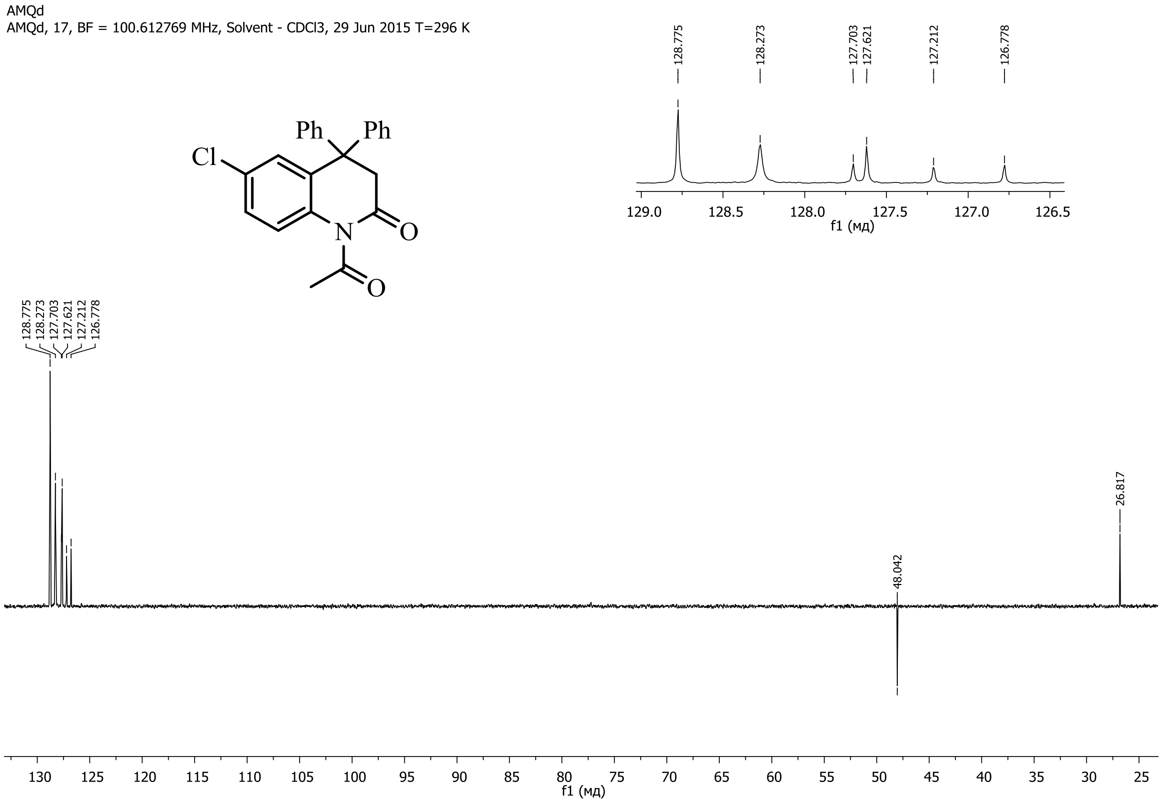

Figure S93. DEPT spectrum of the compound **6e** (100 MHz, $CDCl_3$).

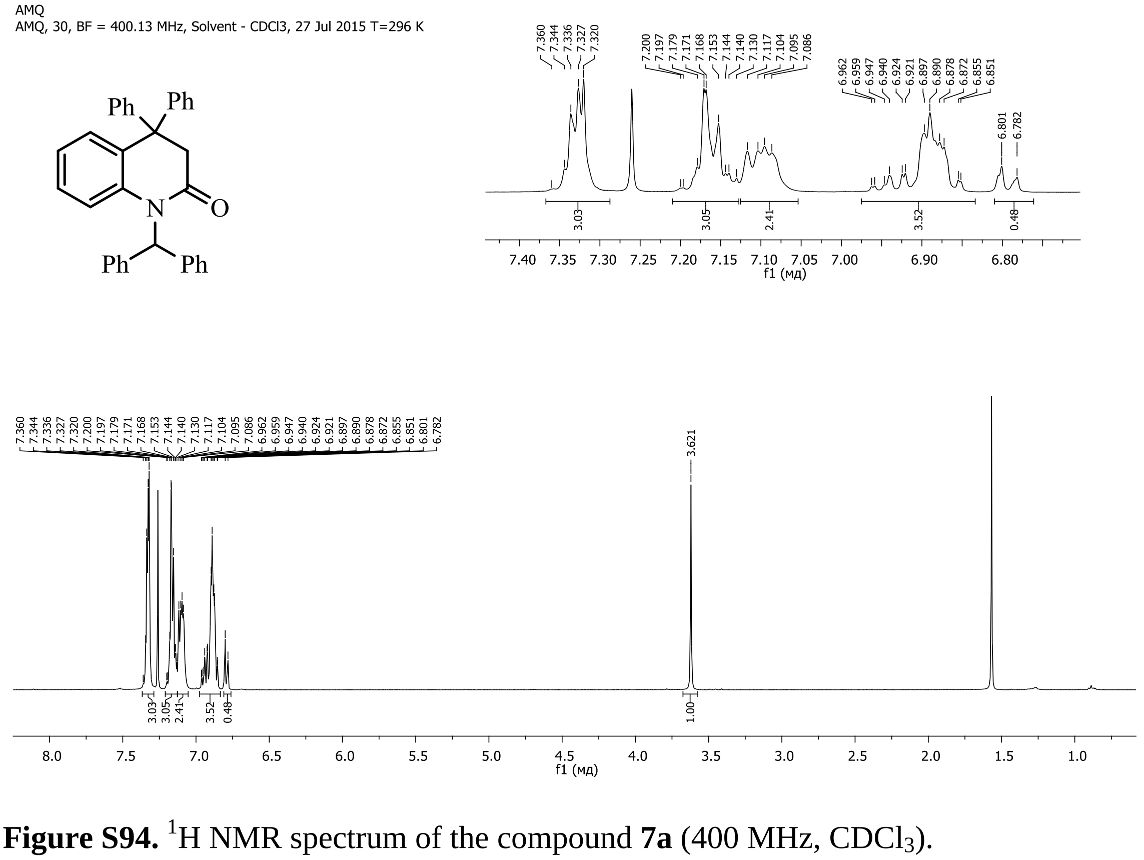

Figure S94. ^{1}H NMR spectrum of the compound **7a** (400 MHz, $CDCl_3$).

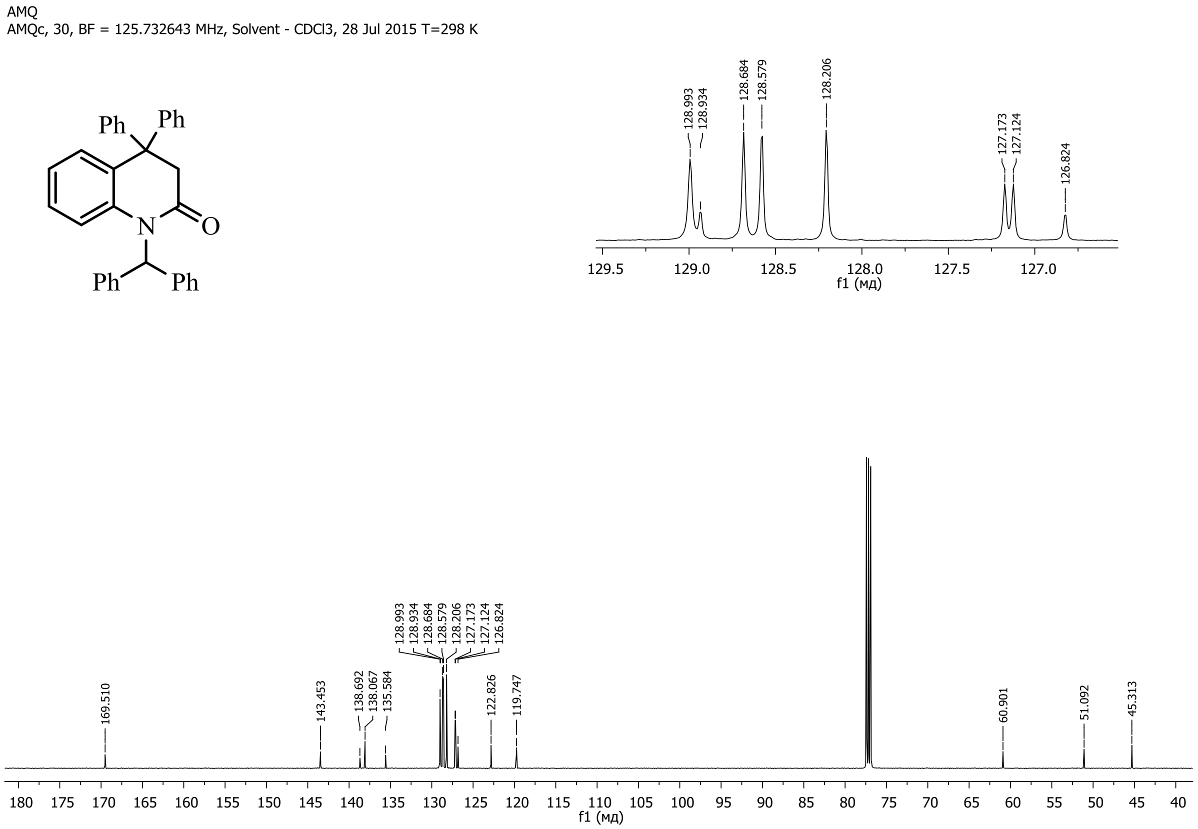

Figure S95. ^{13}C NMR spectrum of the compound **7a** (100 MHz, $CDCl_3$).

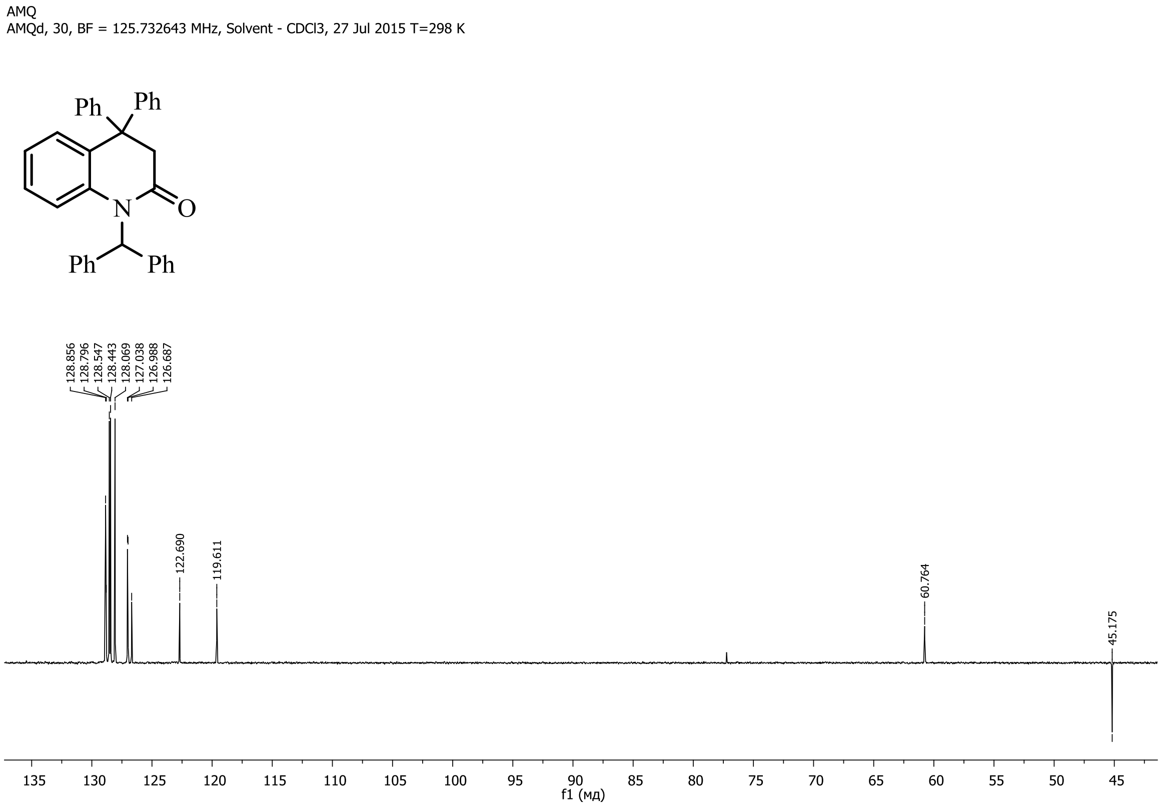

Figure S96. DEPT spectrum of the compound **7a** (100 MHz, $CDCl_3$).

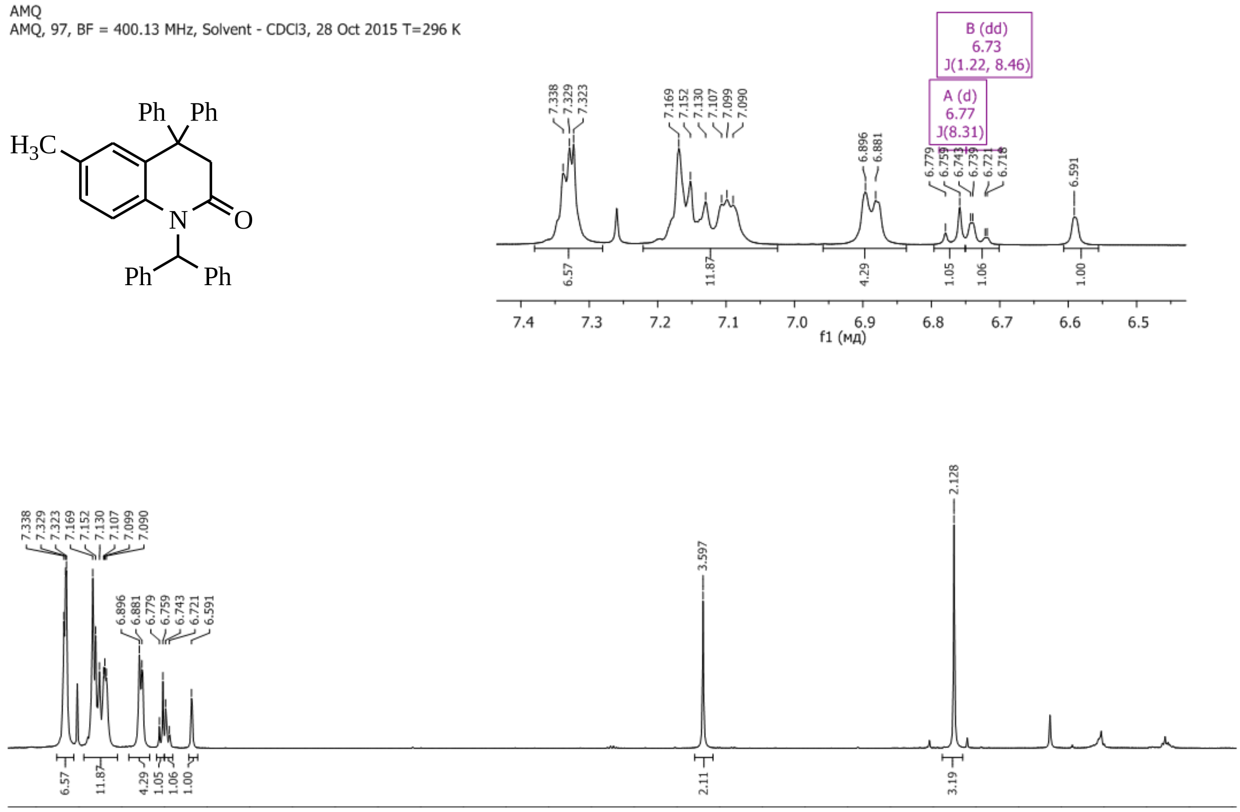

Figure S97. ^{1}H NMR spectrum of the compound **7b** (400 MHz, $CDCl_3$).

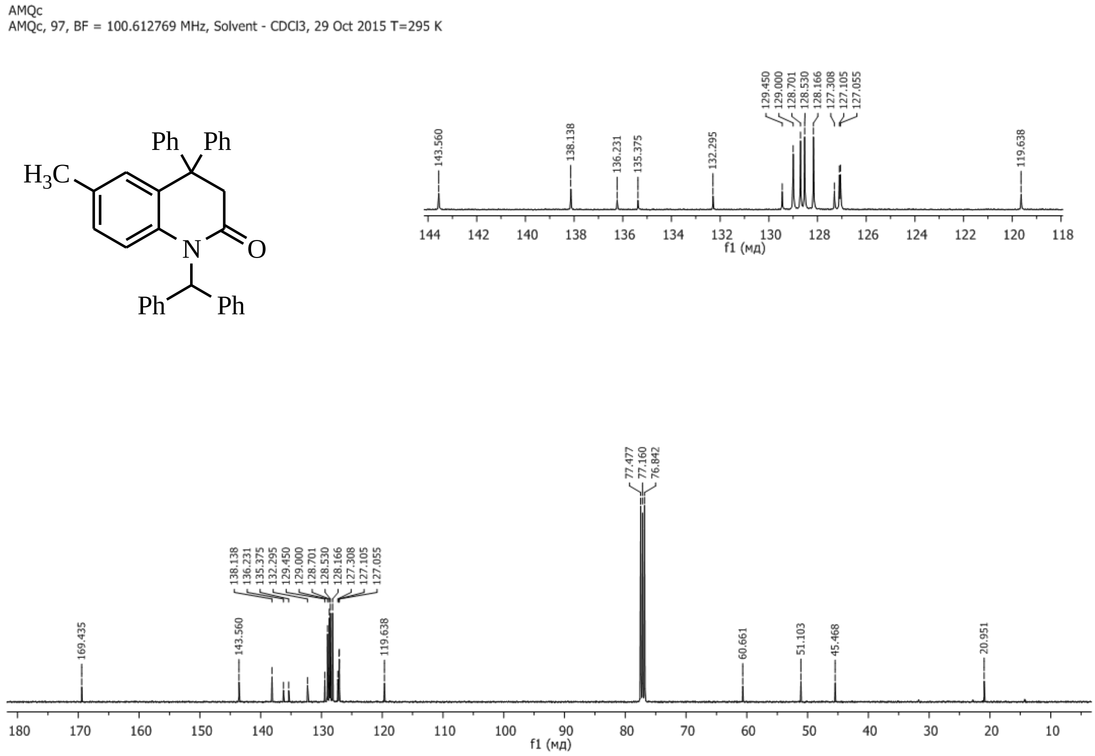

Figure S98. ^{13}C NMR spectrum of the compound **7b** (100 MHz, $CDCl_3$).

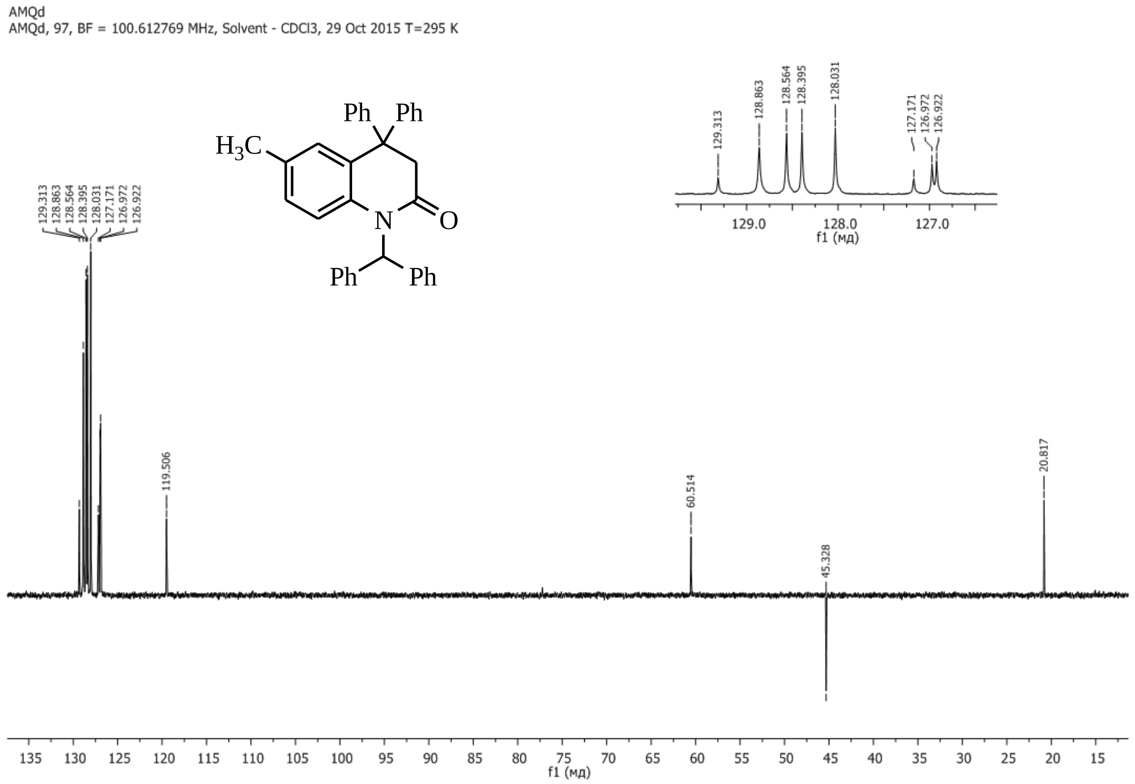

Figure S99. DEPT spectrum of the compound **7b** (100 MHz, $CDCl_3$).

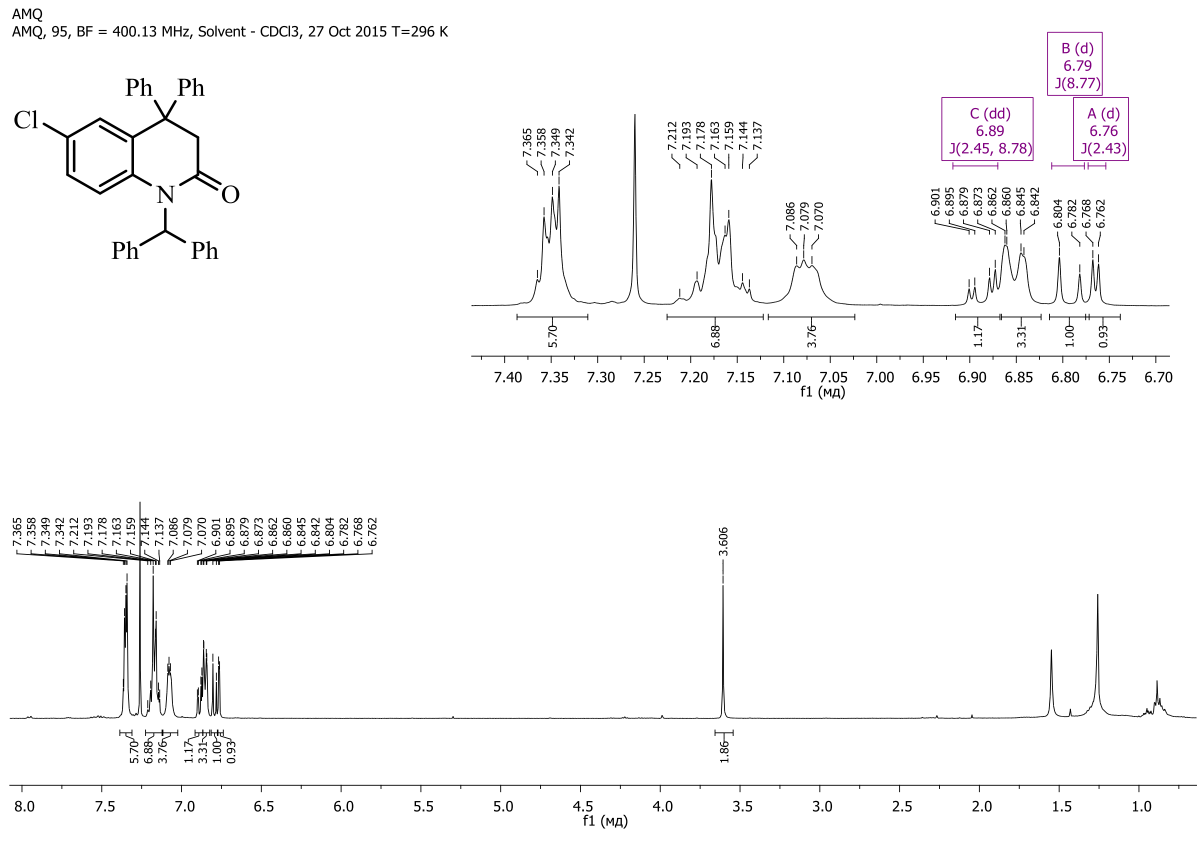

Figure S100. ^{1}H NMR spectrum of the compound **7c** (400 MHz, $CDCl_3$).

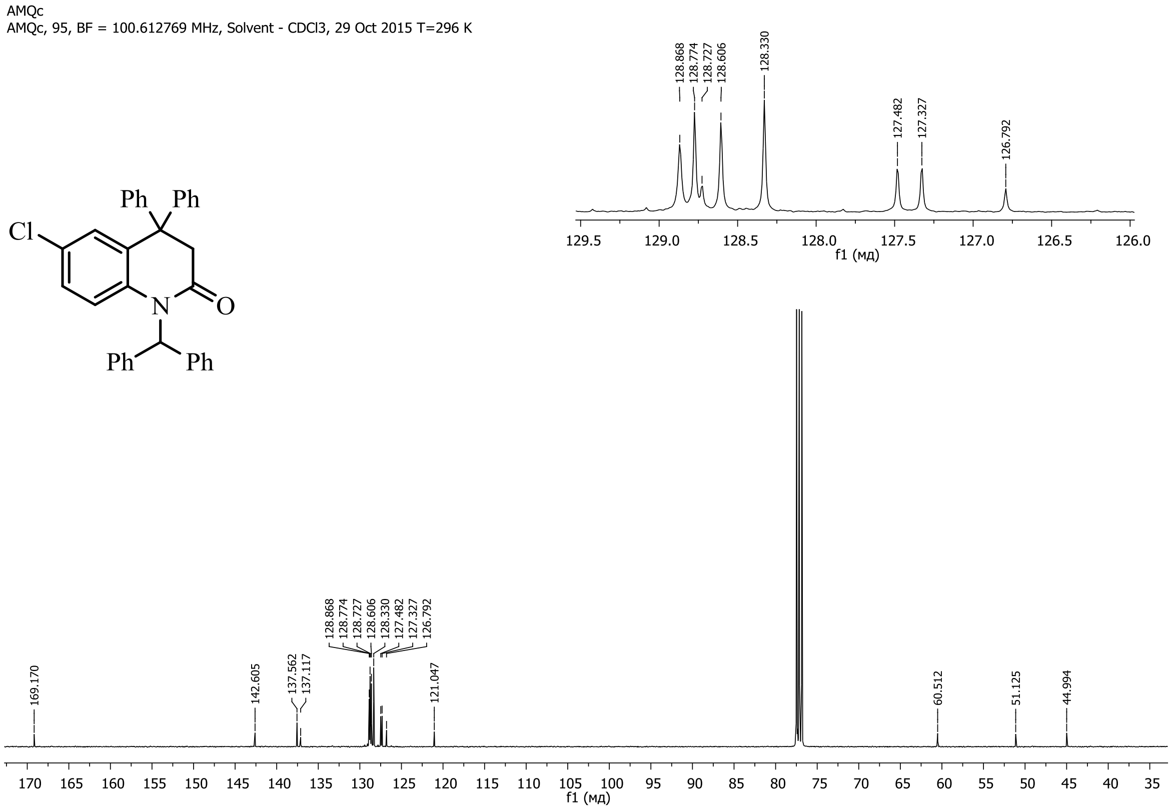

Figure S101. ^{13}C NMR spectrum of the compound **7c** (100 MHz, $CDCl_3$).

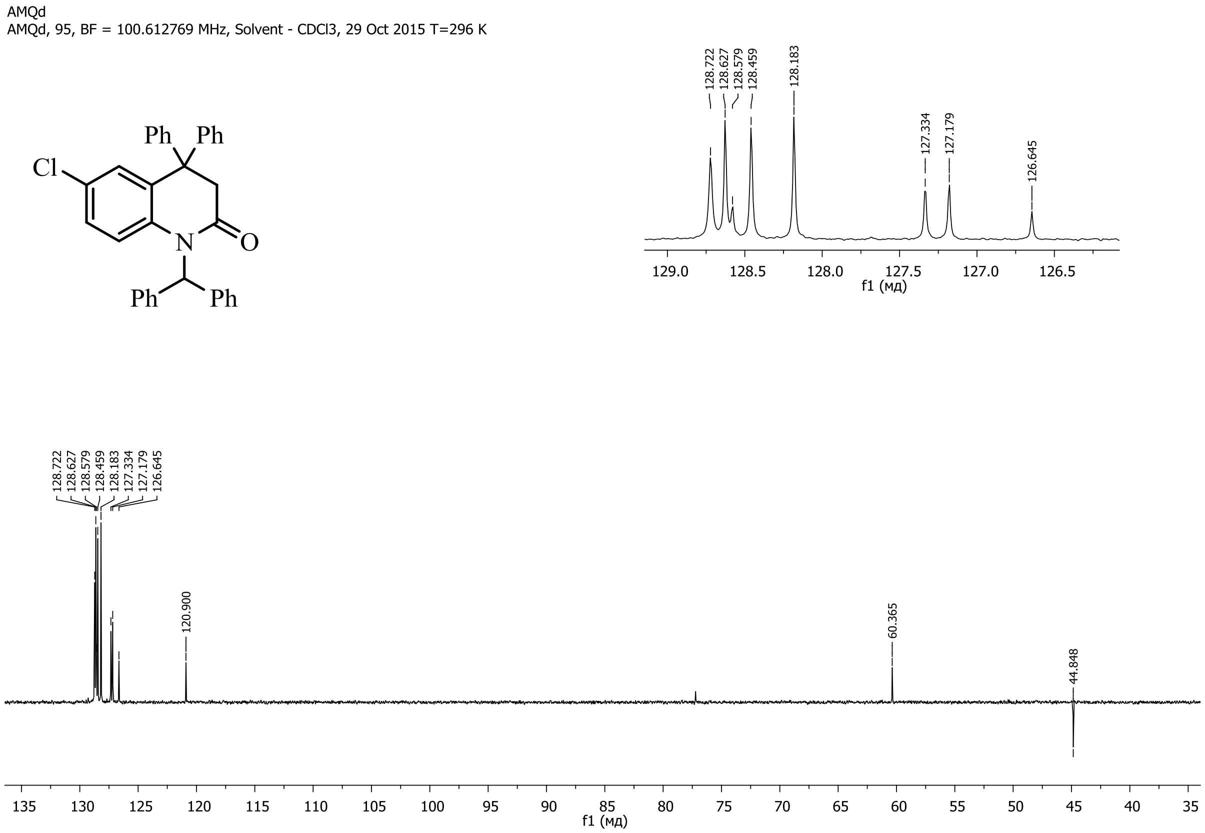

Figure S102. DEPT spectrum of the compound **7c** (100 MHz, $CDCl_3$).

Details of DFT calculations

C1

Energy **E= -1054.90802121 h,** **G^{298}= -1054.606459 h,** **μ=26.09 D**

Cartesian coordinates, Å

N	atom	x	y	z
1	C	2.417371	1.474649	0.289113
2	C	1.180800	1.268935	-0.330300
3	C	0.572117	2.355514	-0.960606
4	C	1.177393	3.608035	-0.985221
5	C	2.410275	3.798575	-0.371942
6	C	3.025055	2.726194	0.268070
7	C	0.445533	-0.090669	-0.229004
8	C	-0.507286	0.033921	0.969400
9	C	-1.894689	0.037345	0.860777
10	N	-2.529274	-0.239218	-0.441603
11	C	-1.772988	0.023174	-1.582892
12	C	-0.356926	-0.336198	-1.552450
13	C	-2.737760	0.346306	1.924124
14	C	-2.186748	0.582070	3.172199
15	C	-0.804493	0.531518	3.333953
16	C	0.013815	0.280608	2.245378
17	C	1.418773	-1.285729	-0.118863
18	C	2.456385	-1.399429	-1.051019
19	C	3.299450	-2.504185	-1.046700
20	C	3.120347	-3.519341	-0.110544
21	C	2.084185	-3.422445	0.810919
22	C	1.234816	-2.316752	0.802674
23	C	-3.688760	-0.901314	-0.495755
24	O	-4.234406	-1.155521	-1.609883
25	O	-2.266633	0.438558	-2.660008
26	H	1.086903	0.282540	2.384453
27	H	-0.361520	0.714940	4.306316
28	H	-2.836411	0.820108	4.006359
29	H	-3.811874	0.424579	1.789833
30	H	0.139005	0.081993	-2.426038
31	H	-4.130533	-1.254703	0.439601
32	H	-5.100306	-1.688745	-1.520383
33	H	-3.231502	0.779126	-2.686264
34	H	-0.419801	-1.428850	-1.715984
35	H	0.431421	-2.276691	1.526018
36	H	2.617465	-0.619701	-1.787375
37	H	1.927953	-4.208201	1.541623
38	H	4.100208	-2.568076	-1.775276
39	H	3.781767	-4.378519	-0.103059
40	H	-0.390893	2.262895	-1.447109
41	H	2.918750	0.660444	0.795006
42	H	0.680627	4.430888	-1.487175
43	H	3.984819	2.859452	0.754872
44	H	2.887167	4.772070	-0.392940

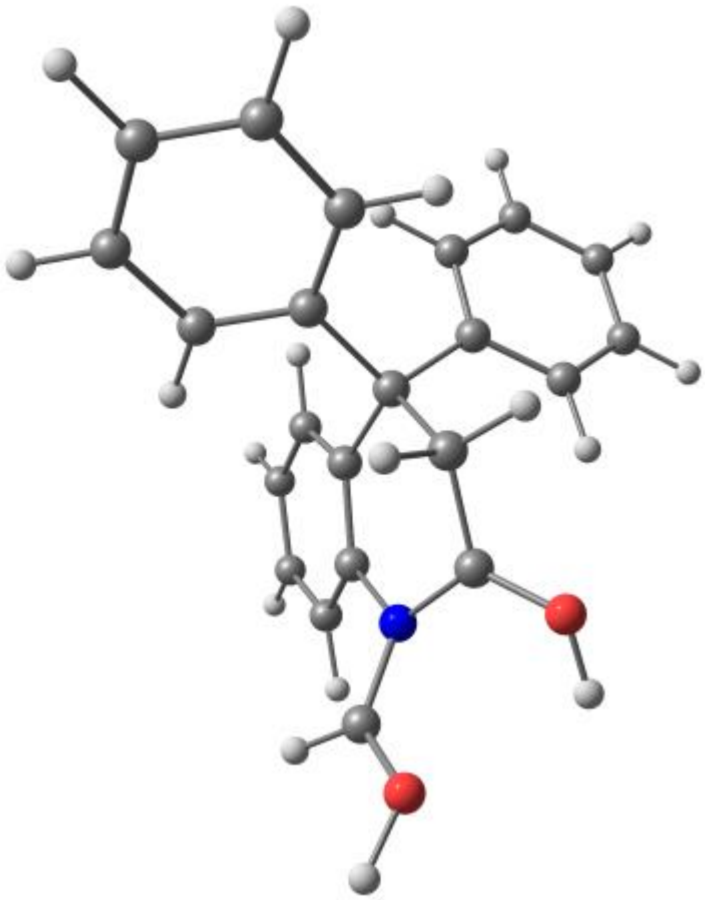

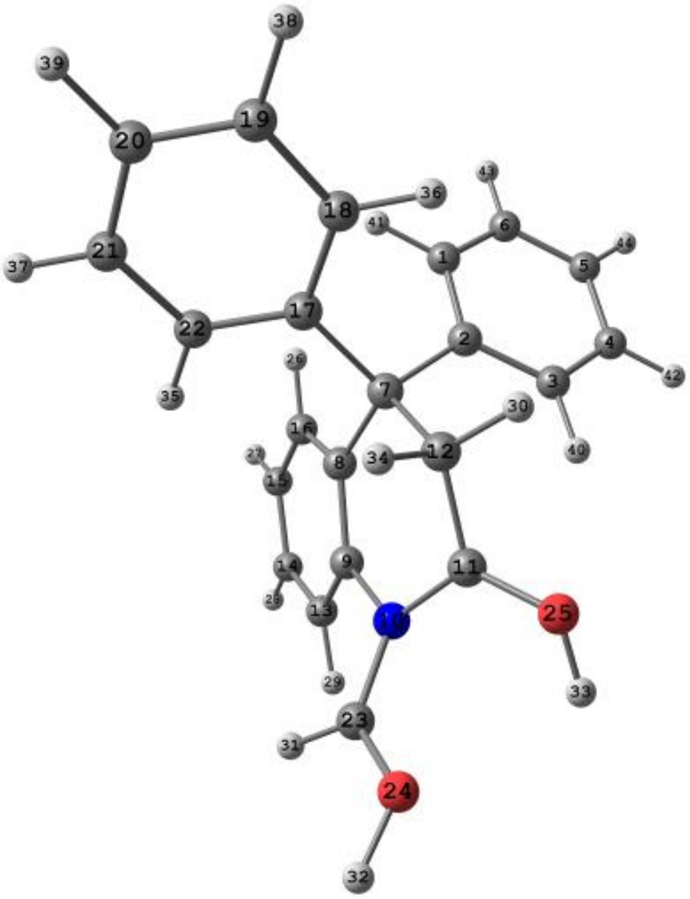

Summary of Natural Population Analysis:

Atom	No	Natural Charge	Natural Population: Core	Valence	Rydberg	Total
C	1	-0.20397	1.99901	4.18633	0.01864	6.20397
C	2	-0.03405	1.99894	4.00986	0.02525	6.03405
C	3	-0.21323	1.99900	4.19589	0.01834	6.21323
C	4	-0.17544	1.99915	4.15673	0.01956	6.17544
C	5	-0.14829	1.99915	4.13025	0.01890	6.14829
C	6	-0.15603	1.99915	4.13788	0.01900	6.15603
C	7	-0.09332	1.99903	4.05403	0.04026	6.09332
C	8	0.11019	1.99883	3.86591	0.02507	5.88981
C	9	0.05813	1.99882	3.91035	0.03270	5.94187
N	10	-0.44199	1.99913	5.41741	0.02545	7.44199
C	11	0.83772	1.99913	3.13384	0.02931	5.16228
C	12	-0.47998	1.99907	4.45655	0.02436	6.47998
C	13	-0.21978	1.99902	4.20293	0.01783	6.21978
C	14	-0.14728	1.99916	4.12812	0.02001	6.14728
C	15	-0.13101	1.99914	4.11326	0.01861	6.13101
C	16	-0.19202	1.99915	4.16063	0.03224	6.19202
C	17	-0.07230	1.99895	4.04831	0.02504	6.07230
C	18	-0.21740	1.99902	4.20033	0.01805	6.21740
C	19	-0.16766	1.99915	4.14920	0.01931	6.16766
C	20	-0.16754	1.99915	4.14933	0.01906	6.16754

C	21	-0.17515	1.99915	4.15663	0.01936	6.17515
C	22	-0.22122	1.99901	4.20352	0.01869	6.22122
C	23	0.59946	1.99934	3.37480	0.02640	5.40054
O	24	-0.58695	1.99968	6.56192	0.02535	8.58695
O	25	-0.52888	1.99962	6.50231	0.02695	8.52888
H	26	0.24482	0.00000	0.75271	0.00246	0.75518
H	27	0.24609	0.00000	0.75248	0.00143	0.75391
H	28	0.24634	0.00000	0.75218	0.00148	0.75366
H	29	0.22239	0.00000	0.77545	0.00216	0.77761
H	30	0.29258	0.00000	0.70532	0.00210	0.70742
H	31	0.24377	0.00000	0.75442	0.00181	0.75623
H	32	0.56876	0.00000	0.42860	0.00264	0.43124
H	33	0.55247	0.00000	0.44474	0.00279	0.44753
H	34	0.31900	0.00000	0.67926	0.00174	0.68100
H	35	0.21020	0.00000	0.78722	0.00258	0.78980
H	36	0.21845	0.00000	0.77945	0.00211	0.78155
H	37	0.22866	0.00000	0.76975	0.00160	0.77134
H	38	0.23193	0.00000	0.76652	0.00155	0.76807
H	39	0.23376	0.00000	0.76479	0.00145	0.76624
H	40	0.17967	0.00000	0.81726	0.00307	0.82033
H	41	0.23002	0.00000	0.76791	0.00207	0.76998
H	42	0.22905	0.00000	0.76936	0.00159	0.77095
H	43	0.23488	0.00000	0.76362	0.00150	0.76512
H	44	0.23613	0.00000	0.76244	0.00143	0.76387

===

* Total *	2.00099	49.97795	121.39977	0.62130	171.99901

Bond lengths, Å

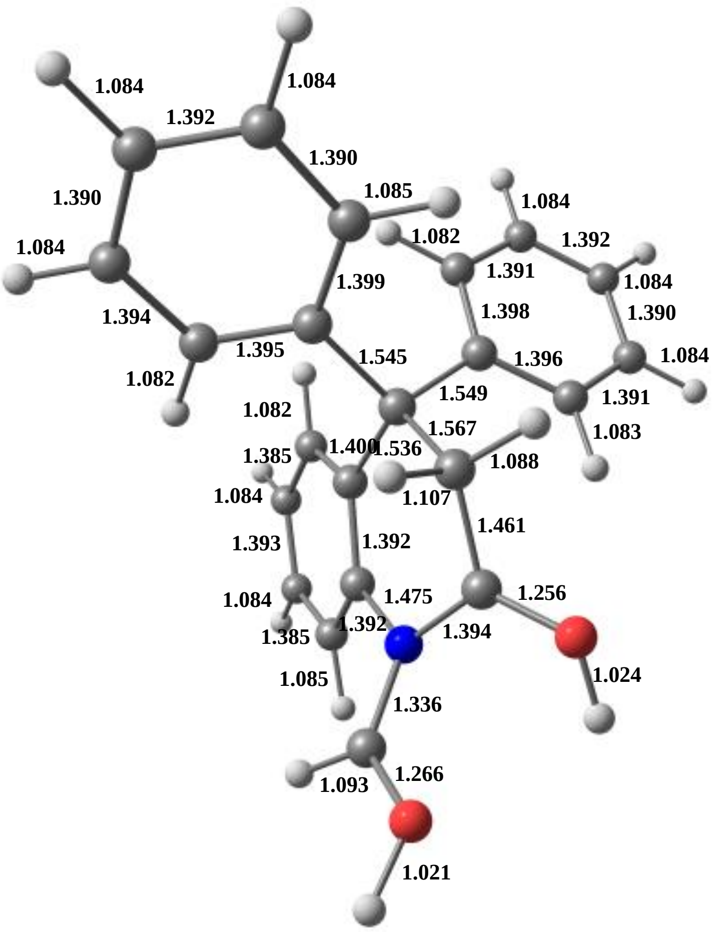
1.084
1.084
1.392
1.390
1.085
1.390
1.084
1.082
1.392
1.084
1.391
1.084
1.399
1.398
1.390
1.394
1.395
1.545
1.396
1.084
1.082
1.549
1.391
1.082
1.567
1.083
1.385
1.400
1.536
1.088
1.084
1.107
1.461
1.393
1.392
1.084
1.475
1.256
1.392
1.385
1.394
1.085
1.024
1.336
1.266
1.093
1.021

D1

Energy **E= -1094.25438712 h, G^{298}= -1093.926156 h, μ=19.27 D**

Cartesian coordinates, Å

N	atom	x	y	z
1	C	0.451461	2.533747	0.374146
2	C	0.325596	1.341103	-0.348914
3	C	-0.116378	1.412276	-1.672565
4	C	-0.440473	2.637318	-2.252257
5	C	-0.326274	3.812819	-1.518997
6	C	0.124976	3.756247	-0.203518
7	C	0.716774	-0.006857	0.298449
8	C	0.030253	-1.174798	-0.414360
9	C	-1.309117	-1.443254	-0.116428
10	N	-2.019894	-0.551981	0.789222
11	C	-1.315500	0.111704	1.745056
12	C	0.148245	-0.067224	1.761517
13	C	-1.988904	-2.545188	-0.618084
14	C	-1.335060	-3.379414	-1.515428
15	C	-0.018484	-3.110079	-1.870485
16	C	0.660811	-2.029350	-1.311203
17	C	2.245647	-0.196628	0.370622
18	C	2.791047	-1.234619	1.135749
19	C	4.165529	-1.429548	1.199225
20	C	5.025886	-0.592355	0.492847
21	C	4.495721	0.434602	-0.278154
22	C	3.117028	0.632115	-0.337812
23	C	-3.335638	-0.209026	0.485060
24	O	-4.209641	-0.060906	1.382930
25	O	-1.968330	0.911811	2.486753
26	C	-3.749006	0.060589	-0.904389
27	H	1.696291	-1.857593	-1.575249
28	H	0.497211	-3.757606	-2.570934
29	H	-1.852055	-4.242127	-1.919064
30	H	-3.002543	-2.770475	-0.298309
31	H	0.337287	-1.058226	2.191747
32	H	-3.997180	-0.413128	2.313491
33	H	-1.421210	1.423270	3.172432
34	H	0.591740	0.675139	2.423224
35	H	-0.200362	0.517375	-2.275451
36	H	0.828134	2.529105	1.391655
37	H	-0.781641	2.665547	-3.281260
38	H	0.229449	4.665622	0.377863
39	H	-0.582107	4.765551	-1.969025
40	H	2.151692	-1.917923	1.684469
41	H	2.735721	1.440137	-0.945112
42	H	4.562706	-2.239310	1.801192
43	H	5.151414	1.092294	-0.838010
44	H	6.098633	-0.742120	0.543756
45	H	-4.427358	-0.728809	-1.242203

46	H	-4.331686	0.986256	-0.887511
47	H	-2.905943	0.138574	-1.580195

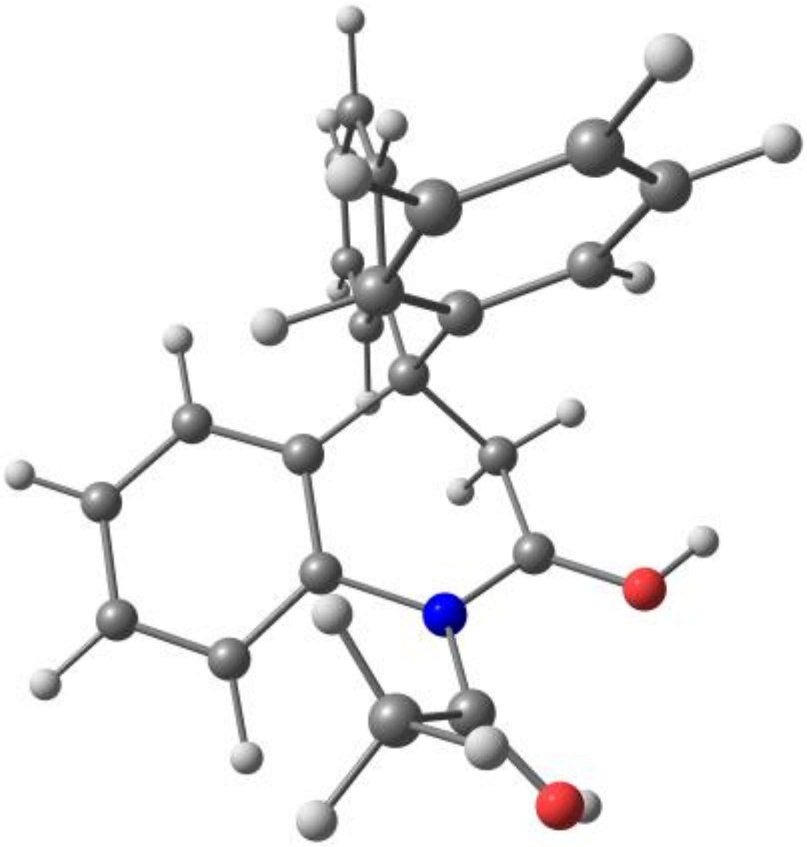

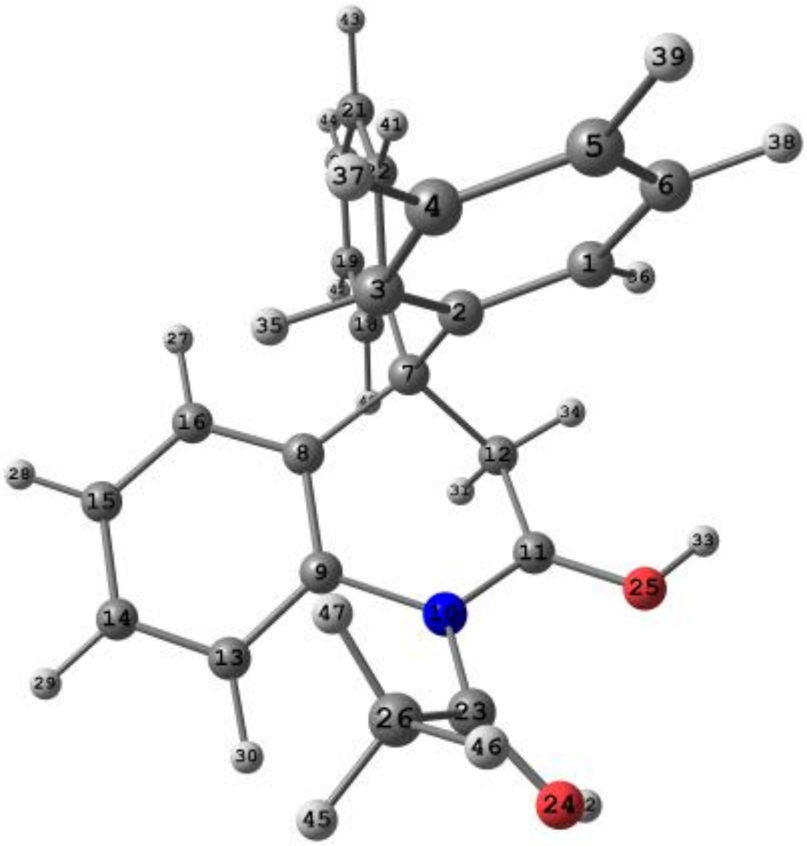

Summary of Natural Population Analysis:

Atom	No	Natural Charge	Natural Population Core	Valence	Rydberg	Total
C	1	-0.23119	1.99901	4.21306	0.01912	6.23119
C	2	-0.07014	1.99896	4.04462	0.02656	6.07014
C	3	-0.22477	1.99900	4.20666	0.01910	6.22477
C	4	-0.17414	1.99915	4.15555	0.01945	6.17414
C	5	-0.16962	1.99915	4.15122	0.01925	6.16962
C	6	-0.17178	1.99915	4.15304	0.01959	6.17178
C	7	-0.06328	1.99899	4.03365	0.03064	6.06328
C	8	-0.05694	1.99896	4.01623	0.04175	6.05694
C	9	0.22848	1.99874	3.74865	0.02413	5.77152
N	10	-0.49749	1.99908	5.46374	0.03467	7.49749
C	11	0.82285	1.99912	3.15032	0.02772	5.17715
C	12	-0.47328	1.99910	4.45066	0.02352	6.47328
C	13	-0.24971	1.99913	4.21347	0.03711	6.24971
C	14	-0.17023	1.99915	4.15191	0.01917	6.17023
C	15	-0.12026	1.99915	4.10265	0.01845	6.12026
C	16	-0.16539	1.99901	4.14616	0.02023	6.16539
C	17	-0.06967	1.99895	4.04615	0.02456	6.06967

C	18	-0.21695	1.99902	4.19994	0.01799	6.21695
C	19	-0.17296	1.99915	4.15441	0.01940	6.17296
C	20	-0.16313	1.99915	4.14498	0.01900	6.16313
C	21	-0.16923	1.99915	4.15106	0.01902	6.16923
C	22	-0.18814	1.99901	4.17112	0.01800	6.18814
C	23	0.84307	1.99927	3.12873	0.02892	5.15693
O	24	-0.53325	1.99963	6.50617	0.02746	8.53325
O	25	-0.60047	1.99964	6.57562	0.02522	8.60047
C	26	-0.67432	1.99919	4.65779	0.01735	6.67432
H	27	0.25066	0.00000	0.74729	0.00204	0.74934
H	28	0.24464	0.00000	0.75390	0.00145	0.75536
H	29	0.24992	0.00000	0.74845	0.00163	0.75008
H	30	0.22606	0.00000	0.77173	0.00221	0.77394
H	31	0.29256	0.00000	0.70549	0.00195	0.70744
H	32	0.54953	0.00000	0.44790	0.00256	0.45047
H	33	0.56412	0.00000	0.43325	0.00263	0.43588
H	34	0.26332	0.00000	0.73453	0.00215	0.73668
H	35	0.21721	0.00000	0.78033	0.00245	0.78279
H	36	0.20224	0.00000	0.79532	0.00243	0.79776
H	37	0.22953	0.00000	0.76890	0.00158	0.77047
H	38	0.23112	0.00000	0.76729	0.00159	0.76888
H	39	0.23500	0.00000	0.76352	0.00148	0.76500
H	40	0.19891	0.00000	0.79877	0.00232	0.80109
H	41	0.22505	0.00000	0.77241	0.00254	0.77495
H	42	0.23079	0.00000	0.76765	0.00156	0.76921
H	43	0.23217	0.00000	0.76631	0.00152	0.76783
H	44	0.23494	0.00000	0.76361	0.00144	0.76506
H	45	0.29508	0.00000	0.70327	0.00165	0.70492
H	46	0.29392	0.00000	0.70450	0.00159	0.70608
H	47	0.26751	0.00000	0.73004	0.00245	0.73249

===

* Total *	2.00234	51.97701	127.36205	0.65860	179.99766

Bond lengths, Å

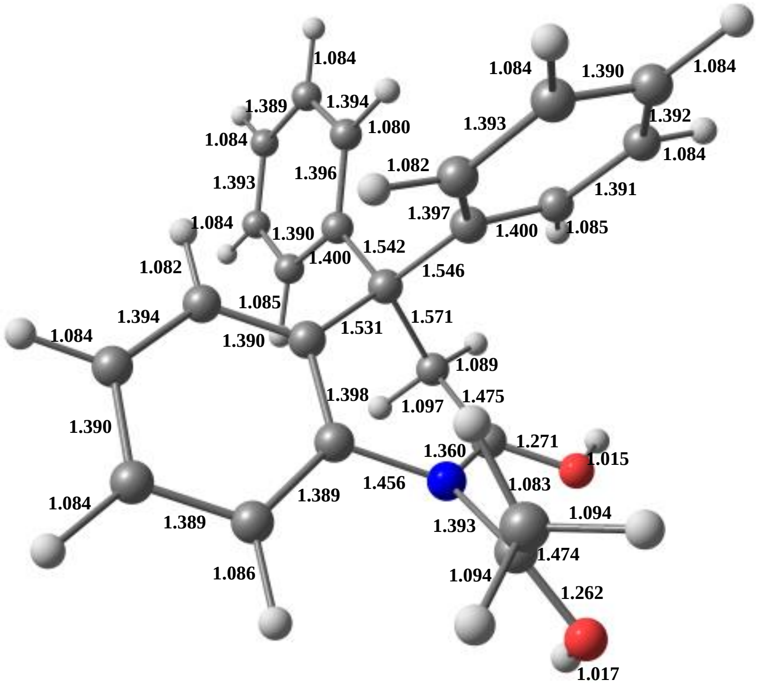
1.084
1.084
1.390
1.084
1.389
1.394
1.080
1.392
1.084
1.393
1.084
1.393
1.396
1.082
1.084
1.391
1.084
1.397
1.390
1.400
1.085
1.082
1.400
1.542
1.546
1.394
1.085
1.084
1.390
1.531
1.571
1.089
1.398
1.097
1.475
1.390
1.360
1.271
1.015
1.084
1.456
1.083
1.389
1.389
1.393
1.094
1.474
1.086
1.094
1.262
1.017

References

S1. Saulnier, S.; Golovanov, A. A.; Ivanov, A. Yu.; Boyarskaya, I. A.; Vasilyev, A. V. *J. Org. Chem.*, **2016**, *81*, 1967–1980. doi: 10.1021/acs.joc.5b02785

S2. Dolomanov, O. V.; Bourhis, L. J.; Gildea, R. J.; Howard, J. A. K.; Puschmann, H. *J. Appl. Cryst*. **2009**, *42*, 339-341. doi:10.1107/S0021889808042726

S3. *SHELXS*; Sheldrick, G. M. *Acta Cryst.* **2008**, *A64*, 112-122. doi:10.1107/S0108767307043930

S4. Frisch, M. J.; Trucks, G. W.; Schlegel, H. B.; Scuseria, G. E.; Robb, M. A.; Cheeseman, J. R.; Montgomery Jr., J. A.; Vreven, T.; Kudin, K. N.; Burant, J. C.; Millam, J. M.; Iyengar, S. S.; Tomasi, J.; Barone, V.; Mennucci, B.; Cossi, M.; Scalmani, G.; Rega, N.; Petersson, G. A.; Nakatsuji, H.; Hada, M.; Ehara, M.; Toyota, K.; Fukuda, R.; Hasegawa, J.; Ishida, M.; Nakajima, T.; Honda, Y.; Kitao, O.; Nakai, H.; Klene, M.; Li, X.; Knox, J. E.; Hratchian, H. P.; Cross, J. B.; Adamo, C.; Jaramillo, J.; Gomperts, R.; Stratmann, R. E.; Yazyev, O.; Austin, A. J.; Cammi, R.; Pomelli, C.; Ochterski, J. W.; Ayala, P. Y.; Morokuma, K.; Voth, G. A.; Salvador, P.; Dannenberg, J. J.; Zakrzewski, V. G.; Dapprich, S.; Daniels, A. D.; Strain, M. C.; Farkas, O.; Malick, D. K.; Rabuck, D.; Raghavachari, K.; Foresman, J. B.; Ortiz, J. V.; Cui, Q.; Baboul, A. G.; Clifford, S.; Cioslowski, J.; Stefanov, B. B.; Liu, G.; Liashenko, A.; Piskorz, P.; Komaromi, I.; Martin, R. L.; Fox, D. J.; Keith, T.; Al-Laham, M. A.; Peng, C. Y.; Nanayakkara, A.; Challacombe, M.; Gill, P. M. W.; Johnson, B.; Chen, W.; Wong, M. W.; Gonzalez, C.; Pople, J. A. *GAUSSIAN 03, Revision D.02*; Gaussian, Inc., Wallingford CT, **2004**.

S5. Ryabukhin, D. S.; Gurskaya, L. Yu.; Fukin, G. K.; Vasilyev, A. V. *Tetrahedron* **2014**, *70*, 6428-6443. doi:10.1016/j.tet.2014.07.028

S6. Ryabukhin, D. S.; Vasilyev, A. V. *Tetrahedron Lett.* **2015**, *56*, 2200-2202. doi:10.1016/j.tetlet.2015.03.060

S7. Bogachenkov, A. S.; Dogadina, A. V.; Boyarskaya, I. A.; Boyarskiy, V. P; Vasilyev, A. V. *Org. Biomol. Chem.*, **2016**, *14*, 1370–1381. doi: 10.1039/C5OB02143J

S8. Ryabukhin, D. S.; Vasilyev, A. V.; Vyazmin, S. Yu. *Russ. Chem. Bull.* **2012**, *44*, 843-846. doi: 10.1007/s11172-012-0117-3

S9. Ryabukhin, D. S.; Vasilyev, A. V. *Russ. J. Org. Chem.* **2008**, *44*, 1849-1851. doi: 10.1134/S1070428008120257

S10. Han, C. Y.-P.; Song, X.-R. ; Qiu, Y.-F.; Hao, X.-H.; Wang, J.; Wu, X.-X.; Liu, X.-Y.; Liang, Y.-M. *J. Org. Chem.* **2015**, *80*, 9200−9207. doi: 10.1021/acs.joc.5b01633
